# Mechanochemical Grignard Reactions with Gaseous CO_2_ and Sodium Methyl Carbonate**

**DOI:** 10.1002/anie.202116514

**Published:** 2022-01-19

**Authors:** Victoria S. Pfennig, Romina C. Villella, Julia Nikodemus, Carsten Bolm

**Affiliations:** ^1^ Institute of Organic Chemistry RWTH Aachen University Landoltweg 1 52074 Aachen Germany

**Keywords:** Ball milling, Carbon dioxide, Carboxylation, Grignard reaction, Mechanochemistry

## Abstract

A one‐pot, three‐step protocol for the preparation of Grignard reagents from organobromides in a ball mill and their subsequent reactions with gaseous carbon dioxide (CO_2_) or sodium methyl carbonate providing aryl and alkyl carboxylic acids in up to 82 % yield is reported. Noteworthy are the short reaction times and the significantly reduced solvent amounts [2.0 equiv. for liquid assisted grinding (LAG) conditions]. Unexpectedly, aryl bromides with methoxy substituents lead to symmetric ketones as major products.

## Graphical abstract

Grignard‐type reactions have been performed in ball mills providing carboxylic acids from organobromides with gaseous CO_2_ or sodium methyl carbonate (SMC) as C1‐synthon. The transformations are solvent‐free and occur in the presence of LiOH under liquid‐assisted grinding (LAG) conditions with 2 equivalents of 2‐MeTHF as ethereal additive.

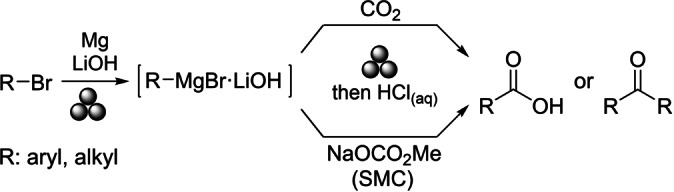

The increasing use of carbon dioxide (CO_2_) as C1‐synthon in organic chemistry is driven by the urge to avoid fossil resources and out‐dated, dangerous synthetic procedures or reagents such as phosgene.^[1]^ Mechanochemistry is experiencing growing popularity, and it has been employed for a plethora of organic and organometallic syntheses.[^2^, ^3^] Some major attributes of mechanochemical or ball milling approaches are reduced amounts of solvents, shorter reaction times through higher reaction rates, and alternative reaction pathways that are unavailable in solution. Specific ball milling setups allow the use of gases as reactants.^[4]^ While the mechanochemical hydrogenation^[5]^ or formation of CO_2_ as a by‐product have been observed and addressed in several investigations,^[6]^ examples of mechanochemical carboxylative CO_2_ insertions are restricted to two studies: the transformation of aziridine into oxazolidinones using dry ice^[7]^ and the addition of gaseous CO_2_ to l‐lysine forming its ϵ‐carbamate (Scheme 1, a and b).[^8^, ^9^]

**Scheme 1:**
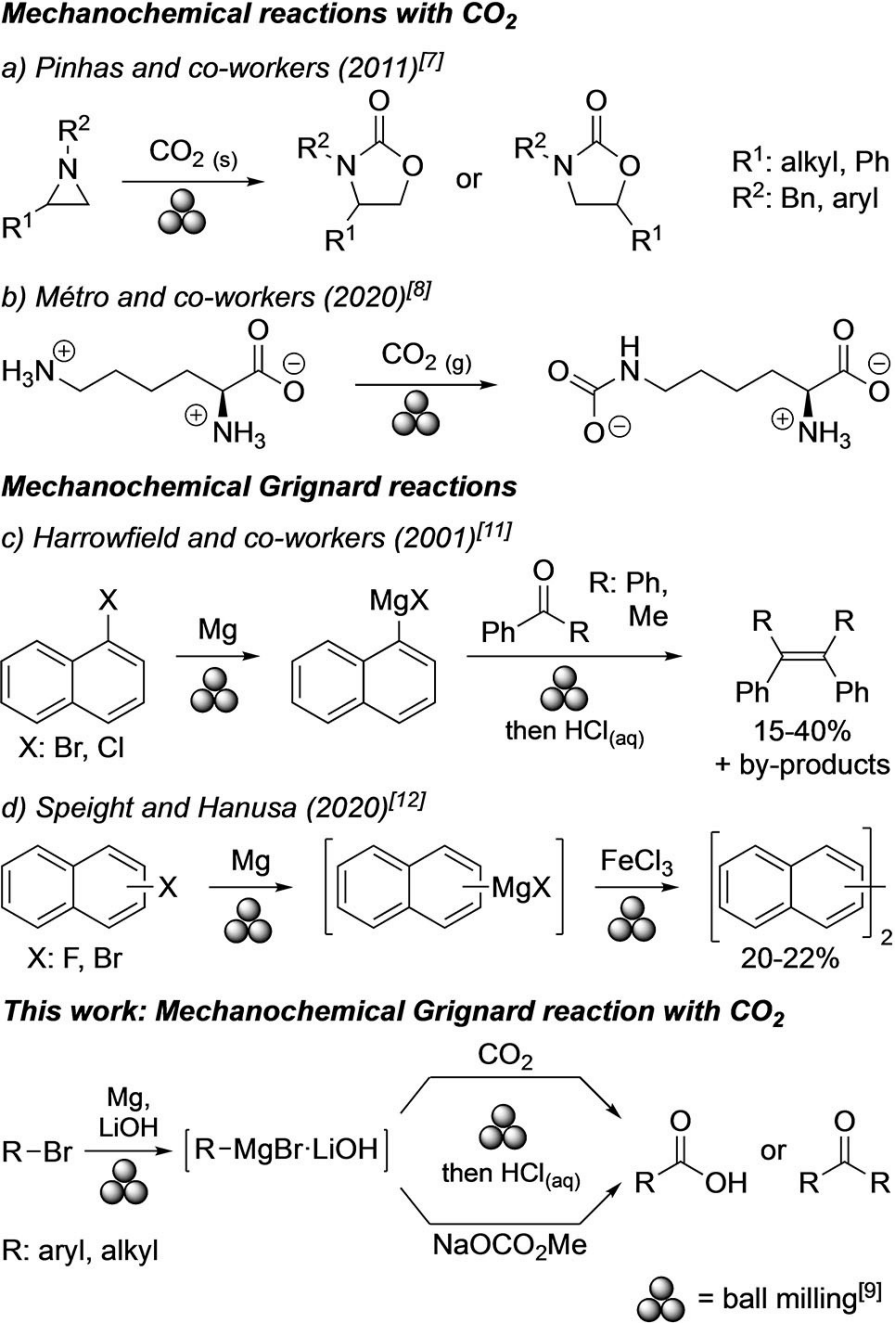
Previously reported mechanochemical reactions with CO_2_ (a and b),[^7^, ^8^] mechanochemical Grignard reactions (c and d),[^11^, ^12^] and mechanochemically conducted Grignard reactions with CO_2_ in this work.

Grignard reagents prepared from organohalides allow to form C−C bonds with electrophiles. The use of CO_2_ as electrophile provides carboxylic acids. Although 120 years have passed since Victor Grignard reported the insertion of magnesium into a C−X bond,^[10]^ its potential in mechanochemistry has not been fully exploited yet. Mechanochemical adaptions of Grignard reactions essentially halted at the attempt to isolate solvent‐free, reactive organomagnesiums by Harrowfield et al. (Scheme 1, c).^[11]^ Their experiments required an excess of magnesium to obtain a manipulable powder that could readily be removed from the milling vessel. When scavenging the Grignard reagents with ketones, however, this excess magnesium promoted the formation of the respective alkenes through McMurry‐type reactions besides the anticipated tertiary alcohols and other by‐products. In search of a mechanochemical way to conduct Grignard reactions that hardly occur in solution, Speight and Hanusa found that ball milling facilitates the insertion of magnesium into a C−F bond as detected by the respective binaphthyls, albeit in low yields (Scheme 1, d).[^12^, ^13^]

We hypothesized that conducting a Grignard reaction under mechanochemical conditions would bring along a number of advantages such as the possibility of conducting the reaction with negligible amounts of potentially dangerous solvents or the continuous activation of magnesium by grinding and thus removal of reacted surfaces. Realizing the potential and challenges, we initiated a program to, first, prepare Grignard reagents in a ball mill and, second, to react the expected organomagnesium reagents with CO_2_ under mechanochemical conditions. As equipment we used a ZrO_2_‐M milling vessel with two gas valves for adding gaseous reactants or inert gases, which is commercially available. The Grignard carboxylations were conducted in three separate steps: First, magnesium turnings were milled to transform them into a fine powder. Then, the Grignard reagent was generated after adding an organobromide. For these two initial steps, the reagents were added to the open vessels in air and then, the latter were closed and flushed with argon gas before milling. Last, gaseous CO_2_ was introduced through the gas valves to serve as electrophile in the final milling step.^[14]^ After terminating the milling, dilute hydrochloric acid was added to facilitate the removal of the product mixture from the milling vessel. Finally, extraction with ethyl acetate afforded the crude carboxylic acid.

In the very first experiment, combining all three steps by milling 4‐tolyl bromide (**1 a**) with magnesium turnings under a CO_2_ atmosphere at 600 rpm for 90 min did not yield any 4‐toluic acid (**2 a**; Table 1, entry 2). Activating the magnesium turnings in a separate milling step prior to the addition of **1 a** generated acid **2 a**, albeit in only trace amounts (entry 3). Probably, this extra step enlarged surface areas and removed passivated metal surfaces, as previously suggested for flow‐chemistry setups of magnesium insertions into aryl halide bonds and for the industrial scale production of Grignard reagents.[^3^, ^15^, ^16^]

**Table 1:** Effect of changing the optimal reaction conditions of the mechanochemical Grignard reaction of **1 a** with CO_2_ in a planetary ball mill.^[a]^

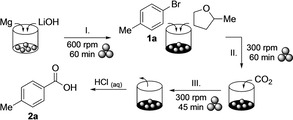		
Entry	Modified conditions	Yield [%] **2 a** ^[b]^
1	none	63
2	combining all three steps in one; no THF; no Li‐salt	0
3	combining II. and III.; no THF, no Li‐salt	4
4	using THF without any lithium salt	25
5	using THF instead of 2‐MeTHF	65^[c]^
6	using THF and LiCl	60
7	drying of the milling vessel and balls in advance	56
8	omitting Ar during II.	14

[a] Reactions conditions (4.0 mmol scale with respect to **1 a**); step I=20 mL ZrO_2_‐M milling vessel with gas inlet/outlet valves and 5 ZrO_2_‐M balls (Ø 10 mm); Mg (2.5 equiv.), LiOH (1.1 equiv.) under Ar; step 2=addition of 2‐MeTHF (2.0 equiv.) and **1 a** (4.0 mmol) under Ar; step III=CO_2_ (4 bar). [b] Determined after column chromatography. [c] Average over two experiments (due to reproducibility issues when THF was used); for more details, see Supporting Information.

Although many mechanochemical reactions are solvent‐free,^[17]^ trace amounts of solvents often significantly affect the reaction rates. Such liquid‐assisted grinding (LAG) conditions can be quantified by the parameter *η* [*η*=*V* (solvent in μL)/*m* (reagents in mg)].^[18]^ Thus here, 2 equiv. of THF were added to the reaction mixture (corresponding to *η*=0.64 μL mg^−1^). As a result, the yield of **2 a** increased to 25 % (Table 1, entry 4).^[19]^ Probably, this positive effect of the additive THF was due to a stabilization of the organometallic intermediate by the Lewis basic ether as observed by Grignard himself,^[10a]^ further investigated by Schlenk and Schlenk,^[20]^ as well as others.[^21^, ^22^]

Among various ethereal additives, only 2‐methyltetrahydrofuran (2‐MeTHF) performed as well as THF (Table 1, entries 1 and 5).^[23]^ In comparison to THF, 2‐MeTHF offered several advantages including its production from renewable biomass (furfural or levulinic acid), its larger range of possible reaction temperatures at which it remains liquid, and its superior performance in various organometallic reactions.^[24]^ In the context of the study reported here, the finding by Kadam et al. was of particular interest, as they described 2‐MeTHF as a superior alternative to diethyl ether and THF in Grignard reactions, highlighting its ability to suppress Wurtz couplings of benzyl halides.^[25]^ Furthermore, a recent study uncovered that its thermodynamic properties make 2‐MeTHF a safer solvent for the formation of Grignard reagents than THF as it prevents thermal runaway reactions.^[26]^ Consequently, 2‐MeTHF was the preferred additive in the later discussed evaluation of the substrate scope.

Guided by results reported by Knochel and co‐workers on “Turbo‐Grignard reagents”,^[27]^ we investigated the effect of lithium chloride as additive in the first grinding step. This protocol modification increased the yield of **2 a** from 25 % to 60 % (Table 1, entries 4 and 6). Surprisingly, a screening of lithium salts^[23]^ revealed LiOH to be superior over LiCl (63 % of **2 a**, Table 1, entry 1). To the best of our knowledge, this observation is unprecedented, and we attribute this improvement by hydroxide to the unusual reaction conditions lacking standard interactions between a possible magnesiate and the surrounding solvent.

Drying of the grinding vessel and the balls (apart from the lid and valve material containing low‐melting plastics) by keeping it at 100 °C overnight prior to use had almost no effect on the yield of **2 a** (56 %; Table 1, entry 7). In contrast, flushing the milling vessel with argon after the addition of **1 a** and the ethereal solvent proved crucial. Omitting this step and performing the magnesium insertion reaction in ambient atmosphere caused the formation of various side products, and the yield of **2 a** dropped to 14 % (Table 1, entry 8).^[28]^

Next, the substrate scope was investigated (Scheme 2). Applying the optimal reaction conditions to other aryl bromides gave similar yields for the sterically and electronically related 2‐methyl‐ and 4‐trifluoromethyl‐substituted acids **2 b** and **2 c** (67 % and 71 %, respectively). The number of electron‐withdrawing halogen substituents on the phenyl ring had little to no effect on the yield of the acids. Thus, 4‐chlorobenzoic acid (**2 d**) was obtained in 49 % yield, compared to 51 % for both 4‐fluoro‐ and perfluorobenzoic acids (**2 e** and **2 i**, respectively). In the series of compounds with electron‐donating substituents, sterically hindered 2,4,6‐tri‐*iso*‐propylphenyl bromide (**1 j**) underwent the carboxylation to the respective acid **2 j** in 52 % yield, while 4‐*tert*‐butylphenyl bromide (**1 f**) formed 42 % of 4‐*tert*‐butylbenzoic acid (**2 f**). The presence of 4‐ and 2‐(methylthio) groups led to yields of 58 % for 4‐(methylthio)benzoic acid (**2 g**) and 67 % for 2‐(methylthio)benzoic acid (**2 h**). Using a naphthyl group instead of substituted phenyls generated 2‐naphthoic acid (**2 n**) in 44 % yield. 2‐Thiophenecarboxylic acid (**2 m**) was obtained in 56 % yield from 2‐bromothiophene (**1 m**).^[29]^

**Scheme 2:**
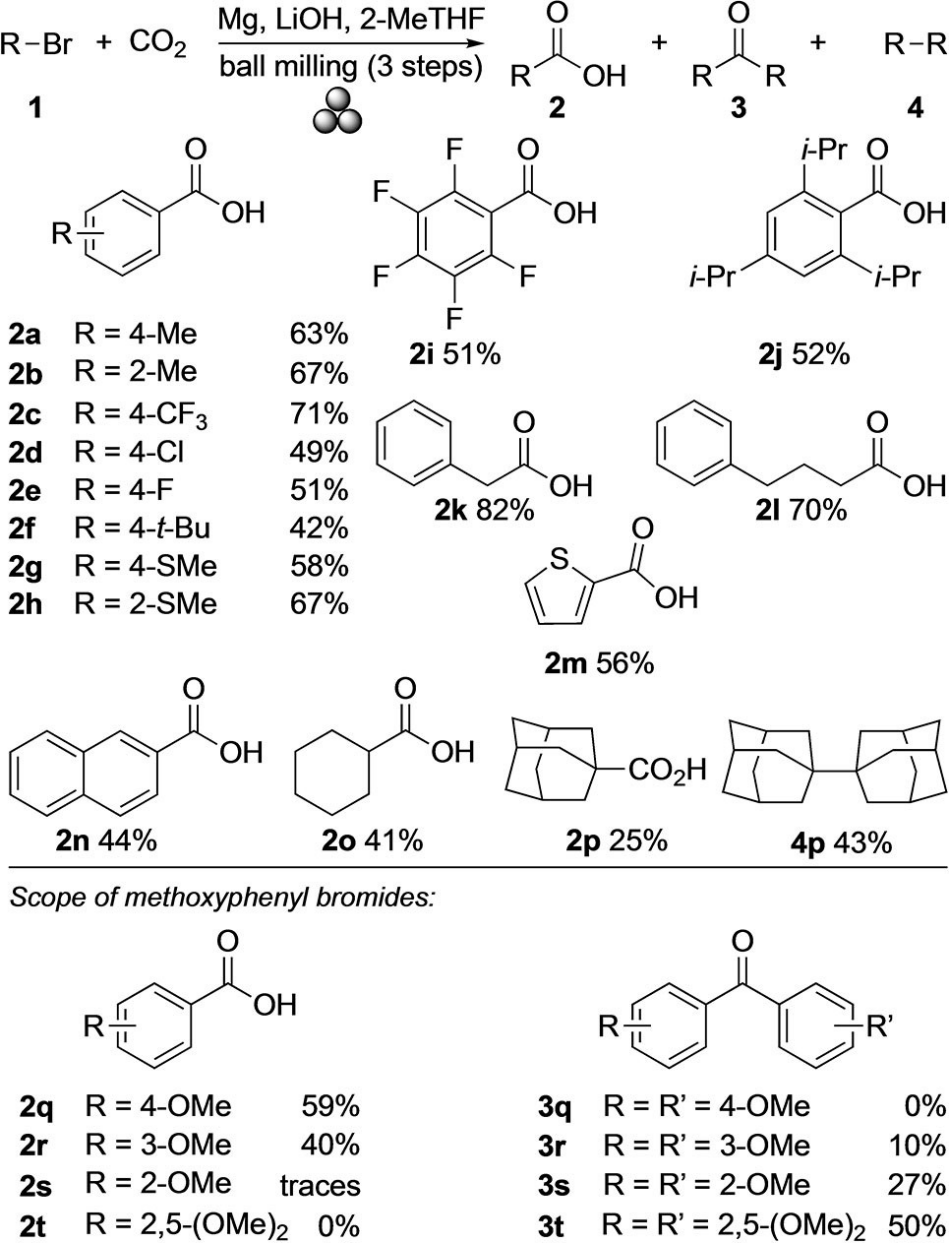
Scope of organobromides in mechanochemical Grignard reactions with CO_2_ (4.0 mmol scale). Reaction conditions (for steps I–III, see Table 1): Step I=Mg (2.5 equiv.) and LiOH (1.1 equiv.) in a ZrO_2_‐M milling vessel (20 mL) with gas inlet/outlet valves and 5 ZrO_2_‐M balls (Ø 10 mm) under Ar; step II=addition of 2‐MeTHF (2.0 equiv.) and **1** (4.0 mmol) under Ar; step III=CO_2_ (4 bar). The yields refer to product amounts obtained after column chromatography.

Like in solution, alkyl bromides performed better than arenes, yielding 82 % of phenylacetic acid (**2 k**) from benzyl bromide and 70 % of 4‐phenylbutyric acid (**2 l**) from the respective bromide. Bromocyclohexane (**1 o**) gave 41 % of cyclohexylcarboxylic acid (**2 o**). With 1‐bromoadamantane (**1 p**), the expected 1‐adamantylcarboxylic acid (**2 p**) was isolated in only 25 % yield, and the major product (43 %) was biadamantyl (**4 p**). This result suggested the formation of a rather stable adamantyl radical as intermediate, which homocoupled faster than it reacted with CO_2_.^[13a]^

An interesting reaction variation was observed in conversions of aryl bromides with strongly electron‐donating methoxy groups.

There, the precise substitution pattern was decisive. While 4‐methoxyphenyl bromide (**1 q**) led to a similar result (59 % yield of **2 q**) as 4‐tolyl bromide (**1 a**), moving the methoxy substituent to the *meta*‐position decreased the yield to 40 % for 3‐methoxybenzoic acid (**2 r**). Concomitant to **2 r**, 10 % of ketone **3 r** had been formed. With 2‐methoxyphenyl bromide (**1 s**), the expected acid was not obtained at all, but instead, 2,2′‐dimethoxybenzophenone (**3 s**) was isolated in 27 %. The trend of increased ketone formation with closer spatial proximity of the methoxy groups to the “site of reaction” was even more pronounced in the reaction with 2,5‐dimethoxybenzylbromide (**1 t**), which led to ketone **3 t** in 50 % yield. Overall, this ketone formation was remarkable as Grignard reactions of carboxylic acid derivatives are prone to lead directly to the corresponding tertiary alcohols.[^30^, ^31^, ^32^] Under our mechanochemical conditions, however, the initially formed magnesium carboxylate appears to be relatively stable. This salt allows for another Grignard reagent to be added, and the resulting dimagnesiate salt remains intact until its hydrolysis by aqueous workup to furnish the symmetric ketone.^[23]^

As demonstrated above, gaseous CO_2_ is a suitable reagent for mechanochemical Grignard reactions, but its handling requires specialized ball mill equipment. Thus, using a solid source of CO_2_ under those conditions appeared attractive. In this manner, the aforementioned technical challenges could be overcome, and mass transport issues arising from gas/solid reactions in the milling devices could be circumvented. The use of dry ice was excluded due to its property of attracting water through condensation hampering the desired organometallic reactivity.[^29^, ^33^] Inspired by the recent revival of sodium methyl carbonate (SMC) by Jessop, Snieckus and co‐workers,[^34^, ^35^] we decided to explore the potential of this very attractive source of solid, pre‐activated CO_2_ in mechanochemical Grignard reactions.

Starting from the previously optimised conditions for the use of gaseous CO_2_, magnesium was activated with lithium hydroxide at 600 rpm for 60 min (step I), and after the subsequent addition (step II) of 4‐tolyl bromide (**1 a**), 2‐MeTHF (2 equiv.) and SMC (1.5 equiv.), the resulting mixture was milled at 300 rpm for 45 min (Scheme 3). To our delight, aqueous workup and purification by column chromatography led to 4‐toluic acid (**2 a**) in 40 % yield. Compared to the method with gaseous CO_2_, this result was remarkable because first, by using SMC as electrophile the milling procedure was shortened by an entire step. Second, argon gas was not required, and third, the overall process time was significantly shorter.^[23]^ In attempts to improve the yield of **2 a** by varying the milling time in step II (60 min and 15 min), the product amount remained almost unchanged. Using more or less of SMC (2.0 equiv. and 1.0 equiv. versus 1.5 equiv. as before) reduced the yield of **2 a** (to 32 % and 31 %, respectively). Switching 2‐MeTHF to THF, adding a flake of iodine in step I, and varying the ball size proved ineffective as well (for details, see the Supporting Information).

**Scheme 3:**
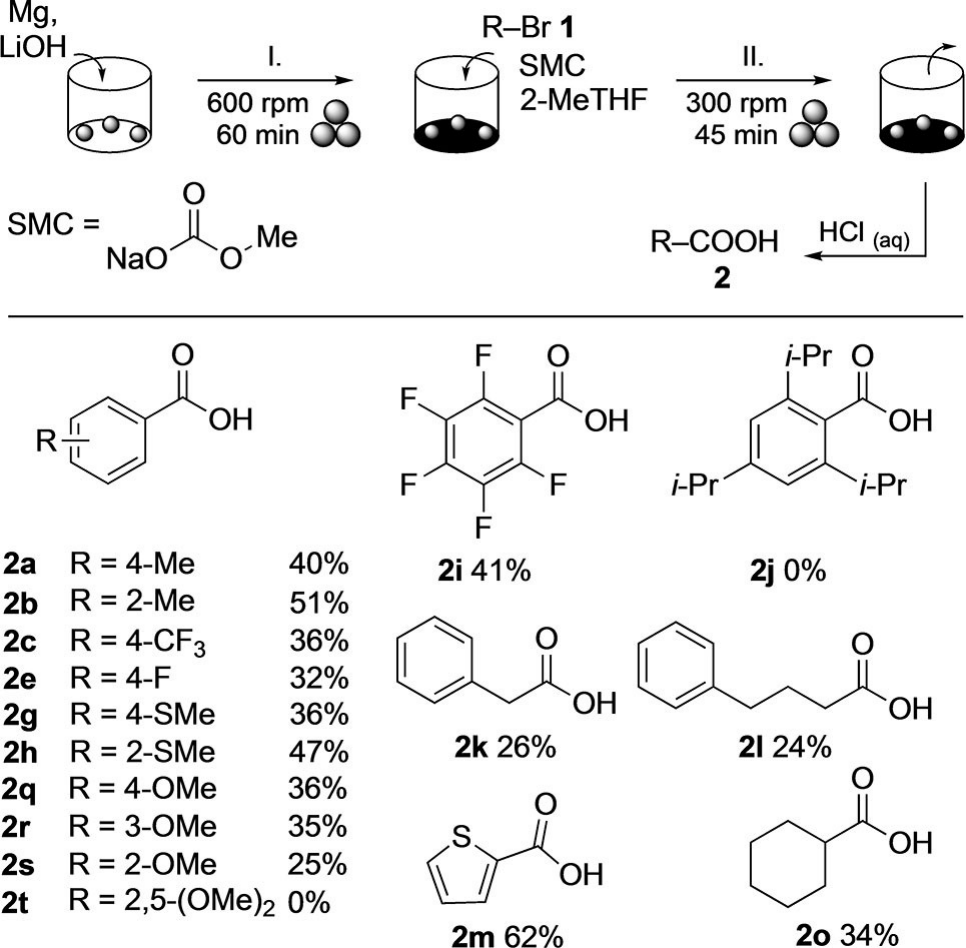
Scope of aryl and alkyl bromides in a mechanochemical Grignard reaction with SMC (1.0 mmol scale). Reaction conditions: Step I=Mg (2.5 equiv.) and LiOH (1.1 equiv.) in a ZrO_2_‐M milling vessel (12 mL) with 3 ZrO_2_‐M balls (Ø 9 mm); step II=addition of 2‐MeTHF (2.0 equiv.), SMC (1.5 equiv.), and **1** (1.0 mmol). The yields refer to product amounts obtained after column chromatography.

Examining the substate scope with SMC as reagent revealed a similar reactivity trend as observed with gaseous CO_2_. With the exception of derivatives **1 j** and **1 t** with multiple donating groups, all other aryl bromides afforded the corresponding acids albeit in mostly lower yields than with CO_2_. The best result was achieved in the formation of 2‐thiophenecarboxylic acid (**2 m**), which was obtained in 62 % yield. Fluoro and methoxy substituents were tolerated equally well independent of their position on the aryl group. Interestingly, 2‐methoxyphenyl bromide (**1 s**) was transformed into the respective acid (**2 s**) in 25 % yield compared to only traces in the reactions with CO_2_. Also with SMC, ketones **3** were detected, but generally, isolation led to only trace amounts of these side products. Although the SMC method was less efficient in terms of product yield, the approach appeared attractive considering the fact that no specialized gas equipment was needed.

In conclusion, we developed mechanochemical Grignard reactions with CO_2_ and SMC to prepare carboxylic acids from organobromides. In contrast to standard methods of this type, the protocol requires the presence of only 2 equivalents of an ethereal additive. Intriguingly, lithium hydroxide proved superior over lithium chloride, which is commonly applied in Grignard reactions as activating agent. With methoxy‐substituted aryl bromides, significant amounts of ketones are formed, contrasting observations made in solution‐based Grignard reactions. With SMC as C1 source, this reactivity is less pronounced.

## Conflict of interest

The authors declare no conflict of interest.

## Supporting information

As a service to our authors and readers, this journal provides supporting information supplied by the authors. Such materials are peer reviewed and may be re‐organized for online delivery, but are not copy‐edited or typeset. Technical support issues arising from supporting information (other than missing files) should be addressed to the authors.

Supporting Information

## Data Availability

The data that support the findings of this study are available in the Supporting Information of this article.
