# Supplementary material for: Mechanochemical Grignard Reactions with Gaseous CO2 and Sodium Methyl Carbonate
Source: Angew Chem Int Ed Engl. 2022 Jan 19;61(9):e202116514. doi: 10.1002/anie.202116514 (PMC9306648; doi:10.1002/anie.202116514)
Supplement: Supplementary file 1 — Supporting Information [file ANIE-61-0-s001.pdf]

Angewandte Chemie
Eine Zeitschrift der Gesellschaft Deutscher Chemiker

Supporting Information

Mechanochemical Grignard Reactions with Gaseous CO_2 and Sodium Methyl Carbonate

*V. S. Pfennig, R. C. Villella, J. Nikodemus, C. Bolm**

Table of contents

General information	S2
Experimental procedures	S3
Results and discussion	S7
Characterization of carboxylic acids, ketones, and other by-products	S21
References	S26
Author contributions	S26
^{1}H, $^{13}C\{^{1}H\}$, ^{19}F NMR spectra	S27

General information

All chemicals were obtained from commercial suppliers unless otherwise stated. Carbon dioxide (CO_2) was obtained from WESTFALEN with min. 99.8% purity. For thin-layer chromatography (TLC), TLC plates were used (silica gel 60 on aluminum plates with fluorescence indicator F254 from MERCK or pre-coated TLC sheets ALUGRAM® Xtra SIL G/UV_{254} from MACHEREY-NAGEL). TLC plates were analyzed qualitatively under UV light (λ = 254 nm) and/or by immersion into a CAM-stain {10 g $[(NH_4)_6(Mo_7O_{24})]\cdot 4\ H_2O$, 0.1 g $Ce(SO_4)_2$, 10 mL conc. H_2SO_4, 100 mL dist. H_2O} followed by heating the stained plates with a heat-gun until stains became visible. For column chromatography, silica gel 60 M (40-60 μm) from MACHEREY-NAGEL and Hyflo Super Cel®, diatomaceous earth from ALDRICH (referred to as Celite) were used and technical grade solvents were distilled prior to use. Tetrahydrofuran (THF) was purified with an MB-SPS-5 solvent purification system from MBRAUN, and 2-methyltatrahydrofuran (2-MeTHF) was obtained from J&K SCIENTIFIC (99%, SuperDry, stabilizer free, water $\leq$ 20 ppm).

Nuclear magnetic resonance (NMR) spectra were recorded either on a VARIAN VNMRS 400, VARIAN VNMRS 600, BRUKER Avance Neo 400 or BRUKER Avance Neo 600 spectrometer at 25 °C and were analyzed with the software MestReNova.[1] Carbon spectra were proton decoupled. Chemical shifts δ are reported in parts per million (ppm) and are referenced to the residual non-deuterated solvent signal of the respective solvent for ^{1}H NMR spectra and to the solvent signal in $^{13}C\{^{1}H\}$ NMR spectra ($CHCl_3$: ^{1}H NMR δ = 7.26 ppm; $CDCl_3$: $^{13}C\{^{1}H\}$ NMR δ = 77.16 ppm; acetone-d_5: ^{1}H NMR δ = 2.05 ppm; acetone-d_6: $^{13}C\{^{1}H\}$ NMR δ = 29.84 ppm).[2] Multiplicities of the signals are given using abbreviations and combinations thereof (s = singlet, d = doublet, t = triplet, q = quartet, p = pentet, sept. = septet, m = multiplet, br = broad signal). Coupling constants (*J*) are reported in Hertz (Hz).

Elemental analyses (CHN) were carried out with an ELEMENTAR VarioEL.

For mechanochemical reactions, a FRITSCH Pulverisette 7 premium line planetary micro mill, a FRITSCH Pulverisette 23 mini mill or a FRITSCH Pulverisette 7 classic line planetary micro mill was used. For each mill, magnesium oxide stabilized zirconium oxide (ZrO_2-M) milling vessels were used and paired with balls of the same material, if not stated otherwise.

Experimental procedures

Synthesis of sodium methyl carbonate (SMC) (modified literature procedure)[3]

A 250 mL two-neck pear shaped flask equipped with a septum and a Schlenk tap was evacuated and flushed with CO_2 thrice. Dry methanol (50 mL) and a solution of sodium methanolate in methanol (5.4 M, 5.6 mL, 30.2 mmol) were added with a syringe. The septum was exchanged for a glass gas inlet tube under counterflow of CO_2, and CO_2 was bubbled into the solution through the inlet tube for 2 h. After that, the precipitate was filtered over a Büchner funnel, rinsed with 50 mL of cold diethyl ether, and transferred into a Schlenk tube. The solid was then dried under reduced pressure at room temperature for 18 h. Sodium methyl carbonate was isolated as a colorless crystalline solid in 71% yield (2.106 g, 21.5 mmol).
^{1}H NMR (600 MHz, methanol-d$_4$) δ: 3.35 (s, 3H) ppm. **^{13}C{^{1}H} NMR** (150 MHz, methanol-d$_4$) δ: 161.4, 49.8 ppm.
CHN analysis: Calc. for $C_2H_3NaO_3$: C: 24.50, H: 3.08, N: 0.00; Found C: 24.62, H: 2.86, N: 0.00

General procedure 1 for the optimization of reaction conditions with CO_2

A 20 mL ZrO_2-M milling vessel with an atmosphere control lid with gas inlet/outlet valves was charged with 5 balls (10 mm diameter), magnesium turnings (243 mg, 10.0 mmol, 2.5 equiv.), and lithium hydroxide (105 mg, 4.4 mmol, 1.1 equiv.). The vessel was then closed, flushed with argon and ca. 0.3 bar argon were added, followed by milling at 600 rpm for 60 min. Afterwards, the argon atmosphere was relieved, the vessel was opened, 4-tolyl bromide (**1a**, 492 μL, 684 mg, 4.0 mmol, 1.0 equiv.), and 2-methyltetrahydrofuran (801 μL, 8.0 mmol, 2.0 equiv.) were added. The vessel was closed, flushed with argon and ca. 0.3 bar argon were added, followed by milling at 300 rpm for 2 x 30 min with 10 min break in between. When the milling finished, the vessel was flushed with CO_2 and 4 bar of CO_2 were added.[4] Then, the mixture was milled at 300 rpm for another 45 min before the pressure was relieved. The lid, main insulation ring, the balls and the milling container were washed with 10% aqueous HCl and ethyl acetate until all black and metallic residues were removed.* The two-phase mixture was transferred into a 250 mL separating funnel, the phases were separated, and the aqueous phase was extracted with 2 x 75 mL ethyl acetate. The combined organic layer was dried over $MgSO_4$, 1,3,5-trimethoxybenzene (168 mg, 1.0 mmol, 0.25 equiv.) was added as an internal standard and the solvent was evaporated under reduced pressure (160 mbar, 40 °C bath temperature) to dryness. A ^{1}H NMR spectrum was recorded, and the NMR yield was calculated based on the ratio of the integrals of 4-toluic acid **2a** (d at 8.02 ppm, C*H*) to 1,3,5-trimethoxybenzene (s at 6.09 ppm, C*H*). In some cases, the crude product was then loaded onto Celite and purified by column chromatography over ca. 37 g of silica gel, eluting with a mixture of diethyl ether/*n*-pentane with traces of acetic acid (ca. 10 drops per 500 mL of solvent mixture).
**Note: Before the lid could come into contact with HCl or ethyl acetate, the valves and their insulation rings were removed, taken apart and cleaned thoroughly with ethanol (technical grade) and a lint-free paper towel. After the removal of all residues from the milling equipment, the lid, main insulation ring, balls and milling vessel were cleaned with water, little scouring agent and acetone, and left to dry at ambient atmosphere.*

General procedure 2 for the optimization of reaction conditions with sodium methyl carbonate in a mixer mill

A 10 mL ZrO_2-M milling vessel was charged with one ball (10 mm diameter), magnesium turnings (61 mg, 2.5 mmol, 2.5 equiv.), and lithium hydroxide (26 mg, 1.1 mmol, 1.1 equiv.). The vessel was then closed, sealed with insulation tape at the seam, and the mixture was milled at 35 Hz for 60 min. Afterwards, the vessel was opened, sodium methyl carbonate (147 mg, 1.5 mmol, 1.5 equiv.), 4-tolyl bromide (**1a**, 123 μL, 171 mg, 1.0 mmol, 1.0 equiv.), and 2-methyltetrahydrofuran (200 μL, 2.0 mmol, 2.0 equiv.) were added. The vessel was closed again and re-sealed with insulation tape at the seam, followed by milling at 20 Hz for 45 min. When the milling finished, the lid, ball and

bottom part of the milling vessel were washed with 10% aqueous HCl and ethyl acetate until all black and metallic residues were removed.* The two-phase mixture was transferred into a 100 mL separating funnel, the phases were separated, and the aqueous phase was extracted with 3 x 30 mL ethyl acetate. The combined organic layer was dried over $MgSO_4$, 1,3,5-trimethoxybenzene (42 mg, 0.25 mmol, 0.25 equiv.) was added as an internal standard and the solvent was evaporated under reduced pressure (160 mbar, 40 °C bath temperature) to dryness. A ^1H NMR spectrum was recorded, and the NMR yield was calculated based on the ratio of the integrals of **2a** (d at 8.02 ppm, C*H*) to 1,3,5-trimethoxybenzene (s at 6.09 ppm, C*H*). In some cases, the crude product was then loaded onto Celite and purified by column chromatography over ca. 23 g of silica gel, eluting with a mixture of diethyl ether/*n*-pentane with traces of acetic acid (ca. 10 drops per 500 mL of solvent mixture).

**Note: The lid, balls and bottom part were cleaned with water, scouring agent and acetone, and left to dry at ambient atmosphere.*

General procedure 3 for the optimization of reaction conditions with sodium methyl carbonate in a planetary mill

A 12 mL ZrO_2-M milling vessel was charged with 3 balls (9 mm diameter), magnesium turnings (61 mg, 2.5 mmol, 2.5 equiv.), and lithium hydroxide (26 mg, 1.1 mmol, 1.1 equiv.). The vessel was then closed, sealed with insulation tape at the seam, followed by milling at 600 rpm for 60 min. Afterwards, the vessel was opened, sodium methyl carbonate (147 mg, 1.5 mmol, 1.5 equiv.), 4-tolyl bromide (**1a**, 123 μL, 171 mg, 1.0 mmol, 1.0 equiv.), and 2-methyltetrahydrofuran (200 μL, 2.0 mmol, 2.0 equiv.) were added. The vessel was closed and re-sealed with insulation tape at the seam, followed by milling at 300 rpm for 45 min. When the milling finished, the lid, milling container and balls were washed with 10% aqueous HCl and ethyl acetate until all black and metallic residues were removed.* The two-phase reaction mixture was transferred into a 100 mL separating funnel, the phases were separated, and the aqueous phase was extracted with 3 x 30 mL ethyl acetate. The combined organic layer was dried over $MgSO_4$, and the solvent was evaporated under reduced pressure (160 mbar, 40 °C bath temperature) to dryness. A ^1H NMR spectrum was recorded, and the crude product was then loaded onto Celite and purified by column chromatography over ca. 23 g of silica gel, eluting with a mixture of diethyl ether/*n*-pentane with traces of acetic acid (ca. 10 drops per 500 mL of solvent mixture).

**Note: The lid, insulation ring, balls and milling vessel were cleaned with water, scouring agent and acetone, and left to dry at ambient atmosphere.*

Description of general procedure 1 in pictures

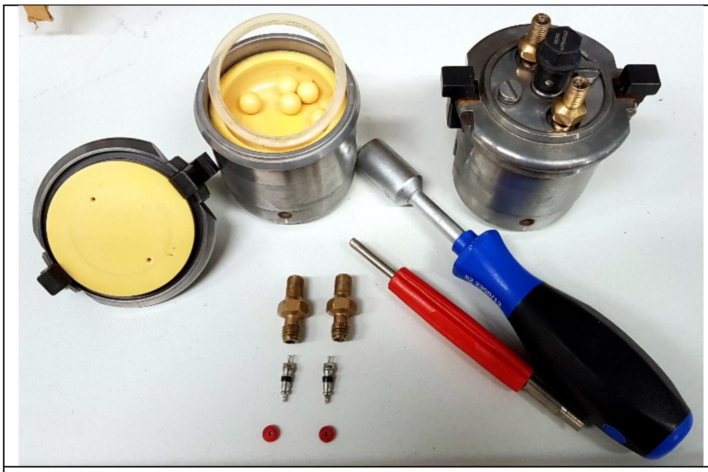

1. Assembly of the milling vessels. Left: milling vessel with open grinding chamber, 5 ZrO_2-M balls (10 mm diameter), main insulation ring and lid; valves with inner valves and valve insulation. Right: assembled vessel with gas valves, required tools in the bottom.

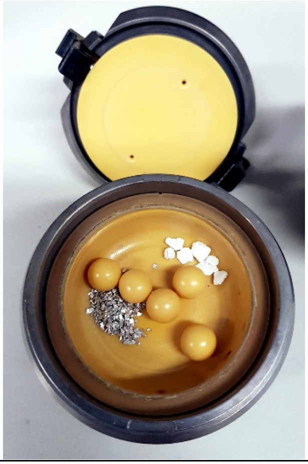

2. Open grinding chamber with balls and main insulation ring after weighing magnesium turnings and lithium hydroxide, lid in the background.

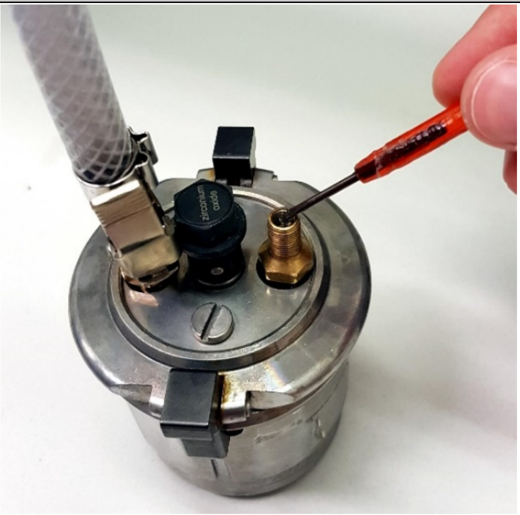

3. Flushing the milling vessel with Argon gas before milling magnesium turnings with lithium hydroxide.

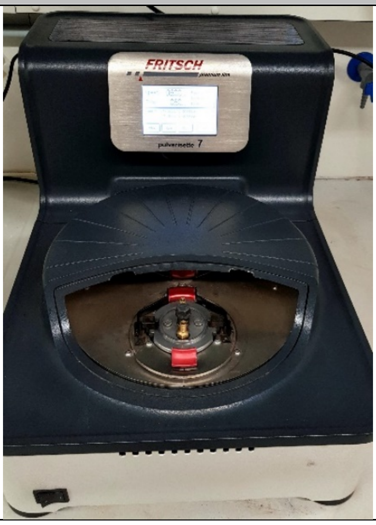

4. Open FRITSCH Pulverisette 7 premium line planetary micro mill with the milling vessel and reaction parameters in the device panel before step I.

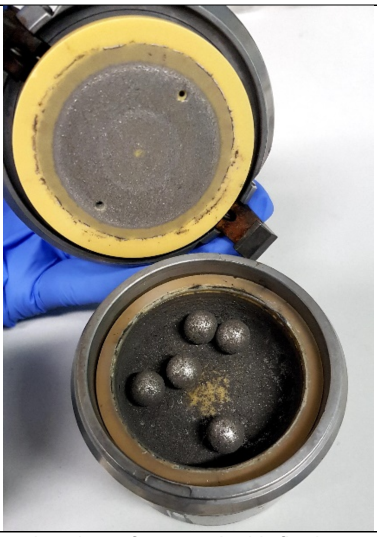

5. Open grinding chamber after step I with finely ground magnesium and lithium hydroxide.

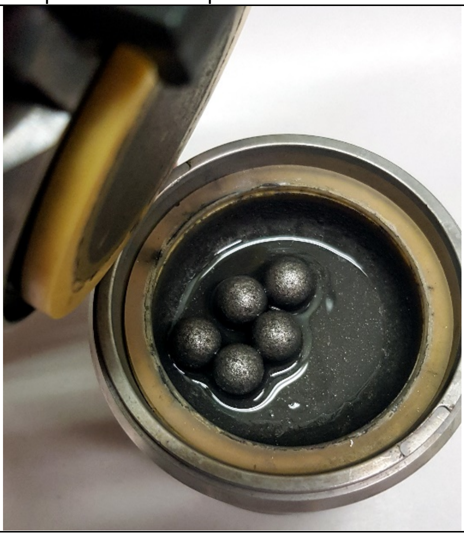

6. Grinding chamber after addition of organobromide and 2-MeTHF; followed by flushing with Argon (3.) and milling (4.).

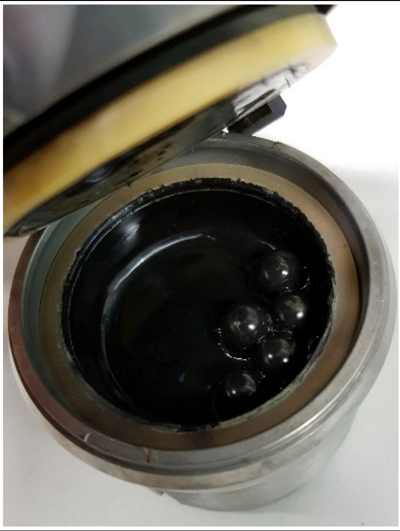

7. Reaction mixture prior to addition of CO_2; here, the lid is flushed to remove residue from the holes.[4] Followed by flushing with CO_2 (like 3.) and milling (4.).

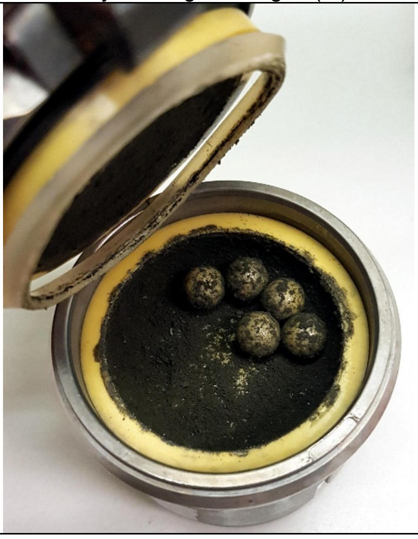

8. Powdery reaction mixture after grinding under CO_2 atmosphere and prior to aqueous workup.

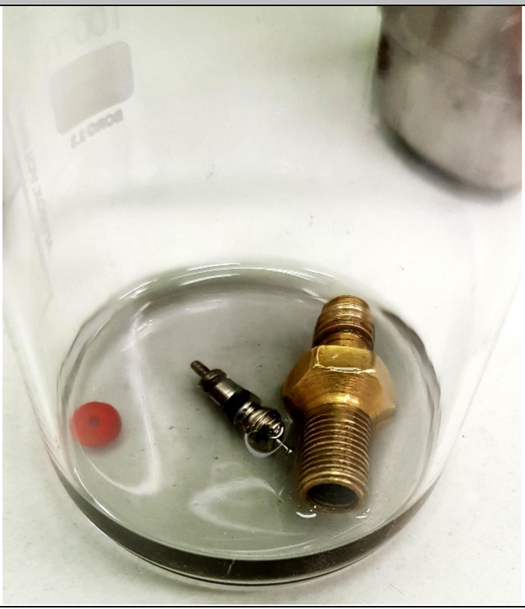	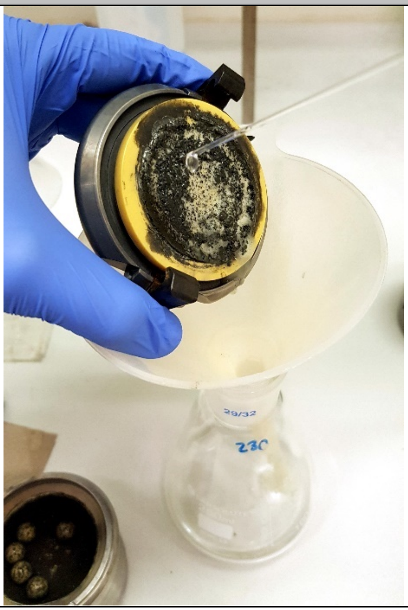
9. Valve, inner valve, and valve insulation in ethanol during the cleaning process.	10. Residue on the lid reacting with 10% aqueous HCl during the workup procedure.
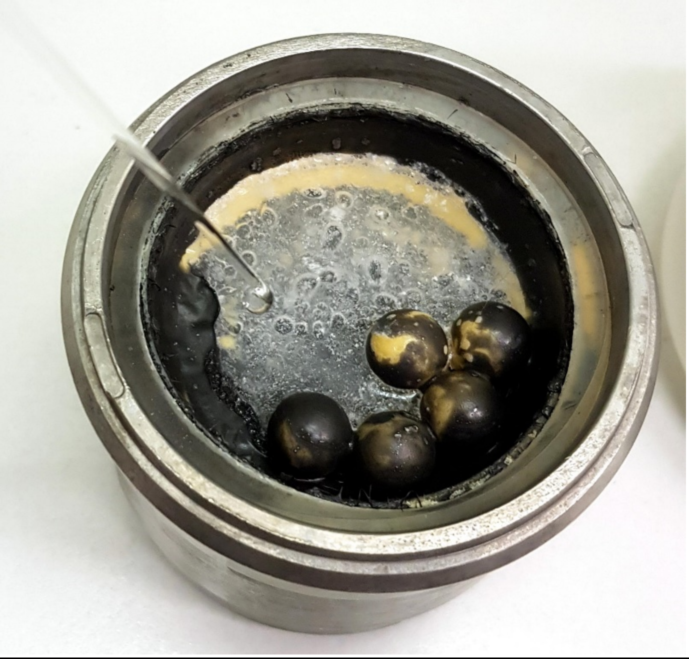	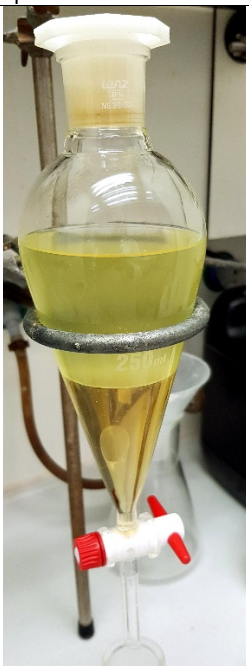
11. Reaction mixture in the milling vessel reacting with 10% aqueous HCl during the workup procedure.	12. Two-phase reaction mixture before phase separation.

Results and discussion

Optimization of the milling conditions with CO_2

To begin the investigation, magnesium and starting material **1a** were milled together under a CO_2 atmosphere with equimolar amounts of magnesium turnings (Table S1, entry 1) and two equivalents of magnesium (entry 2). As no product was detected, it was decided to make use of the increased and fresh surface areas as one of the major advantages in mechanochemistry, and milling of the magnesium before adding the substrate and CO_2 yielded 4% of the acid **2a** after isolation (entry 3). In another project, the wear and tear of the milling material was closely investigated, and it was found that milling less than 190 mg of total material severely wears the five balls in this specific setup, meaning they lose mass. Hence, by separating the activation of magnesium, the minimum scale had to be increased to 4 mmol of **1a** so that 2.0 equiv. magnesium turnings (194 mg) were used in Step **I**.

An approach to lengthen the reaction time did not help to increase the yield (entry 4); yet separating the step of the formation of the Grignard reagent from the reaction with the electrophile raised the yield to 17% (entry 5). The duration of the reaction, the aqueous workup and the evaporation of ethyl acetate took up an entire working day with such reaction times and quantities *and* required an additional working day to isolate the compounds and determine the yield. Because of this, it was decided to test the determination of the yield by ^{1}H NMR spectroscopy using 1,3,5-trimethoxybenzene as an internal standard. Entries 5 and 8 prove that the yields obtained in each method are comparable. Furthermore, the milling speed was reduced for steps **II** and **III** to minimize the energy input, the related wear and tear of the milling balls and the formation of side products (entry 6).

The beneficiary effects of adding THF as Lewis base and lithium chloride as activation additive as well as the reasons behind this are discussed in the main article. Nonetheless, in the entries 7 to 9, the reaction time for the formation of the Grignard reagent in step **II** was shortened consecutively, and yet the yield of the acid **2a** increased. The reaction time with CO_2 in step **III** was reduced to 45 min, further raising the yield to 59% after isolation (entry 10). More attempts to reduce the overall reaction time brought lower yields of the acid **2a** (entries 11-18). Interestingly, the positive effect of separating the respective steps in the beginning of the optimization of milling conditions became less important at these short reaction times: two entry pairs (11 and 12, 13 and 14) show that the separation has no significant effect on the yield of the acid **2a**. Therefore, the general trend indicates that reaction times shorter than the optimum 60 min for the Grignard reagent formation and 45 min for the reaction with the electrophile CO_2 decrease the yield.

Still, a procedure with fewer steps or even all reagents added at the same time was deemed advantageous. Thus, it was tested to leave out step **I** (activation of magnesium), which led to recovered 50% yield according to the ^{1}H NMR (entry 15). However, leaving out step **I** and **II**, thus combining all reagents and reactants and milling them at once, reduced the NMR yield to 19%. It was concluded that the procedure performed best if the three steps were carried out separately and for the amounts of time indicated in the equation above table S1.

Lastly, after discovering the superior reactivity of lithium hydroxide in comparison to lithium chloride, the milling parameters were re-evaluated and lithium hydroxide was revealed to cause 50% NMR yield of the acid **2a** to form after only 30 min overall milling time (15 min step **II**, 15 min step **III**).

Table S1. Effect of changing the milling conditions in a mechanochemical Grignard reaction of **1a** with CO_2 in a planetary ball mill.

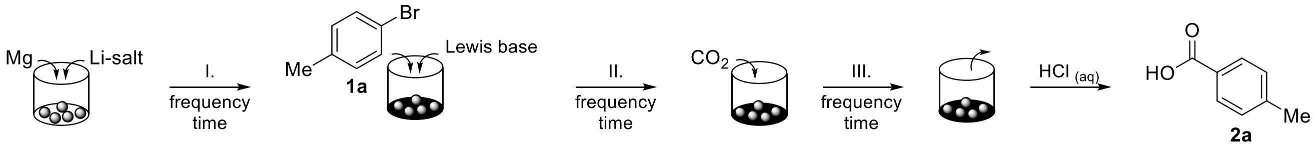

Entry[a]	Mg [equiv.]	**1a** [mmol]	Step I: Li-salt	Step I:[b] Mg activation	Step II: Lewis base	Step II:[b] Grignard formation	Step III:[c] Addition of CO_2	NMR Yield of **2a** [%][d]
1	1	2	-	>	-	>	600 rpm, 90 min	0
2	2	2	-	>	-	>	600 rpm, 90 min	0
3	2	4	-	600 rpm, 90 min	-	>	600 rpm, 180 min	n.d. (4)
4	2	4	-	600 rpm, 30 min	-	>	600 rpm, 280 min	3
5	2	4	-	600 rpm, 30 min	-	600 rpm, 180 min	600 rpm, 90 min	18 (17)
6	2	4	-	600 rpm, 30 min	-	300 rpm, 120 min	300 rpm, 60 min	9
7	2	4	-	600 rpm, 60 min	2.0 equiv. THF	300 rpm, 240 min	300 rpm, 60 min	35 (25)
8	2.5	4	1.25 equiv. LiCl	600 rpm, 60 min	2.0 equiv. THF	300 rpm, 180 min	300 rpm, 60 min	35 (31)
9	2.5	4	1.0 equiv. LiCl	600 rpm, 60 min	0.9 equiv. THF	300 rpm, 120 min	300 rpm, 60 min	55 (42)
10	2.5	4	1.0 equiv. LiCl	600 rpm, 60 min	2.0 equiv. THF	300 rpm, 60 min	300 rpm, 45 min	62 (59)
11	2.5	4	1.0 equiv. LiCl	600 rpm, 60 min	2.0 equiv. THF	300 rpm, 15 min	300 rpm, 15 min	43
12	2.5	4	1.0 equiv. LiCl	600 rpm, 60 min	2.0 equiv. THF	>	300 rpm, 30 min	40
13	2.5	4	1.0 equiv. LiCl	600 rpm, 60 min	2.0 equiv. THF	300 rpm, 5 min	300 rpm, 5 min	25
14	2.5	4	1.0 equiv. LiCl	600 rpm, 60 min	2.0 equiv. THF	>	300 rpm, 10 min	26
15	2.5	4	1.0 equiv. LiCl	>	2.0 equiv. THF	300 rpm, 30 min	300 rpm, 30 min	50
16	2.5	4	1.0 equiv. LiCl	>	2.0 equiv. THF	>	300 rpm, 60 min	19
17	2.5	4	1.1 equiv. LiOH	600 rpm, 60 min	2.0 equiv. THF	300 rpm, 15 min	300 rpm, 15 min	50

[a] Reaction conducted in a 20 mL ZrO_2-M milling vessel with gas inlet/outlet valves and 5 ZrO_2-M balls of 10 mm diameter. [b] The milling steps were conducted with the milling vessel under argon atmosphere; the manipulation and weighing were carried out under ambient atmosphere. “>“ signifies that this step was combined with the next step under the conditions written there. [c] Step III was conducted with a CO_2 atmosphere in the milling vessel. [d] The NMR yield was determined as described in General Procedure 1, with yields after isolation in brackets in case they were determined.

Optimization of the amount of magnesium

Previously described procedures for mechanochemical Grignard reactions by both Harrowfield *et al.* and Speight and Hanusa require 4 to 8 equiv. of magnesium.[5,6] On the opposite, the procedure developed here gave fair yields of the acid **2a** with only 2.5 equiv. of magnesium. Nonetheless, the impact of excess and stoichiometric amounts of magnesium was investigated (Table S2). A remarkably high NMR yield was detected with 1.0 equiv. of magnesium after only 15 min of milling (55%, entry 1). The trend in the other entries, however, showed that until 4.0 equiv., the yield increased with more magnesium and at longer reaction times. Several reasons justified the use of 2.5 equiv. of magnesium instead of more: First, the increase in yield after isolation from 2.5 to 4.0 equiv. consisted of only 4% (entries 4-6). Second, Harrowfield *et al.* had shown that an excess amount of magnesium triggered McMurry reactions, although in this case a different electrophile was used. And third, with more magnesium, the workup became more difficult and time-consuming. For example, with 8.0 equiv. of magnesium, a yield of only 17% yield was obtained, indicating problematically low concentrations of the aryl bromide **1a** in comparison to the magnesium. In any case, the use of 2.5 equiv. of magnesium was considered an improvement over previously reported procedures regarding efficiency and yield.

Table S2. Effect of changing the amount of magnesium in a mechanochemical Grignard reaction of **1a** with CO_2 in a planetary ball mill.

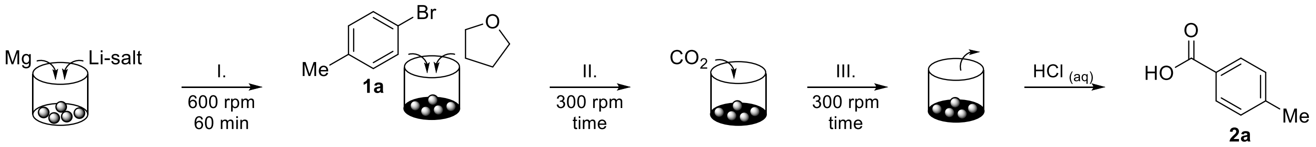

Entry[a]	Mg [equiv.]	Step I:[b] Additive	Step II:[c] Grignard formation	Step III:[d] Addition of CO_2	NMR Yield of **2a** [%][e]
1	1.0	1.0 equiv. LiCl	15 min	15 min	55
2	2.0	1.0 equiv. LiCl	15 min	15 min	35
3	2.5	1.1 equiv. LiOH	15 min	15 min	43
4	2.5	1.1 equiv. LiOH	60 min	45 min	70 (64)
5	3.0	1.1 equiv. LiOH	60 min	45 min	81 (65)
6	4.0	1.1 equiv. LiOH	60 min	45 min	80 (68)
7	8.0	1.1 equiv. LiOH	60 min	45 min	16 (17)

[a] Reaction conducted in a 20 mL ZrO_2-M milling vessel with gas inlet/outlet valves and 5 ZrO_2-M balls of 10 mm diameter. [b] The indicated amount of magnesium was milled with the indicated additive at 600 rpm for 60 min with the milling vessel under argon atmosphere in step I. [c] After the addition of 4 mmol of **1a** and 2.0 equiv. of THF, the mixture was milled for the indicated amount of time at 300 rpm under argon atmosphere in step II; the manipulation and weighing were carried out under ambient atmosphere. [d] Step III was conducted with a CO_2 atmosphere in the milling vessel at 300 rpm for the indicated amount of time. [e] The NMR yield was determined as described in General Procedure 1, with yields after isolation in brackets in case they were determined.

Optimization of the lithium salt and equivalents

Since the optimization of milling conditions alone did not increase the yield beyond traces of 4-toluic acid (**2a**), several additives to enhance the speed of the reaction were tried (Table S3). The addition of one flake of iodine is normally used to overcome the limits of passivated magnesium turnings; yet in our setup, it hardly had an impact on the yield. In the main article, the impact of using a lithium salt additive is described. First reactions using 1.0 and 1.25 equiv. of lithium chloride on the dry reaction mixture did not significantly enhance the yield (2% and 5%, entries 2 and 3); however, in combination with THF, the yield increased notably. Screening different amounts of lithium chloride revealed that 1.0 or 1.1 equiv. of lithium chloride enhanced the reaction best with 59% and 60% yield, respectively (compare entries 4-9).

As Knochel and coworkers had investigated different lithium salts in their works towards turbo Grignard reagents,[7] it was tested whether the salts performed similarly under mechanochemical conditions. Among the other halogenides, the fluoride gave the best result with 18% NMR yield (entry 10). The bromide gave no product (entry 11), while the iodide led to 5% NMR yield (entry 12). Lithium acetate, carbonate and tetrafluoroborate also inhibited the formation of the acid **2a** (entries 13-15). In comparison with Knochel's work, our approach was thus found to be relatively demanding towards the activation additive. Lithium phosphate helped to generate 15% NMR yield of the acid **2a** (entry 16), but lithium hydroxide gave the most remarkable result with 80% yield after isolation (entry 17). To the best of our knowledge, lithium hydroxide has not been investigated for accelerating magnesium insertion reactions before.

With this unexpected replacement for lithium chloride, the best amount of lithium hydroxide to be added to the reaction mixture was re-investigated. Neither the use of more nor less salt than the initially tested 1.1 equiv. improved the yield (compare entries 17-21). The yield did not decrease below 36% even with 5.0 equiv. of lithium hydroxide. Therefore, the investigation was continued using 1.1 equiv. of lithium hydroxide. In addition, lithium chloride was occasionally tested again.

Table S3. Effect of changing the lithium salt for activating a mechanochemical Grignard reaction of **1a** with CO_2 in a planetary ball mill.

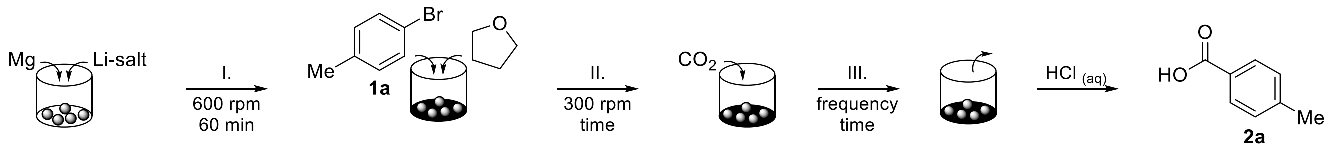

Entry[a]	Mg [equiv.]	Step I:[b] Additive	Step II: Additive	Step II:[c] Grignard formation	Step III:[d] Addition of CO_2	NMR Yield of **2a** [%][e]
1[f]	2	1 mol-% I_2	-	>	600 rpm, 180 min	n.d. (3)
2	2.5	1.25 equiv. LiCl	-	180 min	300 rpm, 60 min	2
3	2.5	1.0 equiv. LiCl	-	120 min	300 rpm, 60 min	5
4	2.5	0.25 equiv. LiCl	2.0 equiv. THF	60 min	300 rpm, 45 min	15
5	2.5	0.5 equiv. LiCl	2.0 equiv. THF	60 min	300 rpm, 45 min	37
6	2.5	1.0 equiv. LiCl	2.0 equiv. THF	60 min	300 rpm, 45 min	62 (59)
7	2.5	1.1 equiv. LiCl	2.0 equiv. THF	60 min	300 rpm, 45 min	58 (60)
8	2.5	1.25 equiv. LiCl	2.0 equiv. THF	60 min	300 rpm, 45 min	37
9	1.0	1.5 equiv. LiCl	2.0 equiv. THF	20 min	300 rpm, 20 min	19
10	2.5	1.1 equiv. LiF	2.0 equiv. THF	60 min	300 rpm, 45 min	18
11	2.5	1.1 equiv. LiBr	2.0 equiv. THF	60 min	300 rpm, 45 min	0
12	2.5	1.1 equiv. LiI	2.0 equiv. THF	60 min	300 rpm, 45 min	5
13	2.5	1.1 equiv. LiOAc	2.0 equiv. THF	60 min	300 rpm, 45 min	0
14	2.5	0.55 equiv. Li_2CO_3	2.0 equiv. THF	60 min	300 rpm, 45 min	0
15	2.5	1.1 equiv. $LiBF_4$	2.0 equiv. THF	60 min	300 rpm, 45 min	0
16	2.5	0.37 equiv. Li_3PO_4	2.0 equiv. THF	60 min	300 rpm, 45 min	15
17	2.5	1.1 equiv. LiOH	2.0 equiv. THF	60 min	300 rpm, 45 min	75 (80)
18	2.5	0.9 equiv. LiOH	2.0 equiv. THF	60 min	300 rpm, 45 min	63
19	2.5	1.0 equiv. LiOH	2.0 equiv. THF	60 min	300 rpm, 45 min	55
20	2.5	2.5 equiv. LiOH	2.0 equiv. THF	60 min	300 rpm, 45 min	50 (38)
21	2.5	5.0 equiv. LiOH	2.0 equiv. THF	60 min	300 rpm, 45 min	46 (36)

[a] Reaction conducted in a 20 mL ZrO_2-M milling vessel with gas inlet/outlet valves and 5 ZrO_2-M balls of 10 mm diameter. [b] The indicated amount of magnesium was milled with the indicated additive at 600 rpm for 60 min with the milling vessel under argon atmosphere in step I. [c] After the addition of 4 mmol of **1a** and the indicated amount of THF, the mixture was milled under argon atmosphere at 300 rpm for the indicated amount of time in step II; the manipulation and weighing were carried out under ambient atmosphere. “>“ signifies that this step was combined with the next step under the conditions written there. [d] Step III was conducted with a CO_2 atmosphere in the milling vessel at under the indicated conditions. [e] The NMR yield was determined as described in General Procedure 1, with yields after isolation in brackets in case they were determined. [f] The activation step I was milled for 90 min instead of 60 min.

Optimization of the Lewis base and equivalents

Although the first encounter of most chemistry students with Grignard reactions may not necessarily involve working under Schlenk conditions, using dry ethereal solvents has so far been irreplaceable as explained in the main article. During the optimization of milling conditions, the maximum yield of **2a** without solvent was 17% (table S1, entry 5). When small amounts of THF (0.67 equiv.) were added for liquid assisted grinding (LAG), the yield increased to 25% of **2a** in the NMR (Table S4, entry 1). When the amount of THF was increased to 2.0 equiv. to satisfy the Schlenk equilibrium equation, the yield rose to 35%, even without lithium chloride (entries 2 and 4). Diethyl ether performed slightly worse (30%, entry 3), and thus the amount of THF was optimized. While 0.9 equiv. gave almost

the same result as 2.0 equiv., the longer reaction time may account for this oddity since 1.0 equiv. only gave 18% NMR yield at the standard reaction times (entries 5, 7, 8).

Table S4. Effect of changing the Lewis basic additive in a mechanochemical Grignard reaction of **1a** with CO_2 in a planetary ball mill.

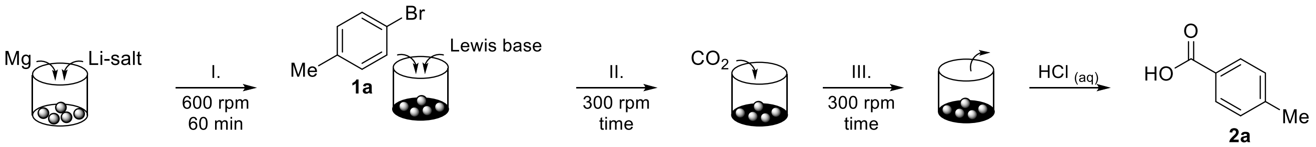

Entry[a]	Mg [equiv.]	Step I:[b] Additive	Step II: Lewis basic additive	Step II:[c] Grignard formation	Step III:[d] Addition of CO_2	NMR Yield of **2a** [%][e]
1	2	-	0.67 equiv. THF (η = 0.25)	120 min	60 min	25
2	2	-	2.0 equiv. THF (η = 0.75)	240 min	60 min	35
3	2	-	2.0 equiv. Et_2O (η = 0.95)	240 min	60 min	30
4	2.5	1.25 equiv. LiCl	2.0 equiv. THF (η = 0.58)	180 min	60 min	35 (31)
5	2.5	1.0 equiv. LiCl	0.9 equiv. THF (η = 0.27)	120 min	60 min	55
6	2.5	1.0 equiv. LiCl	-	120 min	60 min	5
7	2.5	1.0 equiv. LiCl	1.0 equiv. THF (η = 0.30)	60 min	45 min	18
8	2.5	1.0 equiv. LiCl	2.0 equiv. THF (η = 0.60)	60 min	45 min	62 (59)
9	2.5	1.0 equiv. LiCl	2.0 equiv. PEG-4000	60 min	45 min	0[f]
10	2.5	1.1 equiv. LiCl	2.0 equiv. γ-Valerolactone (η = 0.69)	60 min	45 min	0
11	2.5	1.1 equiv. LiOH	2.0 equiv. γ-Butyrolactone (η = 0.60)	60 min	45 min	0[f]
12	2.5	1.1 equiv. LiOH	2.0 equiv. Cyrene™ (η = 0.79)	60 min	45 min	0[f,g]
13	2.5	1.1 equiv. LiOH	2.0 equiv. 2,5-$(MeO)_2$-THF (η = 1.01)	60 min	45 min	0[f]
14	2.5	1.1 equiv. LiOH	2.0 equiv. $CH_3OCH_2OCH_3$ (η = 0.69)	60 min	45 min	10
15	2.5	1.1 equiv. LiOH	2.0 equiv. 1,3-Dioxolane (η = 0.54)	60 min	45 min	0
16	2.5	1.1 equiv. LiOH	2.0 equiv. Diglyme (η = 1.10)	60 min	45 min	0
17	2.5	1.1 equiv. LiOH	2.0 equiv. 2-MeTHF (η = 0.78)	60 min	45 min	n.d. (63)
18	2.5	1.1 equiv. LiOH	2.0 equiv. Et_2O (η = 0.81)	60 min	45 min	34
19	2.5	1.1 equiv. LiOH	2.0 equiv. Ph_2O (η = 1.23)	60 min	45 min	0
20	2.5	1.1 equiv. LiOH	2.0 equiv. DBU (η = 1.16)	60 min	45 min	1
21	2.5	1.1 equiv. LiOH	2.0 equiv. TMEDA (η = 1.16)	60 min	45 min	0
22	2.5	1.1 equiv. LiOH	2.0 equiv. DIPEA (η = 1.35)	60 min	45 min	1
23	2.5	1.1 equiv. LiOH	2.0 equiv. DABCO	60 min	45 min	0[f]
24	2.5	1.1 equiv. LiOH	2.0 equiv. THF, 2.0 equiv. DABCO	60 min	45 min	4
25	2.5	1.1 equiv. LiOH	2.0 equiv. THF, 2.0 equiv. DBU	60 min	45 min	15
26	2.5	1.1 equiv. LiOH	2.0 equiv. THF, 1.0 equiv. DBU	60 min	45 min	9
27	2.5	1.1 equiv. LiOH	2.0 equiv. THF, 0.5 equiv. DBU	60 min	45 min	23
28	2.5	1.1 equiv. LiOH	5.0 equiv. THF (η = 1.59)	60 min	45 min	65 (61)

PEG = Polyethylene glycol; DBU = 1,8-Diazabicyclo(5.4.0)undec-7-ene; TMEDA = Tetramethylethylenediamine; DIPEA = *N*,*N*-Diisopropylethylamine (Hünig's base); DABCO = 1,4-diazabicyclo[2.2.2]octane. [a] Reaction conducted in a 20 mL ZrO_2-M milling vessel with gas inlet/outlet valves and 5 ZrO_2-M balls of 10 mm

diameter. [b] The indicated amount of magnesium was milled with the indicated additive at 600 rpm for 60 min with the milling vessel under argon atmosphere in step I. [c] After the addition of 4 mmol of **1a** and the indicated Lewis Base, the mixture was milled under argon atmosphere at 300 rpm for the indicated amount of time in step II; the manipulation and weighing were carried out under ambient atmosphere. [d] Step III was conducted with a CO_2 atmosphere in the milling vessel at 300 rpm for the indicated amount of time. [e] The NMR yield was determined as described in General Procedure 1, with yields after isolation in brackets in case they were determined. [f] Starting material was recovered. [g] Decomposition of Cyrene™ was observed.

A control experiment with lithium chloride and without any solvent confirmed the increases in reaction rate and conversion caused by THF (entry 6). The screening of several additives with ether functionalities under the optimum milling conditions revealed that, besides THF and diethyl ether, only dimethoxymethane and 2-MeTHF led to the conversion towards the acid **2a** (entries 9-19). Given that the reaction was entirely inhibited with diphenyl ether and with diglyme, liquid assisted grinding with a “Grignard-resistant” solvent alone was shown to not suffice to trigger the magnesium insertion reaction or the formation of the acid **2a**. Equally, the failure of PEG as a solid ether proved that the ether functionality alone did not stabilize the Schlenk equilibrium enough in our ball milling approach. The combination of the liquid state and the ether functionality with the right degree of Lewis basicity of the additive was therefore decisive for the yield.
Nonetheless, the next screening focused on nitrogen-containing Lewis bases to fine-tune the interaction between the Grignard reagents and their environment. Additionally, 1,8-diazabicyclo(5.4.0)undec-7-ene (DBU) known to form an adduct with CO_2 in THF,[8] probably improved any potential phase transfer issues from gaseous to solid in our setup. However, only trace amounts of **2a** were detected after workup when DBU or Hünig’s base were used (entries 20-25). Even in combination with THF, the addition of DBU reduced the yield to 23% (entries 25-27). Furthermore, the plain volume of reagents inside the milling vessel can complicate the flushing procedures and cause the loss of material.[4]
Lastly, it was confirmed that no more than 2.0 equiv. of solvent were required and useful, since the yield did not improve beyond 61% with 5.0 equiv. of THF (entry 28). Although the volume of solvent per milligram of reaction mixture could still be considered LAG (η = 1.59 μL/mg), this surplus also promoted side reactions and made the workup procedure and purification rather difficult. As outlined in the main article, it was decided to use 2-MeTHF to investigate the substrate tolerance of the protocol.

Testing other aryl halogenides as starting material

Here, 4-tolyl bromide (**1a**) was used for all optimization reactions due to its straightforward detection in ^{1}H NMR spectroscopy. A pressing question may arise as to why the bromide was chosen for these reactions although textbook chemistry suggests aryl iodides to be more reactive. Under optimum conditions, we compared the reactivity of the other aryl halides (Table S5). In contrast to the work by Speight and Hanusa,[9] 4-tolyl fluoride did not react under our conditions to form the acid **2a** (entry 1). With 4-tolyl chloride, an NMR yield of 38% was detected, while the bromide yielded 67% of the acid (NMR analysis). Curiously, with 4-tolyl iodide, the NMR yield of the acid was only 35%. 4-Tolyl iodide is a solid, while 4-tolyl bromide was always heated to its melting point (below 30 °C) for easier manipulation with a syringe. The group of Ondruschka addressed similar observations in mechanochemical Suzuki couplings with the aspect of limited mass transport superimposing the increased reactivity when moving down the halogenides in the periodic table.[10] Additionally, the use of aryl iodides or iodine in the activation step required an additional washing step with sodium sulfite or sodium thiosulfate solution in order to reduce any iodine, thereby decreasing the yield further.

Table S5. Effect of changing the organohalide starting material in a mechanochemical Grignard reaction with CO_2 in a planetary ball mill.

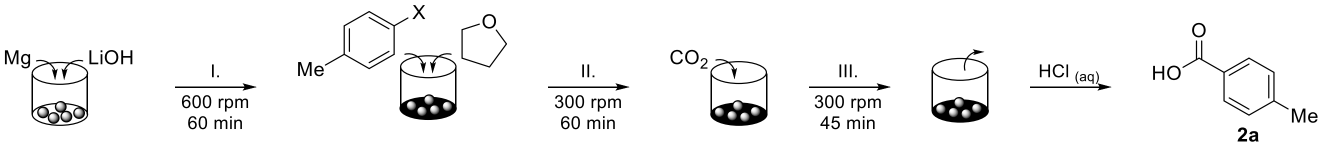

Entry[a]	X	NMR Yield of **2a** [%][b]
1	F	0
2	Cl	38
3	Br	67[c]
4	I	35

[a] Reaction conducted in a 20 mL ZrO_2-M milling vessel with gas inlet/outlet valves and 5 ZrO_2-M balls of 10 mm diameter precisely according to General Procedure 1, except for using THF instead of 2-MeTHF. [b] The NMR yield was determined as described in General Procedure 1, with yields after isolation in brackets in case they were determined. [c] Average over two reactions.

Miscellaneous test reactions

To confirm the optimal conditions, further test reactions were carried out and the remaining parameters were changed (Table S6). Since the highest yield of **2a** under optimum conditions was only 63%, it was suspected that the amount of CO_2 in the milling vessel might in fact not suffice for a full conversion. Therefore, the reaction was scaled down to 3 mmol of **1a** with the equivalents of all other reagents adjusted accordingly, but still using 4 bar of CO_2 (corresponding to more than 1 equiv. of CO_2). With 49% NMR yield, there was no improvement (entry 1). As a counter-proof, 6 and 8 bar of CO_2 were used in the standard 4 mmol scale reaction, yielding only 25 and 29% of **2a**, respectively (entries 2 and 3). To eliminate possibly detrimental influences of residual water on the milling equipment, the milling vessel and the balls were heated out prior to the reaction sequence, but no increase in yield was observed (entry 4). On the other hand, when the milling step for the Grignard formation was carried out without an argon atmosphere, a significant number of side reactions occurred which rendered the separation of **2a** from the resulting by-products rather complicated. As a control reaction, the procedure was carried out without magnesium, yielding no product. Instead, the starting material was recovered after workup (entry 6).

When the reaction was performed in the presence of 1.5 equiv. of TEMPO (2,2,6,6-tetramethylpiperidine-*N*-oxyl radical), the yield of **2a** was reduced to 40% (after isolation). According to results of mechanistic studies on the homocoupling of Grignard reagents by Studer and coworkers,[11] this outcome suggests that under mechanochemical conditions with severely reduced amounts of solvent the reaction of Grignard reagents with gaseous CO_2 predominantly proceeds via radical anions. Furthermore, the here employed aryl Grignard reagent is more stable than alkyl Grignard reagents and therefore less likely to undergo oxidation, thus preventing the formation of a TEMPO adduct.[12] The decrease in yield might be due to either the share of free radicals that are trapped with TEMPO (yet the resulting compounds could not be detected since they probably did not withstand the workup and separation procedures, if they had been generated in the first place), or a simple dilution of the reaction mixture in the vessel as the available volume for CO_2 gas was reduced further.

Table S6. Collected control reactions of a mechanochemical Grignard reaction of **1a** with CO_2 in a planetary ball mill.

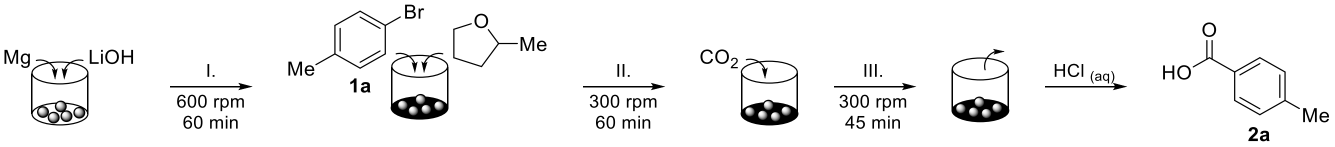

Entry[a]	Change to General Procedure 1	NMR Yield of **2a** [%][b]
1	Scaling the reaction down to 3 mmol of **1a** (all other reagents accordingly except for CO_2); THF instead of 2-MeTHF	49
2	Using 6 bar of CO_2 instead of 4 bar; THF instead of 2-MeTHF	25 (25)
3	Using 8 bar of CO_2 instead of 4 bar; THF instead of 2-MeTHF	29 (29)
4	Heating out the jar prior to conducting the reaction sequence	56
5	Heating out the jar prior to conducting the reaction sequence, milling under ambient atmosphere for step II	14[c]
6	Omitting magnesium (control reaction)	0[d]
7	Addition of 1.5 equiv. of TEMPO in step II	n.d (40)

[a] Reaction conducted in a 20 mL ZrO_2-M milling vessel with gas inlet/outlet valves and 5 ZrO_2-M balls of 10 mm diameter precisely according to General Procedure 1 with the noted changes. [b] The NMR yield was determined as described in General Procedure 1, with yields after isolation in brackets in case they were determined. [c] Many side products were formed. [d] Starting material obtained after workup.

Reproducibility issues

When the optimum reaction conditions described in General procedure 1 were tested with THF instead of 2-MeTHF for the first time, an NMR yield of 75% was detected, and after isolation, it was even found that 80% of the acid **2a** had formed (Table S7, entry 1). Unfortunately, attempts to repeat the experiment under the exact same conditions remained unsuccessful, and in each case lower yields ranging from 18-58% according to ^{1}H NMR spectroscopy with 1,3,5-trimethoxybenzene as the internal standard were found (entries 2-9). At first, these deviations in yield seemed inexplicable given that all conditions were kept unchanged: the same batches of reagents, the same equipment and the same (meticulous) cleaning procedures had been used. Only when the main insulation ring, the valve insulations and the inner valves were all exchanged with new ones, two experiments conducted parallel on the same day (such that humidity or temperature changes could not interfere) gave results with acceptable deviations (64 and 67% after isolation, entries 10 and 11). Therefore, gas leaks were certainly a reason for decreased yields if the insulation material had aged with repeated contact to chemicals.

With 2-MeTHF, the results proved more reproducible: a range from 41 to 63% yield (entries 12-14) after isolation was still large, yet the two lower yields of the compared three results could be explained with material lost during the flushing of the milling vessels with CO_2. This opened three more possible sources of error: the flushing process, the water content of the solvent and the detection of the yield through NMR spectroscopy with an internal standard. The possible loss of material during the flushing process was addressed by opening the milling vessel between steps II and III to liberate the valves from any residues that had blocked them during the milling process before flushing the grinding chamber with CO_2. To exclude errors from the water content of the solvents, 2-MeTHF with ≤ 20 ppm water in a sealed bottle was bought. The THF from the solvent purification system turned blue upon being subjected to sodium on an inorganic carrier (Solvona® from DR. BILGER UMWELTCONSULTING) and benzophenone to test for the water content (see in a video on our homepage: https://bolm.oc.rwth-aachen.de/sites/default/files/files/thf-sps.mp4).

Although several experiments in which both the NMR yield and the yield after isolation were determined suggested that using an internal standard to determine the yield through ^{1}H NMR spectroscopy was safe (e.g., Table S1 entries 5, 8 or 10; Table S6 entries 2 or 3), it was suspected that the combination of 1,3,5-trimethoxybenzene with

the product might occasionally be responsible for deviations like in entry 10. There, an NMR yield of 64% corresponded to 67% yield after isolation, which led us to question the efficiency of our yield detection method. It may not be unusual that yields after isolation were *lower*, but not higher than suggested by an NMR yield. Crude products with NMR yields below 20% were usually not selected for purification, but discarded. Thus, to exclude such errors in our investigation, the crude product was weighed, the mass was compared with the NMR yield for a rough estimation, and all entries were purified from there on.

Table S7. Attempts of reproducing the below milling conditions in a mechanochemical Grignard reaction of **1a** with CO_2 in a planetary ball mill.

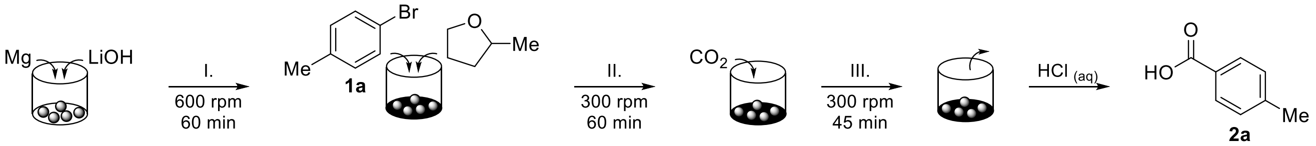

Entry[a]	Lewis base	NMR Yield of **2a** [%][b]
1	2.0 equiv. THF	75 (80)
2	2.0 equiv. THF	58
3	2.0 equiv. THF	34
4	2.0 equiv. THF	18
5	2.0 equiv. THF	51
6	2.0 equiv. THF	23
7	2.0 equiv. THF	58
8	2.0 equiv. THF	19
9	2.0 equiv. THF	31
10	2.0 equiv. THF[d]	64 (67)
11	2.0 equiv. THF[d]	70 (64)
12	2.0 equiv. 2-MeTHF	45 (41)[c]
13	2.0 equiv. 2-MeTHF	n.d. (56)[c]
14	2.0 equiv. 2-MeTHF	n.d. (63)

[a] Reaction conducted in a 20 mL ZrO_2-M milling vessel with gas inlet/outlet valves and 5 ZrO_2-M balls of 10 mm diameter precisely according to General Procedure 1. [b] The NMR yield was determined as described in General Procedure 1, with yields after isolation in brackets in case they were determined. [c] During the flushing of the milling vessel with CO_2, some material was lost in this reaction.[4] [d] New insulation rings and new valves were used.

In order to produce reproducible results, the following steps should therefore be closely adhered to: All insulation rings and inner valves should be exchanged regularly besides cleaning them with great care; the flushing process should always be preceded by liberating the holes in the lid from residues of the reaction mixture; the solvents should be checked for their dryness regularly as the absolute amount of water is more relevant than in larger solvent quantities; and determining the yield with ^{1}H NMR spectroscopy and internal standards seems unsuitable for carboxylic acids. Other purification methods than column chromatography were also tested (see below).

Alternative purification methods to column chromatography

Purification 1: Filtration over activated charcoal

General Procedure 1 was followed until the determination of the yield by NMR spectroscopy. After that, the crude product was dispersed in 2 M aqueous NaOH solution, stirred with 2 g of activated charcoal for 2 min, filtered over celite, and the pH of the filtrate was adjusted to 1 with 10% aqueous HCl (tested with pH paper strips). This solution was extracted with 3 x 35 mL of diethyl ether; the combined organic phases were dried over magnesium sulfate, and the solvent was evaporated. The product was then weighed.

Table S8. Results of purifying **2a** according to Purification 1 compared to the NMR yields.

Entry[a]	NMR Yield of **2a** [%][b]	Yield of **2a** after purification 1 [%][b]
1	30	7
2	43	20
3	37	26

[a] Reaction conducted in a 20 mL ZrO_2-M milling vessel with gas inlet/outlet valves and 5 ZrO_2-M balls of 10 mm diameter according to General Procedure 1. [b] The NMR yield was determined as described in General Procedure 1.

Purification 2: Reaction with (trimethylsilyl)diazomethane and isolation as the respective carboxylic acid methyl ester

In a 100 mL Schlenk round bottom flask equipped with a magnetic stirring bar and a septum, 4-toluic acid (545 mg, 4.0 mmol) was added and heated under a flow of argon. Dry methanol (16 mL) and dry diethyl ether (60 mL) were added while stirring. When all 4-toluic acid had dissolved, (trimethylsilyl)diazomethane (2 M in diethyl ether, 2.2 mL, 4.4 mmol, 1.1 equiv.) was added in the dark, and the mixture was left to stir for 20 h overnight at ambient temperature covered with a card box. After control by TLC for full conversion, the mixture was poured into a 250 mL round bottom flask, the solvent was evaporated, Celite was added and the mixture was purified by column chromatography over silica gel (45 cm length, 5 cm diameter, pure *n*-pentane to diethyl ether/*n*-pentane 1:99 *v/v*) to yield methyl 4-toluate (409 mg, 2.72 mmol, 68% yield).

Discussion

During the purification over activated charcoal, significant amounts of product were lost when compared to the NMR yields. The losses after column chromatography were less significant in comparison. Instead, the esterification of **2a** with (trimethylsilyl)diazomethane was tried to simplify the subsequent column chromatography. While for reactions in solution involving carboxylic acids, this reagent can safely be employed before the workup by simple addition to the reaction mixture, it is less suitable for Grignard reactions in general and the ball milling setup in specific: here, it can only be used after workup through extraction and extensive drying of the crude product. Additionally, the yield of only 68% in the exemplary reaction was probably caused by the high volatility of the product and contrasted the expected quantitative conversion towards the ester. To conclude the efforts for other purification methods, isolation by column chromatography as described in the general procedures was applied.

Alternative quenching methods to acid workup

Quenching 1: Addition of benzyl bromide

General Procedure 1 was followed until the third milling step was finished and the pressure was relieved. Then, benzyl bromide (478 μL, 684 mg, 4.0 mmol, 1.0 equiv.) was added, the vessel was closed again, flushed with CO_2 and the mixture was milled at 300 rpm for 60 min. The lid, main insulation ring, the balls, and the milling container

were washed with 10% aqueous HCl and ethyl acetate until all black and metallic residues were removed.* The two-phase mixture was transferred into a 250 mL separating funnel, the phases were separated, the aqueous phase was brought to pH 8 through addition of $NaHCO_3$ and extracted with 2 x 75 mL ethyl acetate. The combined organic layer was dried over $MgSO_4$, and the solvent was evaporated under reduced pressure (160 mbar, 40 °C bath temperature) to dryness. A ^{1}H NMR spectrum was recorded.

**Note:* *Before the lid could come into contact with HCl or ethyl acetate, the valves and their insulation rings were removed, taken apart and cleaned thoroughly with ethanol (technical grade) and a lint-free paper towel. After the removal of all residues from the milling equipment, the lid, main insulation ring, balls, and milling vessel were cleaned with water, little scouring agent, and acetone, and left to dry at ambient atmosphere.*

Quenching 2: Addition of diethylamine

As described under “Quenching 1: Addition of benzyl bromide” with the use of diethylamine (621 μL, 439 mg, 6.0 mmol, 1.5 equiv.) instead of benzyl bromide.

Discussion

To expand the scope of possible products, the reaction was quenched with the above mentioned reagents, namely benzyl bromide and diethylamine. It was thereby hoped to access other products, namely esters or amides, respectively. Unfortunately, after both reactions, only acid **2a** was detected in the ^{1}H NMR spectrum of the crude products.

Optimization of the milling conditions with sodium methyl carbonate in the mixer mill

In the attempts to conduct a mechanochemical Grignard reaction with sodium methyl carbonate (SMC), it could be profited from the optimization in the setup with CO_2 regarding the employment of 1.1 equiv. of lithium hydroxide and of 2.0 equiv. of THF (Table S9). Since no more gaseous CO_2 was needed in the reactions with SMC, the milling vessels with gas inlet and outlet valves could be replaced by a smaller mixer mill vessel with only one ball. Furthermore, since SMC was applied during the Grignard formation step, no third milling sequence was required. However, with THF we also encountered reproducibility issues as shown in entries 1-3. When the NMR yield was determined, the crude product was weighed to compare whether the yield detected by NMR spectroscopy was higher than the mass of crude product would allow (indicated as *wr* in the table). For the magnesium activation step, 60 min were sufficient (entry 4), yet with shorter overall reaction times, THF performed overall worse (entries 1-9). Similar to the planetary mill setup, a lower frequency sufficed for the second milling step (entry 8). With THF, combining the activation, the Grignard formation, and the reaction with SMC in one step still yielded 43% of **2a**.
In reactions with 2-MeTHF, however, which had been the solvent of choice for the reactions with CO_2 in the planetary mill, reaction times could be shortened to 45 min with beneficial effects for the yield (entries 11-18). By comparing with the performance of lithium chloride (entry 11), lithium hydroxide was confirmed as the more suitable agent for the magnesium insertion reaction (entry 12). Lastly, with 2-MeTHF, a separate addition of SMC was tested, but only 12% of the acid were found after purifying the crude product (entry 18).
With the addition of 1.5 equiv. of TEMPO as radical trap, interestingly, the reaction with SMC towards the acid was completely suppressed (entry 19). This contrasts the results obtained with gaseous CO_2 (see Table S6, entry 7), where TEMPO only reduced the yield to 40%. Indeed, this eradication of reactivity with an SET trap suggests that free radicals play a more significant role in the procedure with SMC than in the one with CO_2.

Table S9. Effect of changing standard conditions in a mechanochemical Grignard reaction of **1a** with sodium methyl carbonate (SMC) in a mixer ball mill.

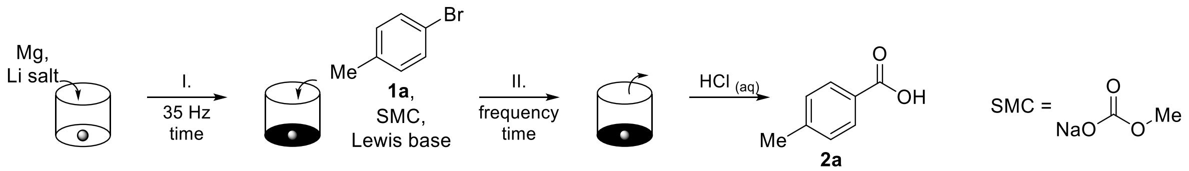

Entry[a]	Step I: Li-salt	Step I:[b] Mg activation	Step II: Lewis base	Step II: Acid formation	NMR Yield of **2a** [%][c]
1	1.1 equiv. LiOH	60 min	2.0 equiv. THF	20 Hz, 120 min	*wr*[d] (59)
2	1.1 equiv. LiOH	60 min	2.0 equiv. THF	20 Hz, 120 min	69 (38)
3	1.1 equiv. LiOH	60min	2.0 equiv. THF	20 Hz, 120 min	n.d. (33)
4	1.1 equiv. LiOH	90 min	2.0 equiv. THF	20 Hz, 120 min	*wr*[d] (52)
5	1.1 equiv. LiOH	60 min	2.0 equiv. THF	20 Hz,105 min	59
6[e]	1.1 equiv. LiOH	60 min	2.0 equiv. THF	20 Hz, 90 min	68 (54)
7	1.1 equiv. LiOH	60 min	2.0 equiv. THF	20 Hz, 90 min	66 (41)
8	1.1 equiv. LiOH	60 min	2.0 equiv. THF	25 Hz, 90 min	*wr*[d] (52)
9	1.1 equiv. LiOH	60 min	2.0 equiv. THF	20 Hz, 60 min	40
10	1.1 equiv. LiOH	>	2.0 equiv. THF	20 Hz, 120 min	50 (43)
11	1.1 equiv. LiCl	60 min	2.0 equiv. 2-MeTHF	20 Hz, 120 min	n.d. (15)
12	1.1 equiv. LiOH	60 min	2.0 equiv. 2-MeTHF	20 Hz, 120 min	n.d. (24)
13	1.1 equiv. LiOH	60 min	2.0 equiv. 2-MeTHF	20 Hz, 105 min	n.d. (28)
14	1.1 equiv. LiOH	60 min	2.0 equiv. 2-MeTHF	20 Hz, 90 min	n.d. (34)
15	1.1 equiv. LiOH	60 min	2.0 equiv. 2-MeTHF	20 Hz, 75 min	n.d. (38)
16	1.1 equiv. LiOH	60 min	2.0 equiv. 2-MeTHF	20 Hz, 60 min	n.d. (42)
17	1.1 equiv. LiOH	60 min	2.0 equiv. 2-MeTHF	20 Hz, 45 min	n.d. (45)
18[f]	1.1 equiv. LiOH	60 min	2.0 equiv. 2-MeTHF	2 x (20 Hz, 60 min)	12
19[g]	1.1 equiv. LiOH	60 min	2.0 equiv. 2-MeTHF	20 Hz, 45 min	n.d. (0)

[a] Reaction conducted in a 10 mL ZrO_2-M milling vessel with 1 ZrO_2-M ball of 10 mm diameter using 1 mmol of **1a** according to General procedure 2. ">" signifies that this step was combined with step II under the conditions written there. [b] The activation of magnesium with the indicated amount of lithium salt was conducted at 35 Hz for the indicated amount of time. [c] The NMR yield was determined as described in General Procedure 2, with yields after isolation in brackets in case they were determined. [d] "wr" signifies that the NMR yield was higher than the mass of crude product would allow. In these cases, the yield was always determined after purification through column chromatography (given in brackets). [e] 1.0 equiv. of SMC were used instead of 1.5 equiv. [f] SMC was added for a separate grinding step after adding and milling **1a** and the Lewis base (step I: Mg + LiOH, step II: add **1a** + 2-MeTHF, step III: add SMC). [g] In step II, TEMPO (2,2,6,6-tetramethylpiperidine-*N*-oxyl radical, 234 mg, 1.5 mmol, 1.5 equiv.) was added.

Optimization of the milling conditions with sodium methyl carbonate in the planetary mill

To further test the tolerance of the protocol towards different milling conditions, SMC was used in a planetary ball mill. Some control reactions confirmed what was already known from reactions in the mixer mill: THF required longer reaction times than 2-MeTHF (compare entries 1-5 with 6-10). Additionally, the impact of the size of the milling balls was checked by replacing the balls of 9 mm average diameter with the same weight of 6 mm average diameter balls, yet the yield decreased in comparison to the best result (10%, entry 11 compared to 40%, entry 8). Similarly, the addition of iodine in the activation step did not help (24%, entry 12). Lastly, with lower yields for either 1.0 or 2.0 equiv. of SMC in comparison to the hitherto used 1.5 equiv., it was decided to use the conditions in entry 8 to test the substrate scope with SMC.

Table S10. Effect of changing standard conditions in a mechanochemical Grignard reaction of **1a** with sodium methyl carbonate (SMC) in a planetary ball mill.

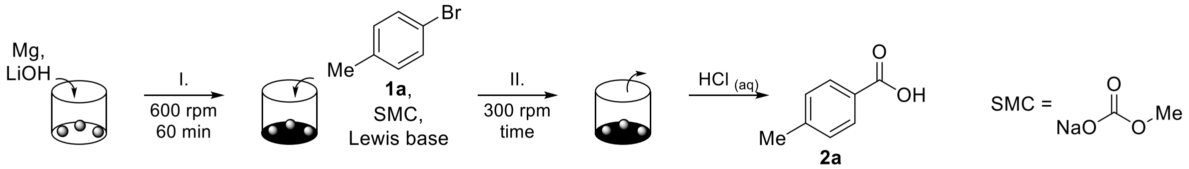

Entry[a]	Step II: SMC	Step II: Lewis base	Step II: Acid formation	Yield of **2a** [%][b]
1	1.5 equiv.	2.0 equiv. THF	60 min	53
2	1.5 equiv.	2.0 equiv. THF	60 min	48
3	1.5 equiv.	2.0 equiv. THF	45 min	35
4	1.5 equiv.	2.0 equiv. THF	30 min	37
5	1.5 equiv.	2.0 equiv. THF	15 min	38
6	1.5 equiv.	2.0 equiv. 2-MeTHF	60 min	40
7	1.5 equiv.	2.0 equiv. 2-MeTHF	60 min	40
8	1.5 equiv.	2.0 equiv. 2-MeTHF	45 min	40
9	1.5 equiv.	2.0 equiv. 2-MeTHF	30 min	38
10	1.5 equiv.	2.0 equiv. 2-MeTHF	15 min	38
11[c]	1.5 equiv.	2.0 equiv. 2-MeTHF	45 min	10
12[d]	1.5 equiv.	2.0 equiv. 2-MeTHF	45 min	24
13	1.0 equiv.	2.0 equiv. 2-MeTHF	45 min	31
14	2.0 equiv.	2.0 equiv. 2-MeTHF	45 min	32

[a] Reaction conducted in a 12 mL ZrO_2-M milling vessel with 3 ZrO_2-M balls of 9 mm diameter using 1 mmol of **1a** according to General procedure 3. [b] Yields after purification through column chromatography. [c] 18 ZrO_2-Y balls of 5 mm diameter were used instead of the 3 ZrO_2-M balls (both groups of balls had approximately the same weight). [d] One flake of iodine was added during the activation step.

Explanation of the ketone formation

As discussed in the main article, the formation of ketones was initially unexpected. However, similar results have previously been investigated with organolithium reagents, including approaches through flow chemistry involving Grignard reagents in a first step to obtain unsymmetrical ketones.[13] To selectively obtain ketones from carboxylic acids, carboxylates, or CO_2, a transformation into e.g., Weinreb amides or the addition of other reagents was necessary.[14] In our case, however, this reaction occurred without any of such additives. Thus, we assume that the formation of ketones stems from 1) the stability of the di-magnesium salt **6** that is similar to or lower than that of described di-lithium salts,[13a] and 2) the lack of solvents in this mechanochemical approach (Scheme 3). In solution,

solvatisation enables the diffusion of the magnesium halides so that an electrophilic centre is recreated at the central carbon atom. This carbon atom is then attacked by remaining organomagnesiums to form carbinol **7**. In the grinding vessel, however, only two equivalents of solvent are available, hence such solvatisation is prevented. Owing to the supposed stability of the salt **6** in the absence of solvatisation, it is hydrolysed slower than any excess organomagnesium, so no carbinol **7** is formed before or during the workup. Moreover, a methoxy group in *o*-position can act as a chelating unit leading to a favourable six-membered complex like in Weinreb reactions and stabilising salt **6** further, thereby explaining the significant amounts of ketones formed with methoxy substituents.

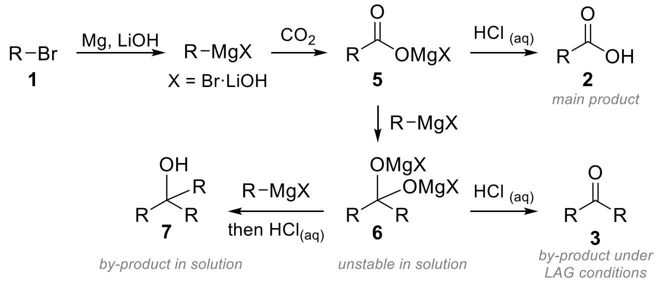

Scheme S1. Suggested mechanism for the formation of carboxylic acids **2** and symmetric ketones **3** from aryl halides and carbon dioxide in a mechanochemical Grignard reaction and the respective pathway towards carbinols **7** in solution.

List of unsuccessful substrates and discussion

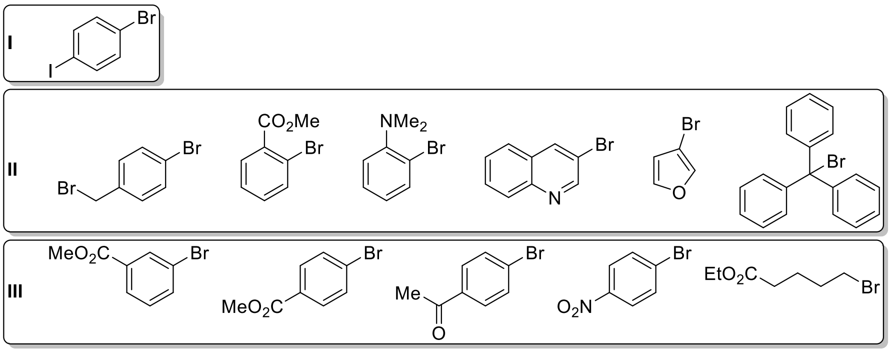

Scheme S2. List of unsuccessful substrates in separate groups: (I) decomposition during workup and purification; (II) complex mixture after workup; (III) starting material obtained after workup.

While striving to broaden the scope of possible substrates, it was found that some substrates did not undergo the reaction as anticipated and are therefore not reported in the main article (Scheme S2). According to the reaction results, they could be classified into three different groups. “Group” **I** included only 1-bromo-4-iodobenzene, of which 4-bromobenzoic acid was isolated in approximately 24% yield with some impurities. This result matches assumptions made in the discussion of alternative substrates (other organohalides), as the magnesium insertion reaction seemed to have occurred at the expectedly more reactive iodide site. Then, however, the formation of significant amounts of iodine was observed during the workup and purification procedures, allowing for no definite conclusion on whether 4-iodobenzoic acid was formed and decomposed under the influence of light before detection. For all substrates listed within group **II**, complex mixtures were obtained after workup. Neither product nor ketone nor the starting material could often be identified in the crude ^{1}H NMR spectrum.
The compounds shown in group **III** did not react. Among them were esters and other substrates with electron-withdrawing substituents. Independent of the substitution pattern, the insertion reaction did not take place, and after all milling steps and workup, only starting material was obtained for the shown substrates. Since even aliphatic ethyl 5-bromovalerate did not show traces of reactivity, steric reasons as well as a connection to the hybridization of the carbon atom in question can be ruled out. Because with 4’-bromoacetophenone no magnesium insertion was observed, the integrity the keto functionality of this molecule could not be investigated. A nitro group in the backbone of the aryl ring also prevented the insertion reaction.

Characterization of carboxylic acids, ketones, and other by-products

4-Methylbenzoic acid (2a)

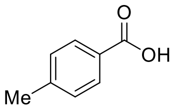

The title compound was prepared according to General procedure 1 from 4-tolyl bromide (684 mg, 492 µL, 4.0 mmol) and obtained as a colorless solid after column chromatography (diethyl ether/*n*-pentane with traces of acetic acid 1:9 to 1:4 *v/v*), UV active, 343 mg (2.52 mmol, 63% yield). **^{1}H NMR** (600 MHz, $CDCl_3$) δ: 12.77 (br, 1H), 8.02 (d, *J* = 8.2 Hz, 2H), 7.28 (d, *J* = 8.2 Hz, 2H), 2.44 (s, 3H) ppm. **^{13}C{^{1}H} NMR** (151 MHz) $CDCl_3$ δ: 172.7, 144.8, 130.4, 129.4, 126.8, 21.9 ppm.
The NMR data are in accordance with those presented in literature.[15]

2-Methylbenzoic acid (2b)

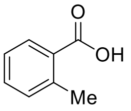

The title compound was prepared according to General procedure 1 from 2-tolyl bromide (684 mg, 481 µL, 4.0 mmol) and obtained as an off-white solid after column chromatography (diethyl ether/*n*-pentane with traces of acetic acid 1:9 *v/v*), UV active, 364 mg (2.67 mmol, 67% yield). **^{1}H NMR** (600 MHz, $CDCl_3$) δ: 12.47 (br, 1H), 8.09 (d, *J* = 7.8 Hz, 1H), 7.46 (t, *J* = 7.5 Hz, 1H), 7.29 (t, *J* = 7.8 Hz, 2H), 2.68 (s, 3H) ppm. **^{13}C{^{1}H} NMR** (151 MHz, $CDCl_3$) δ: 173.5, 141.5, 133.1, 132.1, 131.7, 128.5, 126.0, 22.3 ppm.
The NMR data are in accordance with those presented in literature.[16]

4-(Trifluoromethyl)benzoic acid (2c)

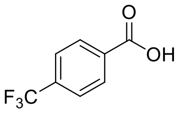

The title compound was prepared according to General procedure 1 from 1-bromo-4-(trifluoro-methyl)benzene (900 mg, 560 µL, 4.0 mmol) and obtained as a colorless solid after column chromatography (diethyl ether/*n*-pentane with traces of acetic acid 15:85 to 1:4 *v/v*), UV active, 543 mg (2.86 mmol, 71% yield). **^{1}H NMR** (600 MHz, acetone-d_6) δ: 11.68 (br, 1H), 8.24 (d, *J* = 8.0 Hz, 2H), 7.88 (t, *J* = 8.0 Hz, 2H) ppm. **^{13}C{^{1}H} NMR** (151 MHz, acetone-d_6) δ: 166.5, 135.2, 134.6 (q, *J* = 32.3 Hz), 131.3, 126.5 (q, *J* = 3.9 Hz), 125.0 (q, *J* = 271.9 Hz) ppm. **^{19}F NMR** (565 MHz, acetone-d_6) δ: -63.62 ppm.
The NMR data are in accordance with those presented in literature. [16]

4-Chlorobenzoic acid (2d)

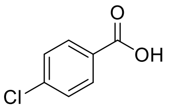

The title compound was prepared according to General procedure 1 from 1-bromo-4-chloro-benzene (766 mg, 4.0 mmol) and obtained as a colorless solid after column chromatography (diethyl ether/*n*-pentane with traces of acetic acid 15:85 to 1:4 *v/v*), UV active, 306 mg (1.95 mmol, 49% yield). **^{1}H NMR** (600 MHz, acetone-d_6) δ: 11.43 (br, 1H), 7.91 (d, *J* = 8.5 Hz, 2H), 7.42 (d, *J* = 8.5 Hz, 2H) ppm. **^{13}C{^{1}H} NMR** (151 MHz, acetone-d_6) δ: 166.8, 139.7, 132.3, 130.4, 129.8 ppm.
The NMR data are in accordance with those presented in literature. [16]

4-Fluorobenzoic acid (2e)

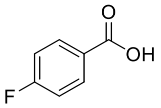

The title compound was prepared according to General procedure 1 from 1-bromo-4-fluorobenzene (700 mg, 439 µL, 4.0 mmol) and obtained as a colorless solid after column chromatography (diethyl ether/*n*-pentane with traces of acetic acid 1:9 to 15:85 *v/v*), UV active, 286 mg (2.04 mmol, 51% yield). **^{1}H NMR** (600 MHz, $CDCl_3$) δ: 12.38 (br, 1H), 8.14 (dd, *J* = 8.5, 5.4 Hz, 2H), 7.16 (t, *J* = 8.5 Hz, 2H) ppm. **^{13}C{^{1}H} NMR** (151 MHz, $CDCl_3$) δ: 171.4, 166.5 (d, *J* = 255.4 Hz), 133.1 (d, *J* = 9.6 Hz), 125.6 (d, *J* = 3.0 Hz), 115.9 (d, *J* = 22.1 Hz) ppm. **^{19}F NMR** (565 MHz, $CDCl_3$) δ: –104.01 ppm.
The NMR data are in close accordance with those presented in literature.[17]

4-tert-Butylbenzoic acid (2f)

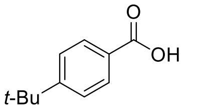

The title compound was prepared according to General procedure 1 from 1-bromo-4-*tert*-butylbenzene (852 mg, 638 μL, 4.0 mmol) and obtained as a colorless solid after column chromatography (diethyl ether/*n*-pentane with traces of acetic acid 1:9 to 15:85 *v/v*), UV active, 300 mg (1.68 mmol, 42% yield). **^{1}H NMR** (600 MHz, $CDCl_3$) δ: 12.09 (br, 1H), 8.05 (d, *J* = 7.6 Hz, 2H), 7.50 (d, *J* = 7.6 Hz, 2H), 1.36 (s, 9H) ppm. **^{13}C{^{1}H} NMR** (151 MHz, $CDCl_3$) δ: 172.0, 157.7, 130.3, 126.6, 125.6, 35.3, 31.3 ppm.
The NMR data are in accordance with those presented in literature.[18]

4-Thiomethylbenzoic acid (2g)

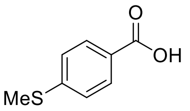

The title compound was prepared according to General procedure 1 from 4-bromothioanisole (812 mg, 4.0 mmol) and obtained as a bright yellow solid after column chromatography (diethyl ether/*n*-pentane with traces of acetic acid 1:4 to 1:1 *v/v*), UV active, 387 mg (2.30 mmol, 58% yield). **^{1}H NMR** (600 MHz, $CDCl_3$) δ: 8.00 (d, *J* = 8.6 Hz, 2H), 7.28 (d, *J* = 8.6 Hz, 2H), 2.53 (s, 3H) ppm. **^{13}C{^{1}H} NMR** (151 MHz, $CDCl_3$) δ: 171.4, 146.9, 130.6, 125.4, 125.1, 14.9 ppm.
The ^{1}H NMR data are in accordance with those presented in literature.[16]

2-Thiomethylbenzoic acid (2h)

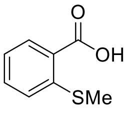

The title compound was prepared according to General procedure 1 from 2-bromothioanisole (812 mg, 534 μL, 4.0 mmol) and obtained as colorless crystals after column chromatography (diethyl ether/*n*-pentane with traces of acetic acid 15:85 to 3:7 *v/v*), UV active, 448 mg (2.66 mmol, 67% yield). **^{1}H NMR** (600 MHz, $CDCl_3$) δ: 8.15 (dd, *J* = 7.9 , 1.6 Hz, 1H), 7.55-7.51 (m, 1H), 7.32-7.29 (m, 1H), 7.22-7.18 (m, 1H), 2.48 (s, 3H) ppm. **^{13}C{^{1}H} NMR** (151 MHz, $CDCl_3$) δ: 171.4, 146.9, 130.6, 125.4, 125.1, 14.9 ppm.
The ^{1}H NMR patterns are in accordance with those presented in literature.[19]

2,3,4,5,6-Pentafluorobenzoic acid (2i)

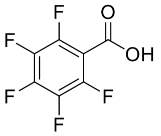

The title compound was prepared according to General procedure 1 from bromopentafluorobenzene (988 mg, 499 μL, 4.0 mmol) and obtained as a beige solid after flash chromatography (diethyl ether/*n*-pentane with traces of acetic acid 1:4 *v/v*), barely UV active, 430 mg (2.03 mmol, 51% yield). **^{1}H NMR** (600 MHz, acetone-d_6) δ: 11.39 (br, 1H) ppm. **^{13}C{^{1}H} NMR** (151 MHz, acetone-d_6) δ: 159.6, 145.9 (dm, $^1J_{C–F}$ = 254.7 Hz), 143.5 (dm, $^1J_{C–F}$ = 255.5 Hz), 138.4 (dm, $^1J_{C–F}$ = 251.0 Hz), 109.3 (td, *J* = 16.8, 3.8 Hz) ppm. **^{19}F NMR** (564 MHz, acetone-d_6) δ: -141.00 (m, 2F), -153.45 (tt, *J* = 20.5, 4.3 Hz, 1F), -163.47 (m, 2F) ppm.
The ^{1}H NMR patterns are in accordance with those presented in literature.[20]

2,4,6-Tri-iso-propylbenzoic acid (2j)

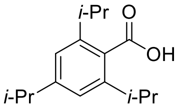

The title compound was prepared according to General procedure 1 from 2-bromo-1,3,5-tri-*iso*-propylbenzene (1133 mg, 1010 μL, 4.0 mmol) and obtained as a colorless solid after column chromatography (diethyl ether/*n*-pentane with traces of acetic acid 15:85 to 1:4 *v/v*), UV active, 521 mg (2.10 mmol, 52% yield). **^{1}H NMR** (600 MHz, $CDCl_3$) δ: 10.85 (br, 1H), 7.05 (s, 2H), 3.06 (sept., *J* = 6.8 Hz, 2H), 2.91 (sept., *J* = 6.9 Hz, 1H), 1.29 (d, *J* = 6.8 Hz, 12H), 1.26 (d, *J* = 6.9 Hz, 6H) ppm. **^{13}C{^{1}H} NMR** (151 MHz, $CDCl_3$) δ: 175.7, 150.7, 145.0, 129.4, 121.1, 34.6, 31.7, 24.4, 24.1 ppm.
The ^{1}H NMR data are in accordance with those presented in literature.[21]

2-Phenylacetic acid (2k)

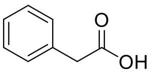

The title compound was prepared according to General procedure 1 from benzyl bromide (684 mg, 478 μL, 4.0 mmol) and obtained as a colorless crystalline solid after column chromatography (diethyl ether/*n*-pentane with traces of acetic acid 1:9 to 1:4 *v/v*), stains with CAM, 445 mg (3.27 mmol, 81% yield). **^{1}H NMR** (600 MHz, $CDCl_3$) δ: 11.34 (br, 1H), 7.37-7.27 (m, 5H), 3.66 (s, 2H) ppm. **^{13}C{^{1}H} NMR** (151 MHz, $CDCl_3$) δ: 177.9, 133.4, 129.5, 128.8, 127.5, 41.5 ppm.
The NMR data are in accordance with those presented in literature.[22]

4-Phenylbutanoic acid (2l)

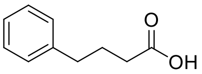

The title compound was prepared according to General procedure 1 from 1-bromo-3-phenylpropane (796 mg, 608 μL, 4.0 mmol) and obtained as a colorless crystalline solid after column chromatography (diethyl ether/*n*-pentane with traces of acetic acid 1:9 to 1:4 *v/v*), stains with CAM, 471 mg (2.87 mmol, 72% yield). **^{1}H NMR** (600 MHz, $CDCl_3$) δ: 11.60 (br, 1H), 7.30 (t, *J* = 7.6 Hz, 2H), 7.22-7.18 (m, 3H), 2.69 (t, *J* = 7.6 Hz, 2H), 2.39 (t, *J* = 7.5 Hz, 2H), 1.98 (p, *J* = 7.5 Hz, 2H) ppm. **^{13}C{^{1}H} NMR** (151 MHz, $CDCl_3$) δ: 180.0, 141.3, 128.63, 128.57, 126.2, 35.1, 33.4, 26.3 ppm.
The NMR data are in accordance with those presented in literature.[15]

2-Thiophenecarboxylic acid (2m)

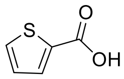

The title compound was prepared according to General procedure 3 from 2-bromothiophene (163 mg, 94 μL, 1.0 mmol) and obtained as an off-white crystalline solid after column chromatography (diethyl ether/*n*-pentane with traces of acetic acid 15:85 to 1:1 *v/v*), UV active, 80 mg (0.62 mmol, 62% yield). **^{1}H NMR** (600 MHz, $CDCl_3$) δ: 7.91 (dd, *J* = 3.8, 1.3 Hz, 1H), 7.66 (dd, *J* = 5.0, 1.3 Hz, 1H), 7.15 (dd, *J* = 5.0, 3.8 Hz, 1H) ppm. **^{13}C{^{1}H} NMR** (151 MHz, $CDCl_3$) δ: 168.0, 135.2, 134.2, 133.0, 128.2 ppm.
The ^{1}H NMR data are in accordance with those presented in literature.[16]

2-Naphthoic acid (2n)

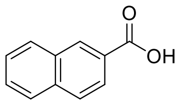

The title compound was prepared according to General procedure 1 from 2-bromonaphthalene (828 mg, 4.0 mmol) and obtained as an off-white crystalline solid after column chromatography (diethyl ether/*n*-pentane with traces of acetic acid 15:85 to 1:4 *v/v*), UV active, 300 mg (1.74 mmol, 44% yield). **^{1}H NMR** (600 MHz, $CDCl_3$) δ: 8.74 (s, 1H), 8.14 (dd, *J* = 8.6, 1.7 Hz, 1H), 8.00 (d, *J* = 8.2 Hz, 1H), 7.92 (dd, *J* = 11.1, 8.3 Hz, 2H), 7.63 (ddd, *J* = 8.2, 6.8, 1.3 Hz, 1H), 7.58 (ddd, *J* = 8.1, 6.9, 1.3 Hz, 1H) ppm. **^{13}C{^{1}H} NMR** (151 MHz, $CDCl_3$) δ: 172.3, 136.1, 132.6, 132.3, 129.7, 128.8, 128.5, 128.0, 126.9, 126.7, 125.5 ppm.
The ^{1}H NMR data are in accordance with those presented in literature.[23]

Cyclohexanecarboxylic acid (2o)

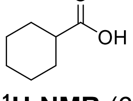

The title compound was prepared according to General procedure 1 from bromocyclohexane (652 mg, 493 μL, 4.0 mmol) and obtained as a beige liquid after column chromatography (diethyl ether/*n*-pentane with traces of acetic acid 15:85 to 1:4 *v/v*), stains with CAM, 212 mg (1.65 mmol, 41% yield). **^{1}H NMR** (600 MHz, $CDCl_3$) δ: 11.69 (br, 1H), 2.33 (tt, *J* = 11.3, 3.7 Hz, 1H), 1.94 (dd, *J* = 13.3, 3.7 Hz, 2H), 1.76 (dt, *J* = 12.8, 3.6 Hz, 2H), 1.67-1.62 (m, 1H), 1.50-1.41 (m, 2H), 1.33-1.19 (m, 3H) ppm. **^{13}C{^{1}H} NMR** (151 MHz, $CDCl_3$) δ: 182.7, 43.1, 28.9, 25.8, 25.5 ppm.
The NMR data are in accordance with those presented in literature.[24]

1-Adamantanecarboxylic acid (2p)

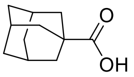

The title compound was prepared according to General procedure 1 from 1-bromoadamantane (861 mg, 4.0 mmol) and obtained as a colorless solid after column chromatography (diethyl ether/*n*-pentane with traces of acetic acid 1:9 to 15:85 *v/v*), stains with CAM, 177 mg (0.98 mmol, 25% yield). ^{1}**H NMR** (600 MHz, $CDCl_3$) δ: 11.78 (br, 1H), 2.03 (s, 3H), 1.91 (d, *J* = 2.4 Hz, 6H), 1.72 (q, *J* = 12.3 Hz, 6H) ppm. ^{13}**C{^{1}H} NMR** (151 MHz, $CDCl_3$) δ: 184.3, 40.6, 38.7, 36.6, 28.0 ppm.
The NMR data are in accordance with those presented in literature.[24]

4-Methoxybenzoic acid (2q)

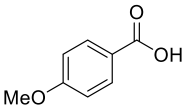

The title compound was prepared according to General procedure 1 from 4-bromoanisole (748 mg, 501 μL, 4.0 mmol) and obtained as a yellow solid after column chromatography (diethyl ether/*n*-pentane with traces of acetic acid 15:85 to 1:4 *v/v*), UV active, 359 mg (2.36 mmol, 59% yield). ^{1}**H NMR** (600 MHz, $CDCl_3$) δ: 12.20 (br, 1H), 8.07 (d, *J* = 8.8 Hz, 2H), 6.95 (d, *J* = 8.8 Hz, 2H), 3.88 (s, 3H) ppm. ^{13}**C{^{1}H} NMR** (151 MHz, $CDCl_3$) δ: 171.7, 164.2, 132.5, 121.8, 113.9, 55.6 ppm.
The NMR data are in accordance with those presented in literature.[17]

3-Methoxybenzoic acid (2r)

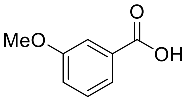

The title compound was prepared according to General procedure 1 from 3-bromoanisole (748 mg, 509 μL, 4.0 mmol) and obtained as an off-white solid after column chromatography (diethyl ether/*n*-pentane with traces of acetic acid 1:9 to 1:4 *v/v*), UV active, 242 mg (1.59 mmol, 40% yield). ^{1}**H NMR** (600 MHz, $CDCl_3$) δ: 7.73 (d, *J* = 7.8 Hz, 1H), 7.63 (s, 1H), 7.39 (t, *J* = 7.9 Hz, 1H), 7.17 (dd, *J* = 8.2, 2.6 Hz, 1H), 3.88 (s, 3H) ppm. ^{13}**C{^{1}H} NMR** (151 MHz, $CDCl_3$) δ: 172.2, 159.8, 130.7, 129.7, 122.8, 120.6, 114.6, 55.6 ppm.
The NMR data are in accordance with those presented in literature. [16]

2-Methoxybenzoic acid (2s)

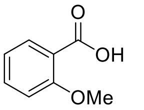

The title compound was prepared according to General procedure 3 from 2-bromoanisole (187 mg, 125 μL, 1.0 mmol) and obtained as a colorless solid after column chromatography (diethyl ether/*n*-pentane with traces of acetic acid 1:4 to 1:1 *v/v*), UV active, 38 mg (0.25 mmol, 25% yield). ^{1}**H NMR** (600 MHz, $CDCl_3$) δ: 10.83 (br, 1H), 8.17 (dd, *J* = 7.8, 1.7 Hz, 1H), 7.59-7.55 (m, 1H), 7.13 (t, *J* = 7.6 Hz, 1H), 7.06 (d, *J* = 8.4 Hz, 1H), 4.07 (s, 3H) ppm. ^{13}**C{^{1}H} NMR** (151 MHz, $CDCl_3$) δ: 165.7, 158.2, 135.2, 133.9, 122.3, 117.8, 111.8, 56.8 ppm.
The NMR data are in accordance with those presented in literature.[16]

3,3'-Dimethoxybenzophenone (3r)

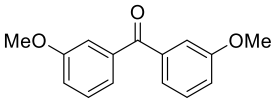

The title compound was isolated alongside **2o** as a colorless solid, UV active, 48 mg (0.20 mmol, 10% yield). ^{1}**H NMR** (600 MHz, $CDCl_3$) δ: 7.40-7.32 (m, 6H), 7.13 (ddt, *J* = 7.8, 2.3, 1.0 Hz, 2H), 3.86 (s, 6H) ppm. ^{13}**C{^{1}H} NMR** (151 MHz, $CDCl_3$) δ:196.4, 159.7, 139.1, 129.3, 123.0, 119.0, 114.5, 55.6 ppm.
The NMR data are in accordance with those presented in literature.[25]

2,2'-Dimethoxybenzophenone (3s)

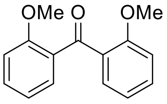

The title compound was prepared according to General procedure 1 from 2-bromoanisole (748 mg, 498 μL, 4.0 mmol) and obtained as a colorless solid after column chromatography (diethyl ether/*n*-pentane with traces of acetic acid 5:95 to 1:1 *v/v*), UV active, 129 mg (0.53 mmol, 27% yield). ^{1}**H**

NMR (600 MHz, $CDCl_3$) δ: 7.51 (dd, *J* = 7.6, 1.7 Hz, 2H), 7.46-7.41 (m, 2H), 6.99 (t, *J* = 7.5 Hz, 2H), 6.92 (d, *J* = 8.3 Hz, 2H), 3.67 (s, 6H) ppm. **$^{13}C\{^1H\}$ NMR** (151 MHz, $CDCl_3$) δ: 195.5, 158.5, 132.7, 130.6, 130.4, 120.5, 111.6, 55.9 ppm.
The NMR data are in accordance with those presented in literature.[25]

2,2',5,5'-Tetramethoxybenzophenone (3t)

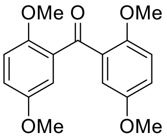

The title compound was prepared according to General procedure 1 from 1-bromo-2,5-dimethoxybenzene (868 mg, 601 µL, 4.0 mmol) and obtained as a beige solid after column chromatography (diethyl ether/*n*-pentane with traces of acetic acid 1:9 to 1:4 *v/v*), UV active, 300 mg (0.99 mmol, 50% yield). **^1H NMR** (600 MHz, $CDCl_3$) δ: 7.08 (d, *J* = 3.2 Hz, 2H), 6.98 (dd, *J* = 8.9, 3.2 Hz, 2H), 6.85 (d, *J* = 8.9 Hz, 2H), 3.79 (s, 6H), 3.60 (s, 6H) ppm. **$^{13}C\{^1H\}$ NMR** (151 MHz, $CDCl_3$) δ: 194.9, 153.5, 152.9, 131.0, 118.7, 114.7, 113.4, 56.7, 56.0 ppm.
The NMR data are in close accordance with those presented in literature.[26]

Biadamantane (4p)

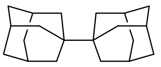

The title compound was isolated alongside **2m** as a colorless, crystalline solid, stains with CAM, 235 mg (0.87 mmol, 43% yield). **^1H NMR** (600 MHz, $CDCl_3$) δ: 1.95 (s, 3H), 1.63 (dd, *J* = 44.5, 11.0 Hz, 6H), 1.57 (d, *J* = 3.0 Hz, 6H) ppm. **$^{13}C\{^1H\}$ NMR** (151 MHz, $CDCl_3$) δ: 37.7, 36.5, 35.4, 29.2 ppm.
The NMR data are in close accordance with those presented in literature.[27]

References

[1] Mestrelab Research S.L., *MestReNova*. Version 12.0.1-20560, **2018**.
[2] G. R. Fulmer, A. J. M. Miller, N. H. Sherden, H. E. Gottlieb, A. Nudelman, B. M. Stoltz, J. E. Bercaw, K. I. Goldberg, *Organometallics* **2010**, *29*, 2176-2179.
[3] a) T. E. Hurst, J. A. Deichert, L. Kapeniak, R. Lee, J. Harris, P. G. Jessop, V. Snieckus, *Org. Lett.* **2019**, *21*, 3882-3885; b) D. Brillon, G. Sauve, *J. Org. Chem.* **1990**, *55*, 2246-2249.
[4] Occasionally, when flushing the vessel, some reaction mixture would move into the valve or even out of the vessel and thus be "lost". To prevent this, the vessel can be opened after step II, both valves can be flushed with CO_2 to remove the pasty reaction mixture from the holes of the lid, and the lid can then be shut again and flushed with CO_2 to remove any ambient air inside.
[5] J. M. Harrowfield, R. J. Hart, C. R. Whitaker, *Aust. J. Chem.* **2001**, *54*, 423-425.
[6] I. R. Speight, T. P. Hanusa, *Molecules* **2020**, *25*, 570.
[7] A. Krasovskiy, P. Knochel, *Angew. Chem. Int. Ed.* **2004**, *43*, 3333-3336; *Angew. Chem.* **2004**, *116*, 3396-3399.
[8] C. Villiers, J.-P. Dognon, R. Pollet, P. Thuéry, M. Ephritikhine, *Angew. Chem. Int. Ed.* **2010**, *49*, 3465-3468; *Angew. Chem.* **2010**, *122*, 3543-3546.
[9] I. R. Speight, T. P. Hanusa, *Molecules* **2020**, *25*, 570.
[10] a) F. Schneider, T. Szuppa, A. Stolle, B. Ondruschka, H. Hopf, *Green Chem.* **2009**, *11*, 1894-1899; b) F. Schneider, B. Ondruschka, *ChemSusChem* **2008**, *1*, 622-625 and references therein.
[11] S. Murarka, J. Möbus, G. Erker, C. Mück-Lichtenfeld, A. Studer, *Org. Biomol. Chem.* **2015**, *13*, 2762-2767.#
[12] O. García Mancheño, T. Stopka, *Synthesis* **2013**, *45*, 1602-1611.
[13] a) R. Levine, M. J. Karten, W. M. Kadunce, *J. Org. Chem.* **1975**, *40*, 1770; b) G. Zadel, E. Breitmaier, *Angew. Chem.* **1992**, *104*, 1070-1071; *Angew. Chem. Int. Ed.* **1992**, *31*, 1035–1036; c) J. Wu, X. Yang, Z. He, X. Mao, T. A. Hatton, T. F. Jamison, *Angew. Chem.* **2014**, *126*, 8556-8560; *Angew. Chem. Int. Ed.* **2014**, *53*, 8416-8420.
[14] a) S. Nahm, S. M. Weinreb, *Tetrahedron Lett.* **1981**, *22*, 3815-3818; b) T. Fujisawa, T. Mori, T. Sato, *Tetrahedron Lett.* **1982**, *23*, 5059-5062; c) V. Fiandanese, G. Marchese, L. Ronzini, *Tetrahedron Lett.* **1983**, *24*, 3677-3680; d) L. Garnier, Y. Rollin, J. Périchon, *J. Organomet. Chem.* **1989**, *367*, 347-358; e) K. Colas, A. C. V. D. Dos Santos, A. Mendoza, *Org. Lett.* **2019**, *21*, 7908-7913.
[15] K. L. Denlinger, P. Carr, D. C. Waddell, J. Mack, *Molecules* **2020**, *25*, 364.
[16] A. Correa, R. Martín, *J. Am. Chem. Soc.* **2009**, *131*, 15974-15975.
[17] J. M. Valera Lauridsen, S. Y. Cho, H. Y. Bae, J.-W. Lee, *Organometallics* **2020**, *39*, 1652-1657.
[18] N. Tada, K. Hattori, T. Nobuta, T. Miura, A. Itoh, *Green Chem.* **2011**, *13*, 1669-1671.
[19] W. Osim, A. Stojanovic, J. Akbarzadeh, H. Peterlik, W. H. Binder, *Chem. Commun.* **2013**, *49*, 9311-9313#.
[20] K. Durka, S. Luliński, M. Dąbrowski, J. Serwatowski, *Eur. J. Org. Chem.* **2014**, 4562-4570.
[21] P. Beak, L. G. Carter, *J. Org. Chem.* **1981**, *46*, 2363-2373.
[22] S. Tang, M. Rauch, M. Montag, Y. Diskin-Posner, Y. Ben-David, D. Milstein, *J. Am. Chem. Soc.* **2020**, *142*, 20875-20882.
[23] G. Urgoitia, R. SanMartin, M. T. Herrero, E. Domínguez, *Adv. Synth. Catal.* **2016**, *358*, 1150-1156.
[24] M. Börjesson, T. Moragas, R. Martin, *J. Am. Chem. Soc.* **2016**, *138*, 7504-7507.
[25] A. Rérat, C. Michon, F. Agbossou-Niedercorn, C. Gosmini, *Eur. J. Org. Chem.* **2016**, 4554-4560.
[26] C. Kuhakarn, N. Surapanich, S. Kamtonwong, M. Pohmakotr, V. Reutrakul, *Eur. J. Org. Chem.* **2011**, 5911-5918.
[27] T. Nishino, T. Watanabe, M. Okada, Y. Nishiyama, N. Sonoda, *J. Org. Chem.* **2002**, *67*, 966-969.

Author contributions

V.P. conducted initial experiments with CO_2 and SMC, designed the study, investigated the scope of bromides, analyzed the results, and wrote the original draft. R.V. conducted the optimization experiments with CO_2 and contributed to the scope with SMC. J.N. conducted the optimization experiments and investigated the scope with SMC. C.B. led the study, provided the idea and scientific insights, acquired the funding, and revised the drafts.

Appendix: ^{1}H, ^{13}C{^{1}H} and ^{19}F NMR spectra

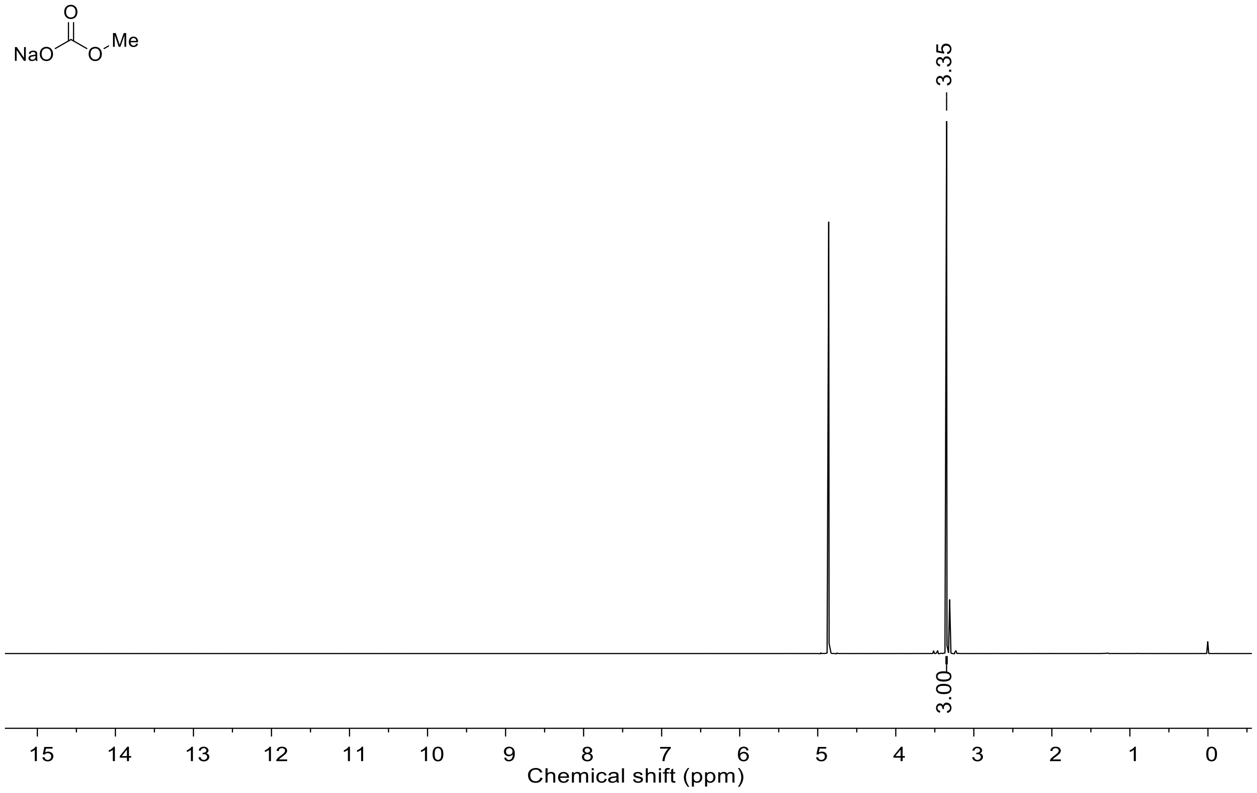

Figure F1. ^{1}H NMR spectrum of sodium methyl carbonate (methanol-d$_4$, 600 MHz)

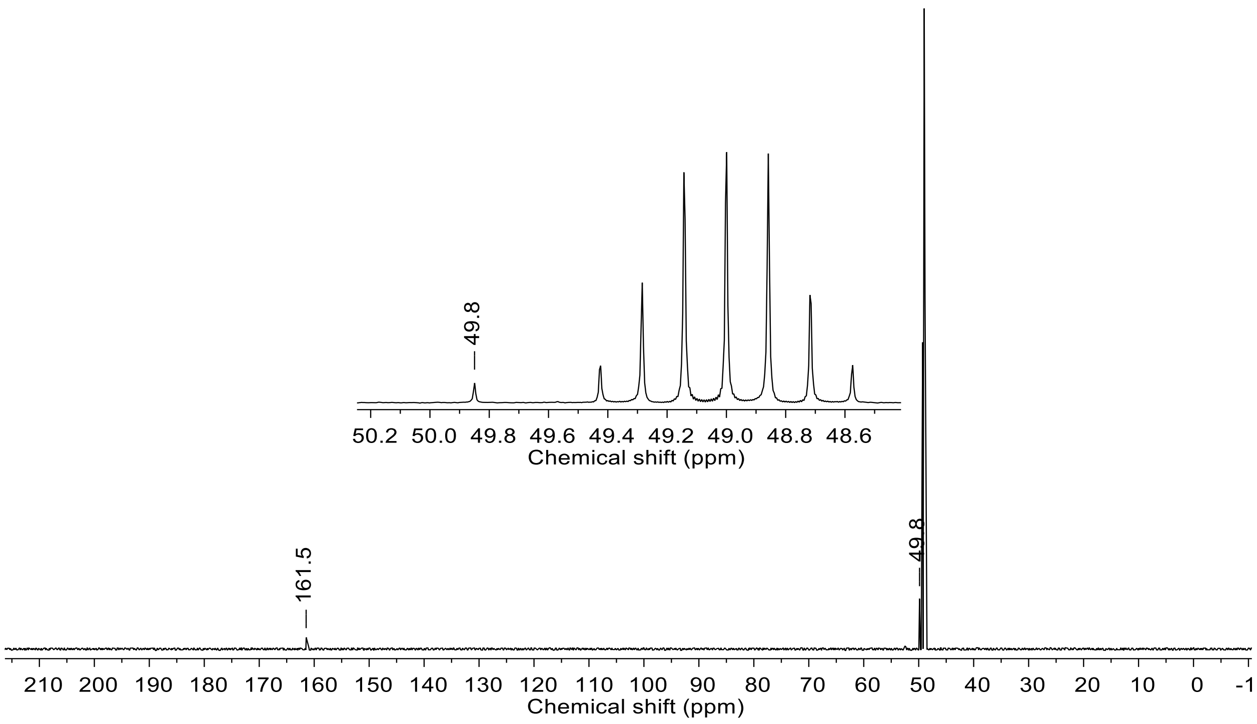

Figure F2. ^{13}C{^{1}H} NMR spectrum of sodium methyl carbonate (methanol-d$_4$, 151 MHz).

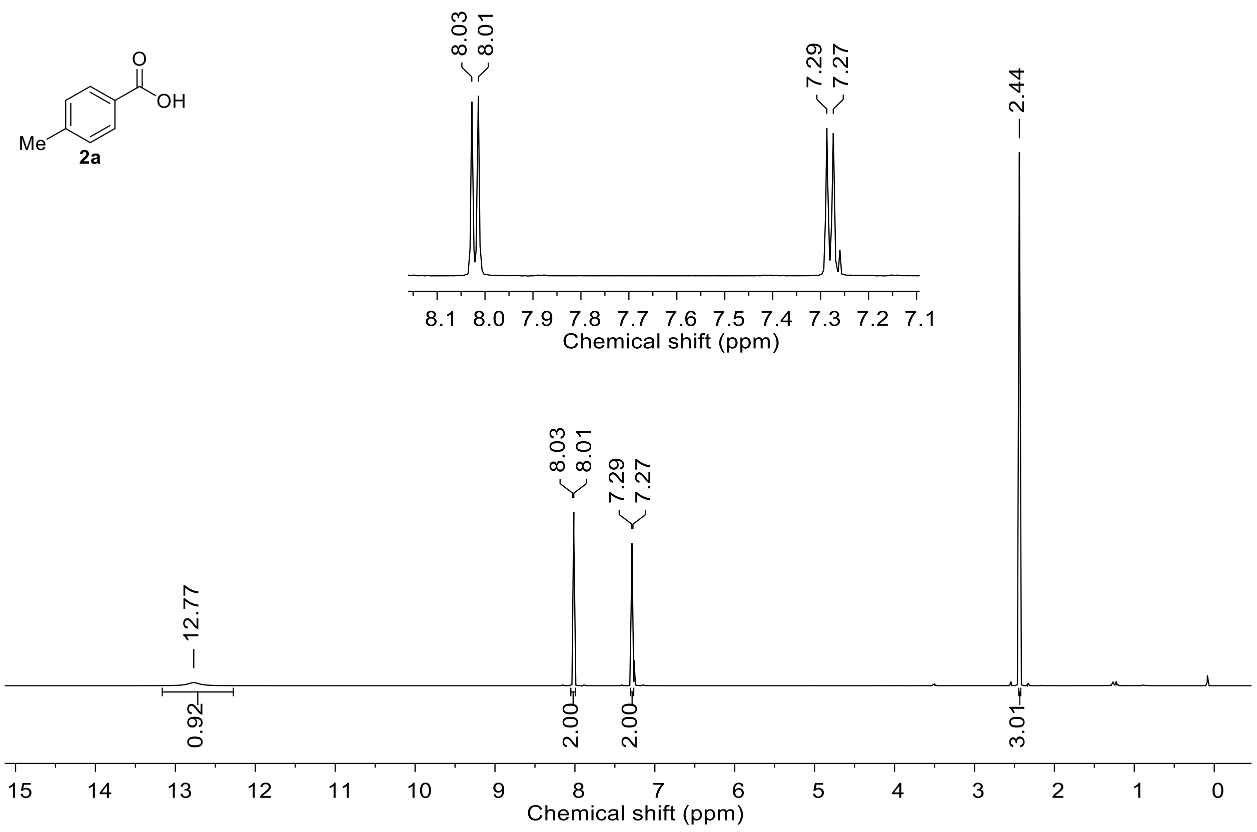

Figure F3. ^{1}H NMR spectrum of 4-methylbenzoic acid **2a** ($CDCl_3$, 600 MHz).

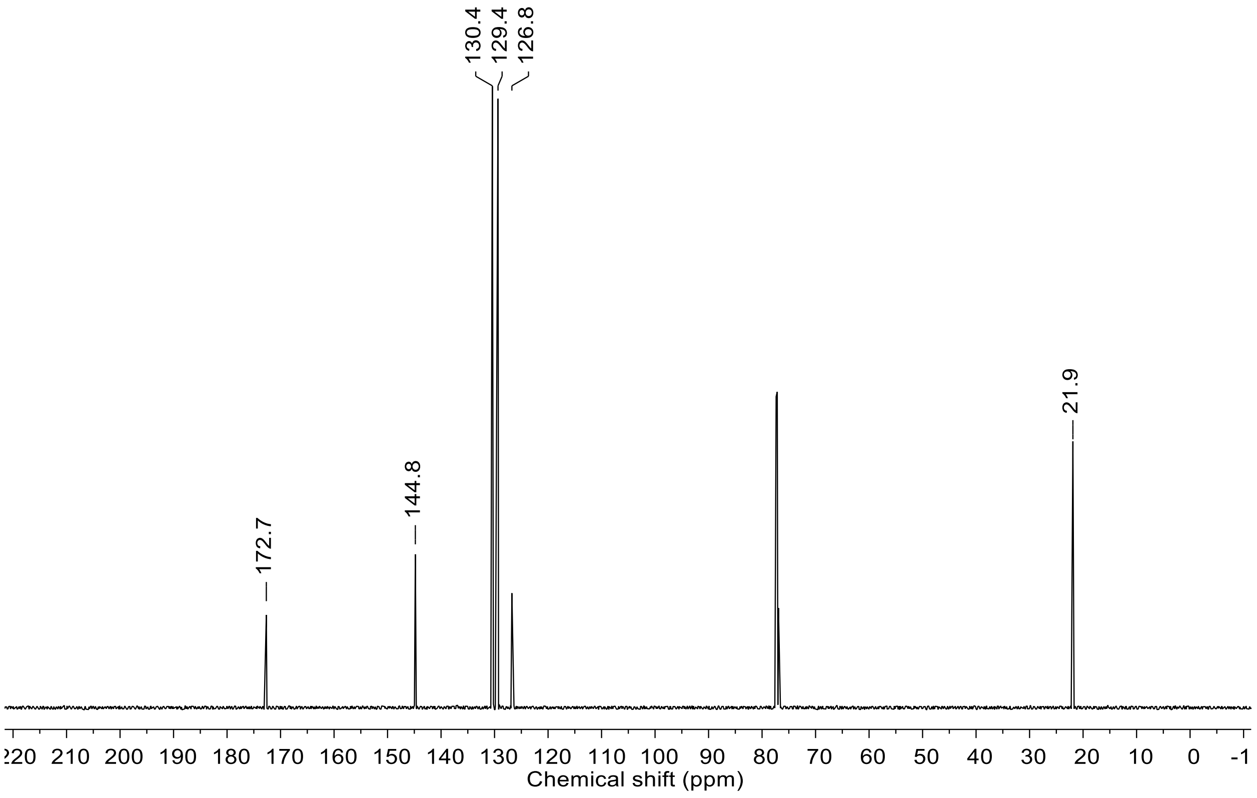

Figure F4. $^{13}C\{^{1}H\}$ NMR spectrum of 4-methylbenzoic acid **2a** ($CDCl_3$, 151 MHz).

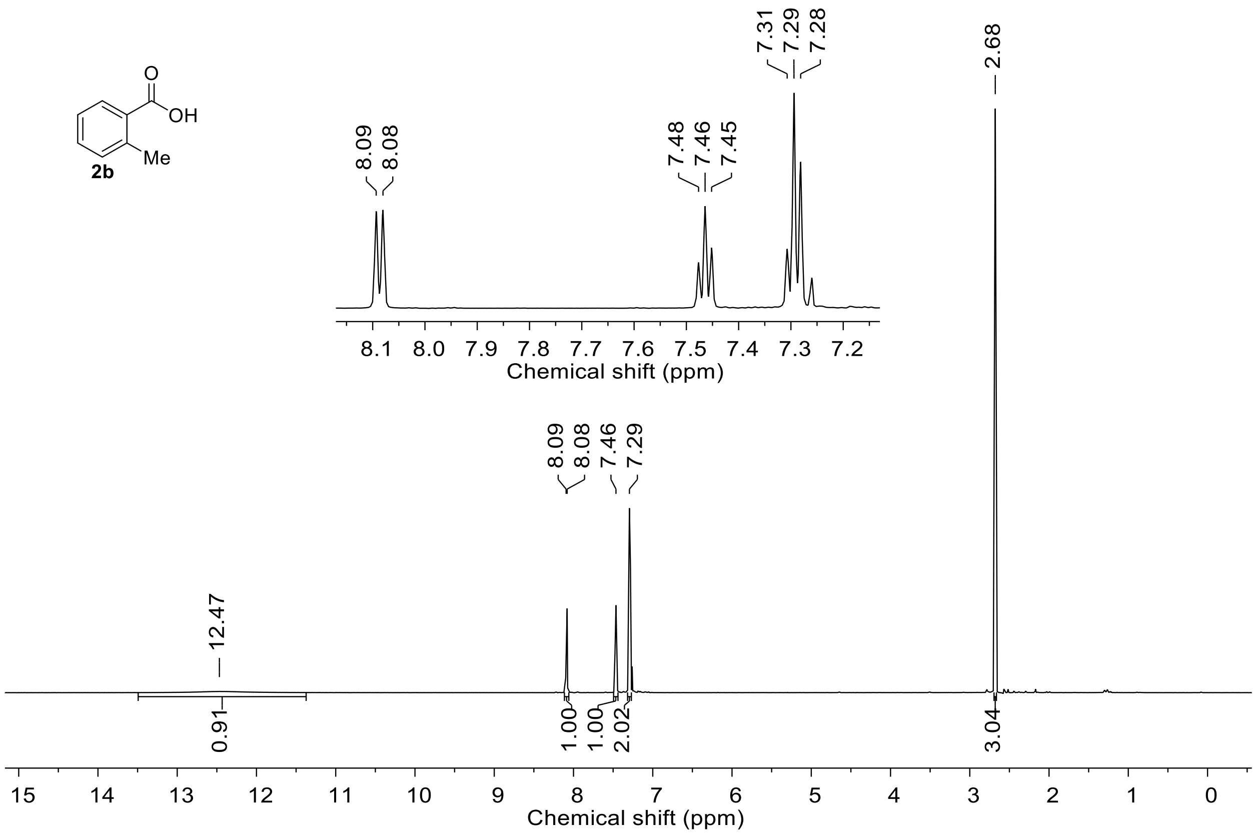

Figure F5. ^{1}H NMR spectrum of 2-methylbenzoic acid **2b** ($CDCl_3$, 600 MHz).

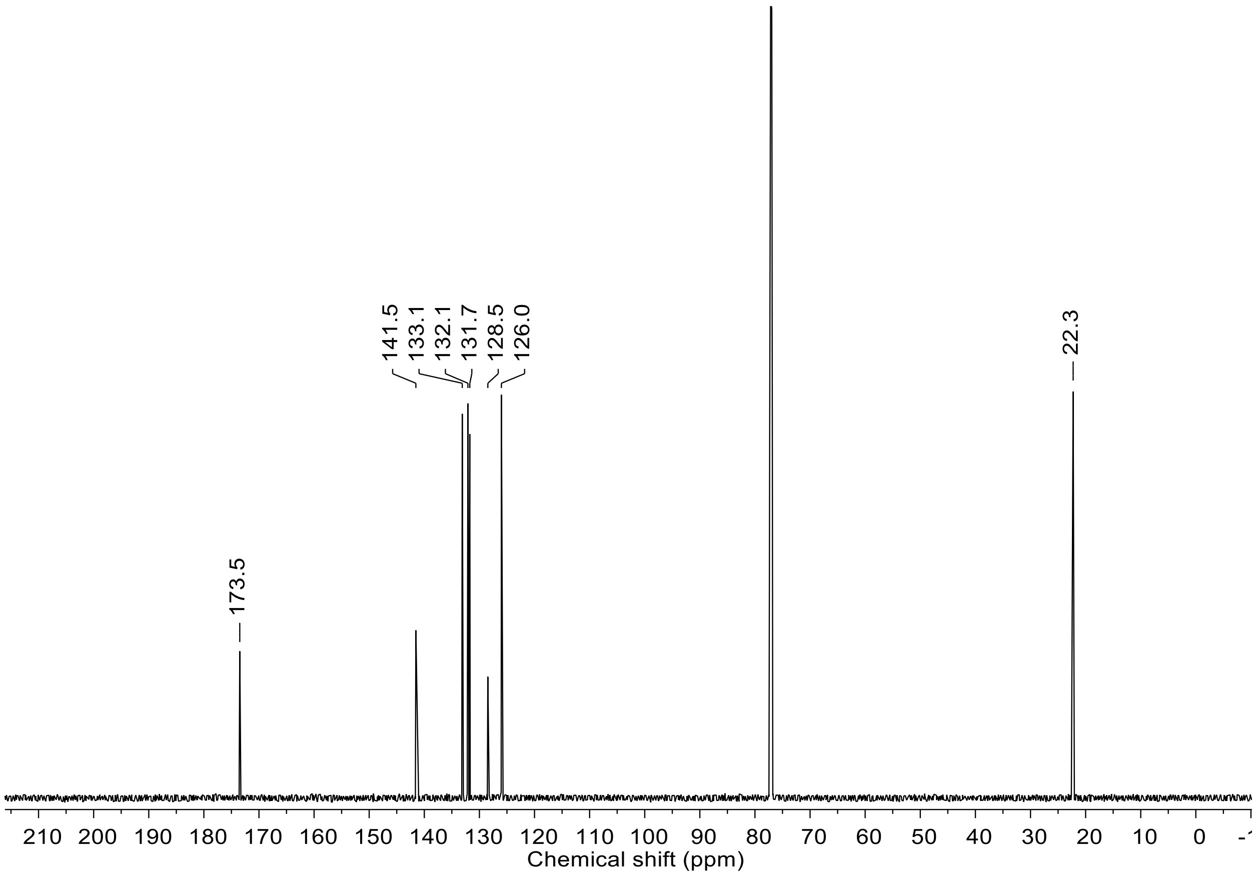

Figure F6. $^{13}C\{^{1}H\}$ NMR spectrum of 2-methylbenzoic acid **2b** ($CDCl_3$, 151 MHz).

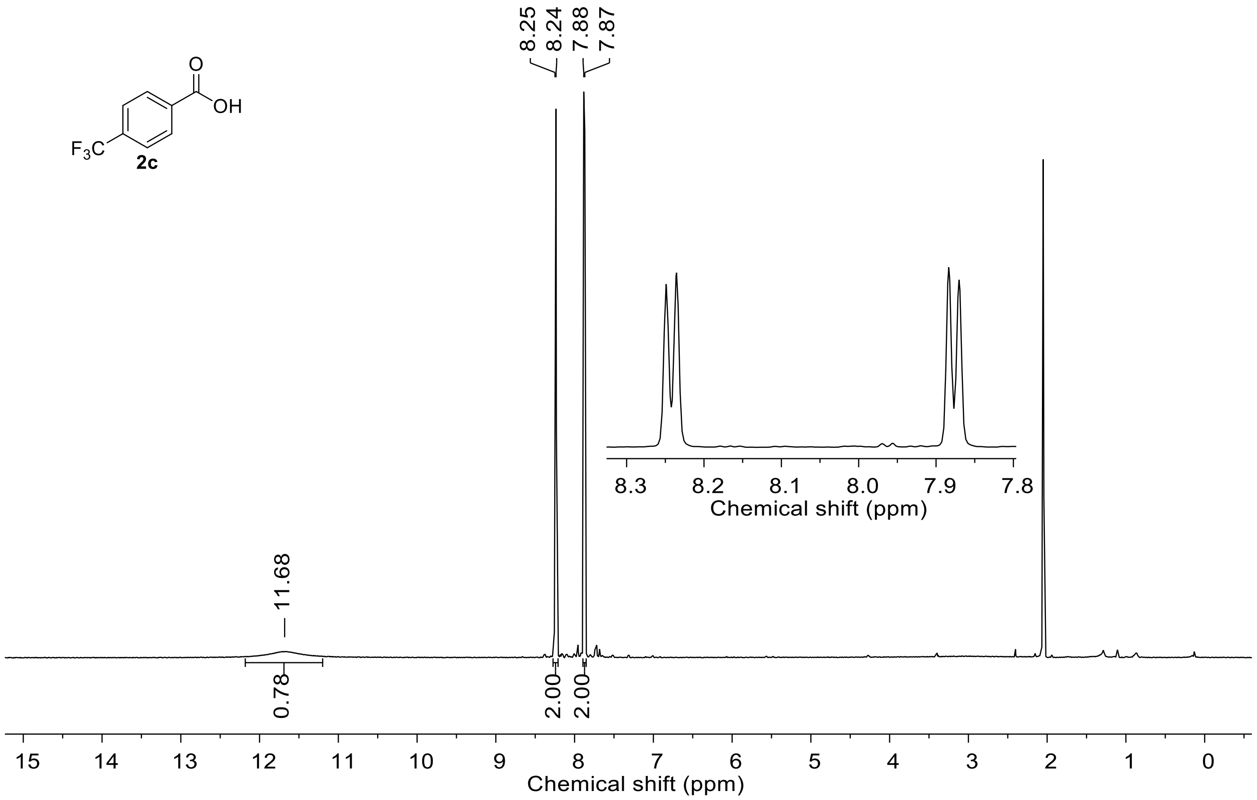

Figure F7. ^{1}H NMR spectrum of 4-(trifluoromethyl)benzoic acid **2c** (acetone-d_6, 600 MHz).

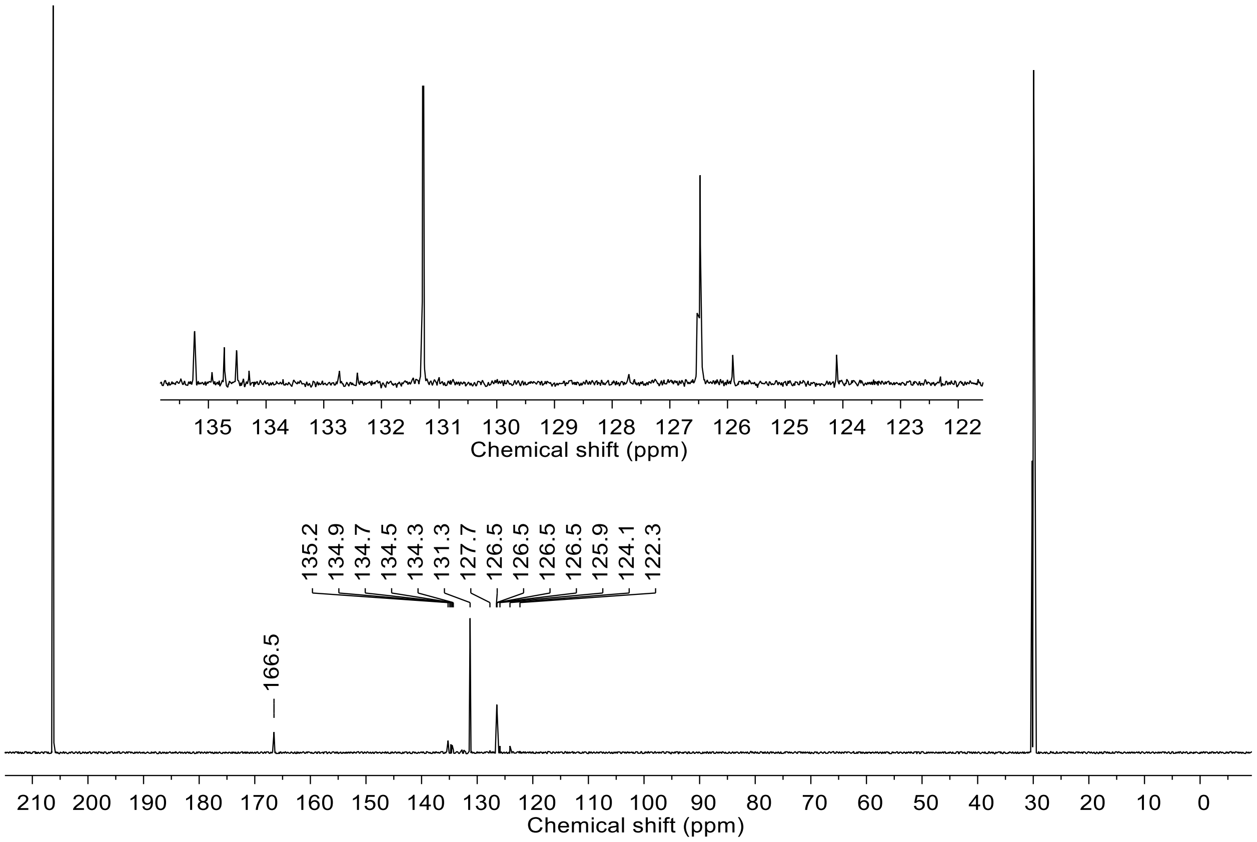

Figure F8. $^{13}C\{^{1}H\}$ NMR spectrum of 4-(trifluoromethyl)benzoic acid **2c** (acetone-d_6, 151 MHz).

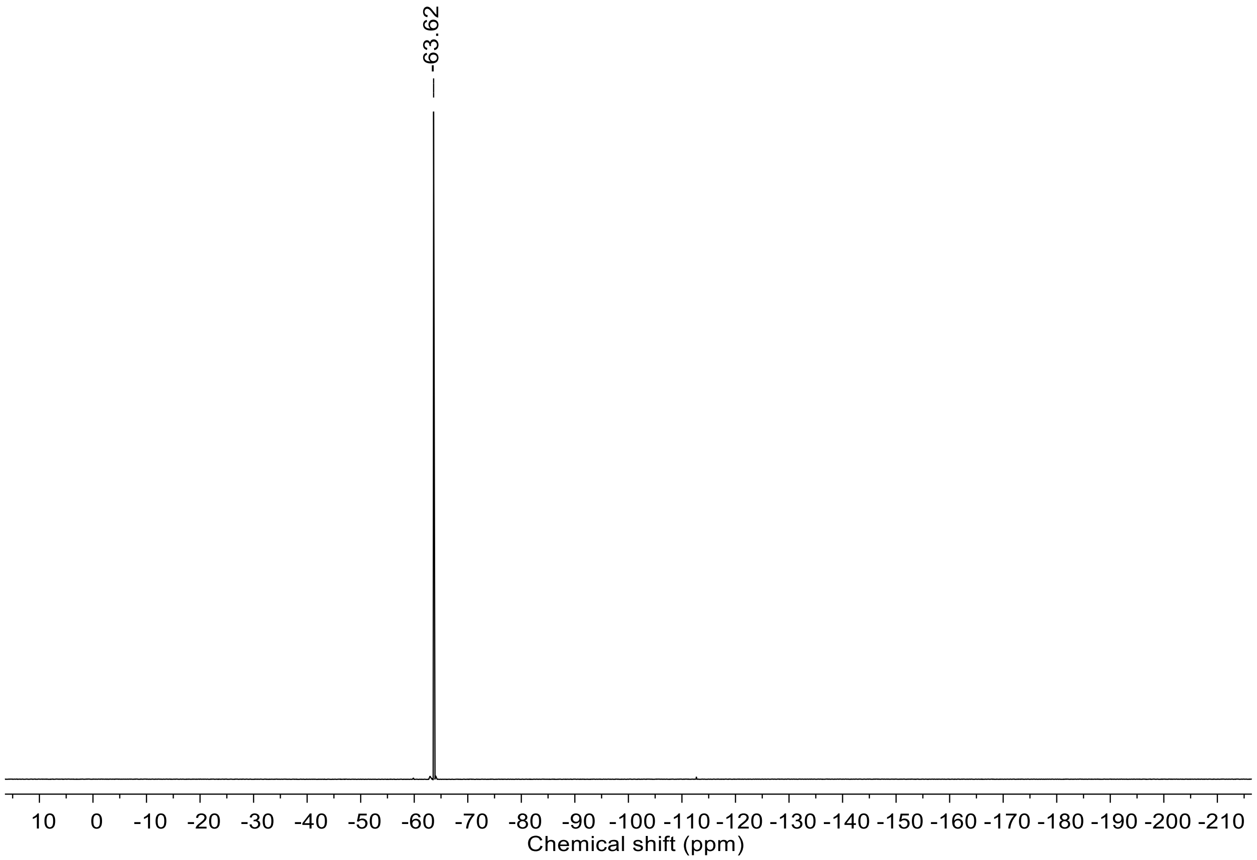

Figure F9. ^{19}F NMR spectrum of 4-(trifluoromethyl)benzoic acid **2c** (acetone-d_6, 565 MHz).

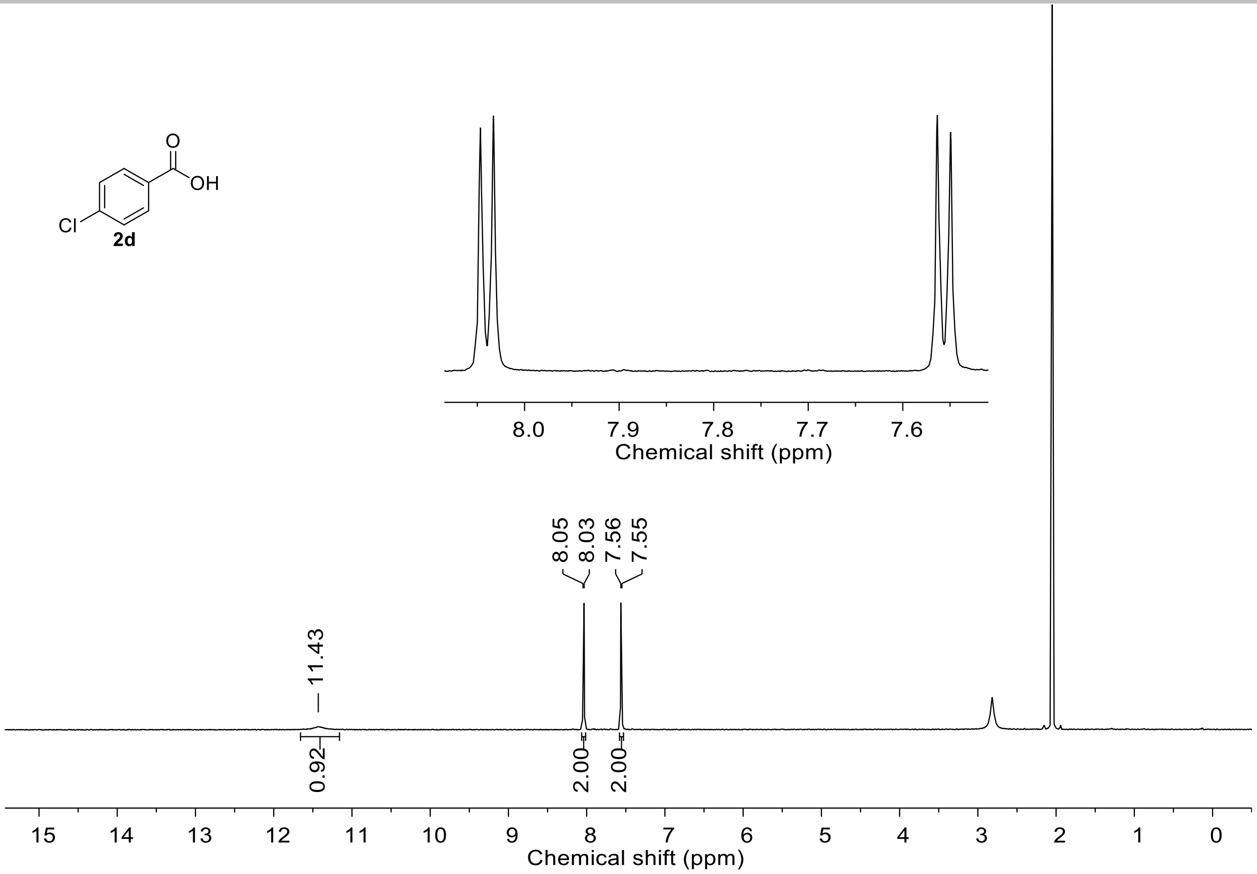

Figure F10. ^{1}H NMR spectrum of 4-chlorobenzoic acid **2d** (acetone-d_6, 600 MHz).

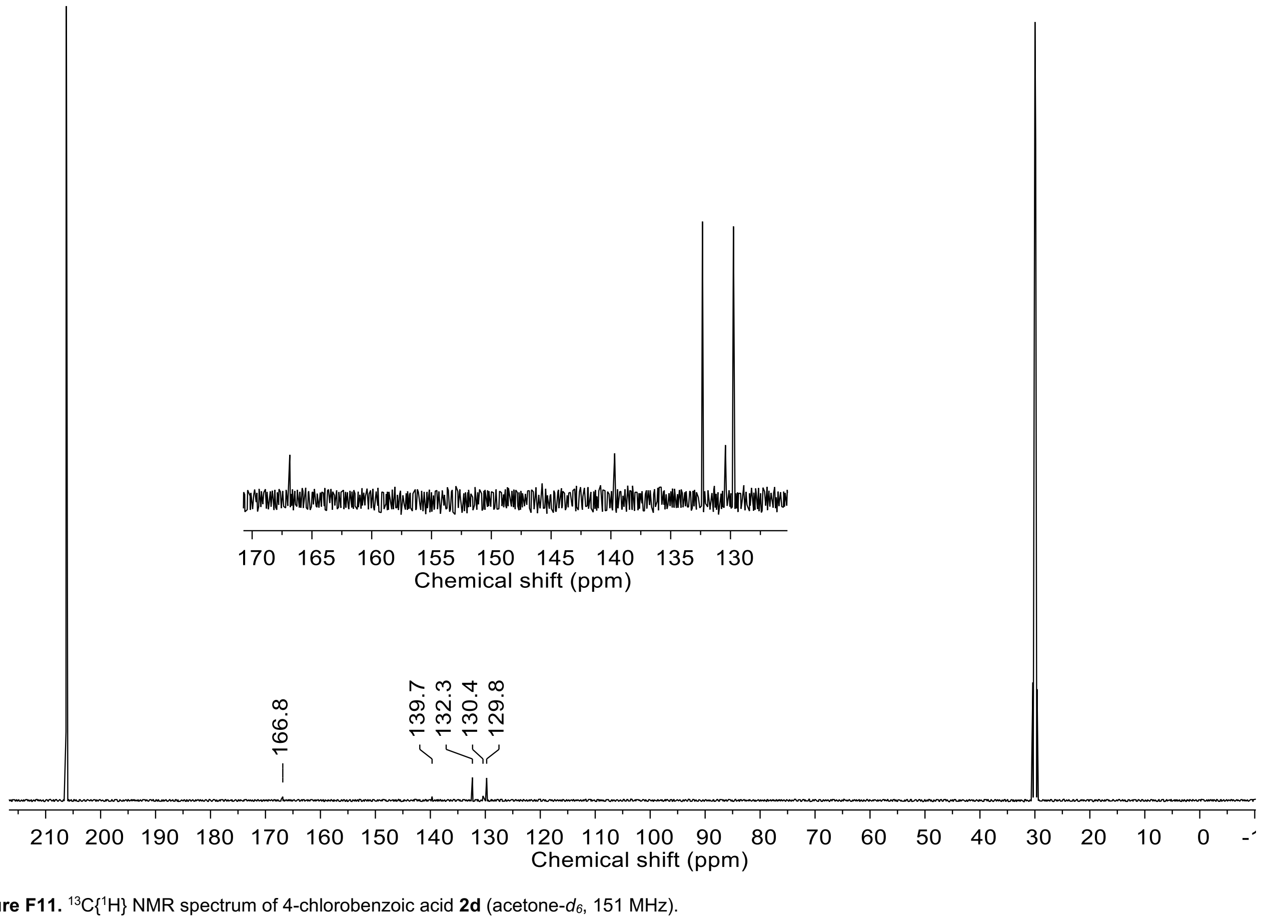

Figure F11. $^{13}C\{^{1}H\}$ NMR spectrum of 4-chlorobenzoic acid **2d** (acetone-d_6, 151 MHz).

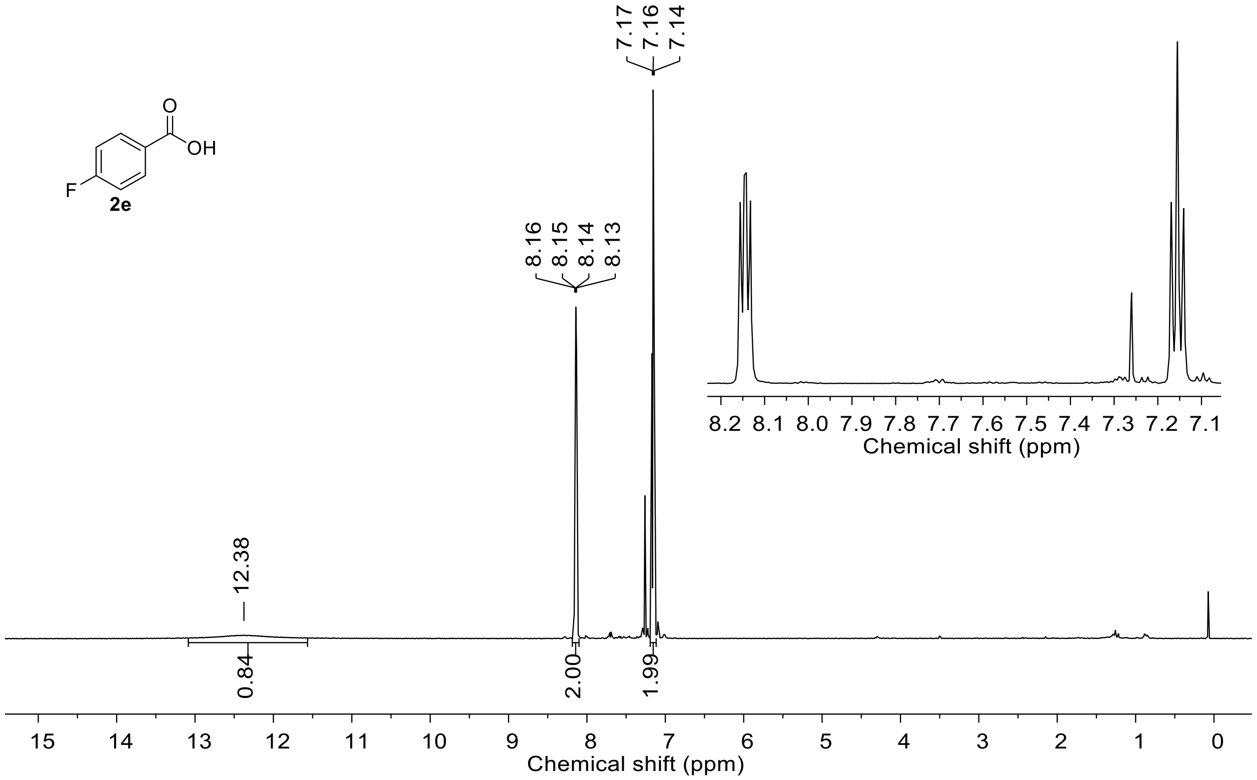

Figure F12. ^{1}H NMR spectrum of 4-fluorobenzoic acid **2e** ($CDCl_3$, 600 MHz).

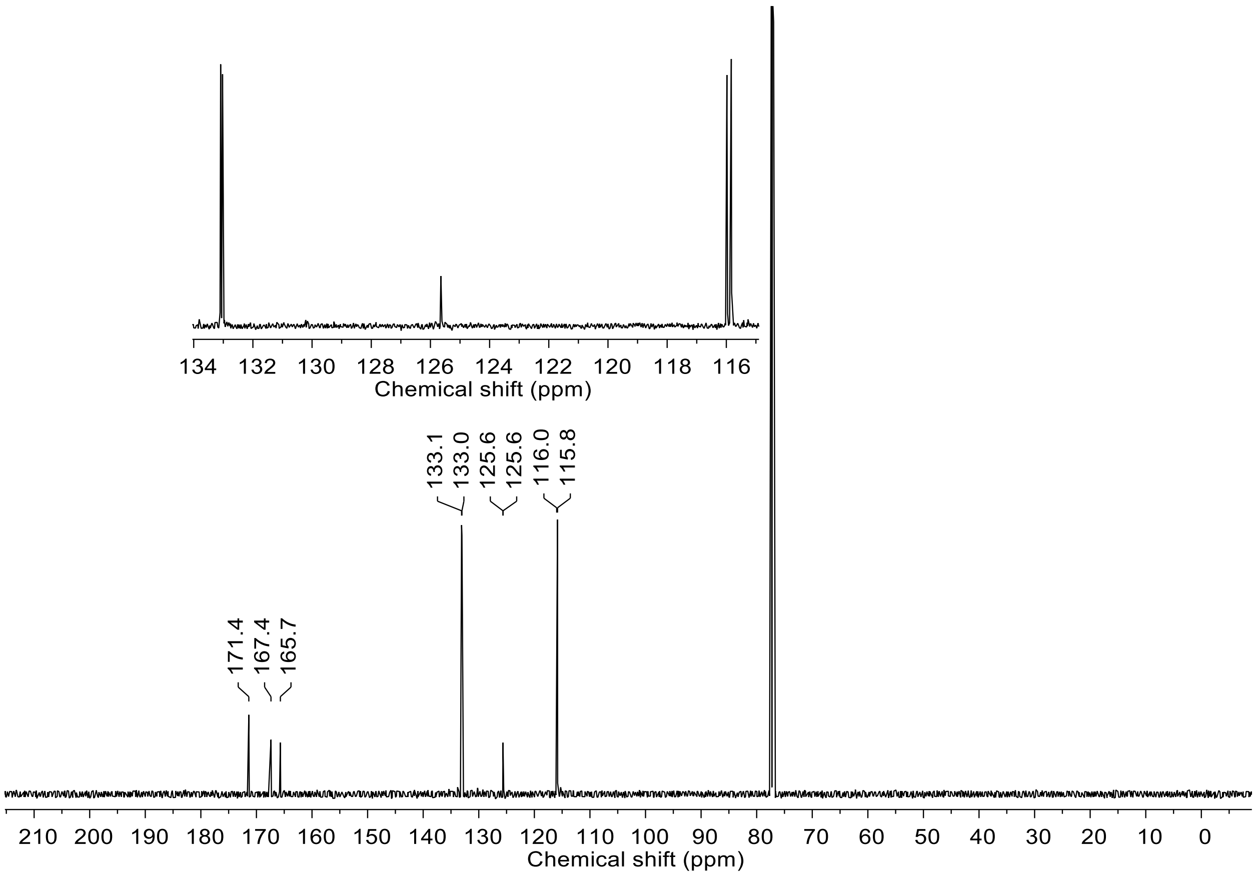

Figure F13. $^{13}C\{^{1}H\}$ NMR spectrum of 4-fluorobenzoic acid **2e** ($CDCl_3$, 151 MHz).

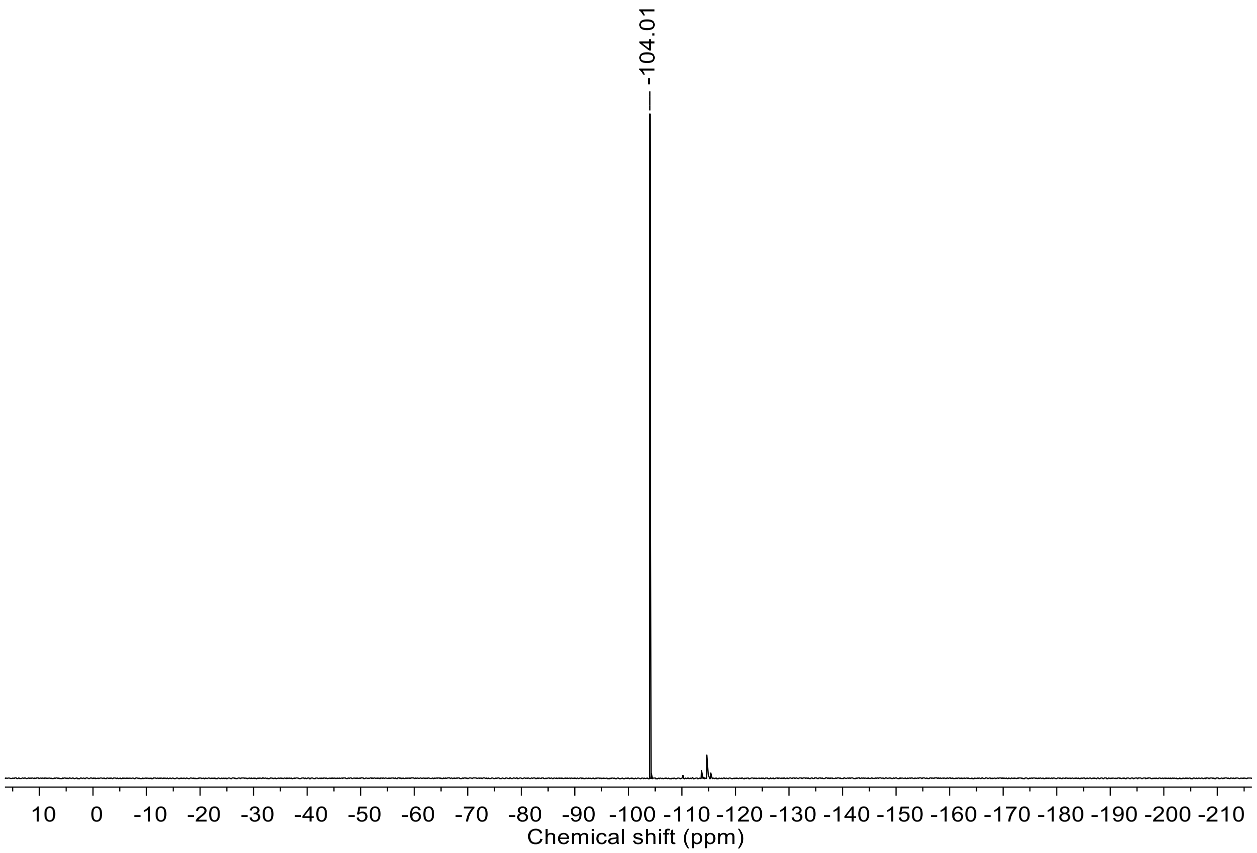

Figure F14. ^{19}F NMR spectrum of 4-fluorobenzoic acid **2e** ($CDCl_3$, 565 MHz).

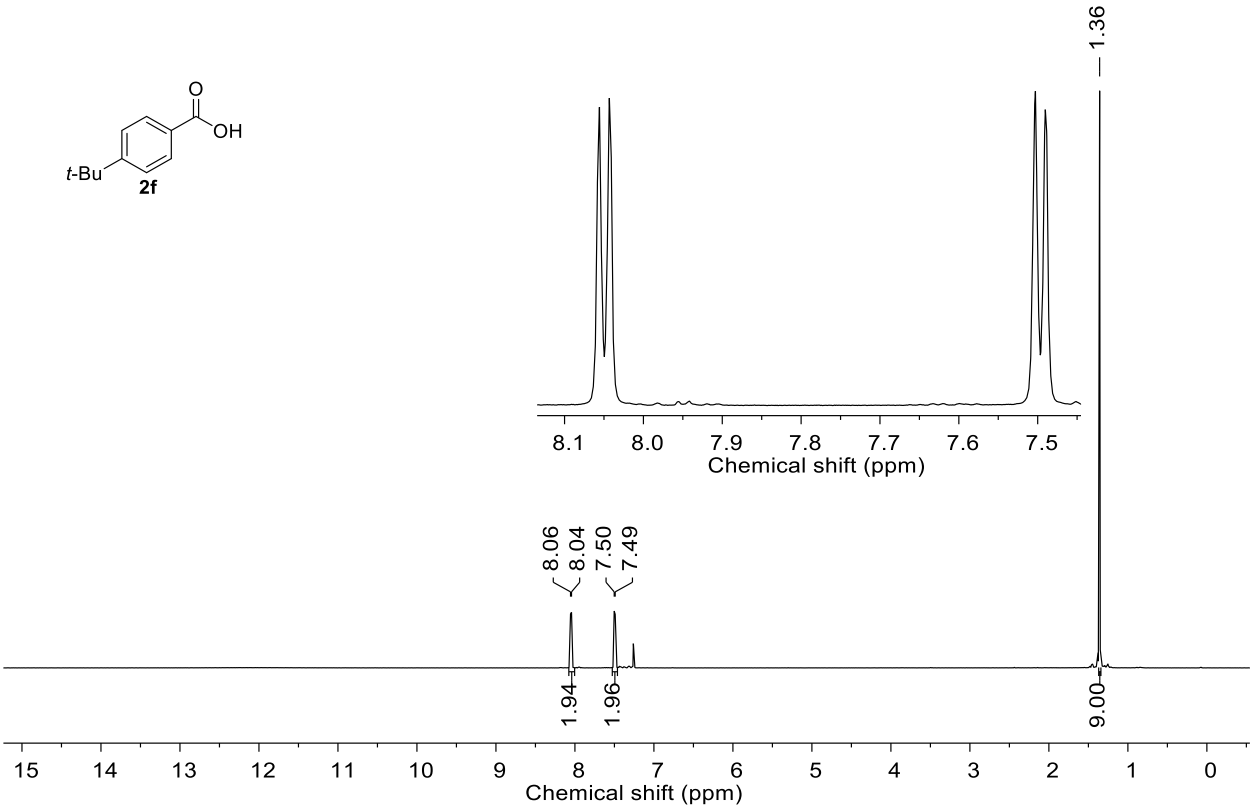

Figure F15. ^{1}H NMR spectrum of 4-*tert*-butylbenzoic acid **2f** ($CDCl_3$, 600 MHz).

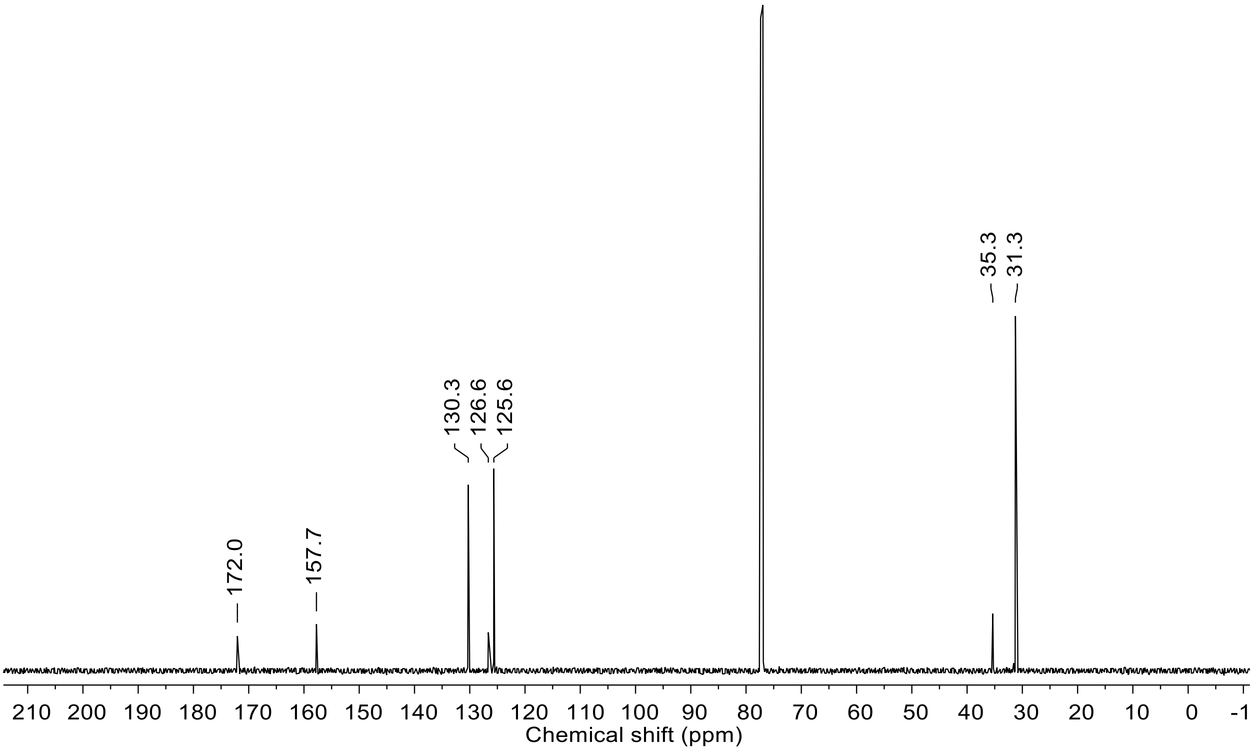

Figure F16. $^{13}C\{^{1}H\}$ NMR spectrum of 4-*tert*-butylbenzoic acid **2f** ($CDCl_3$, 151 MHz).

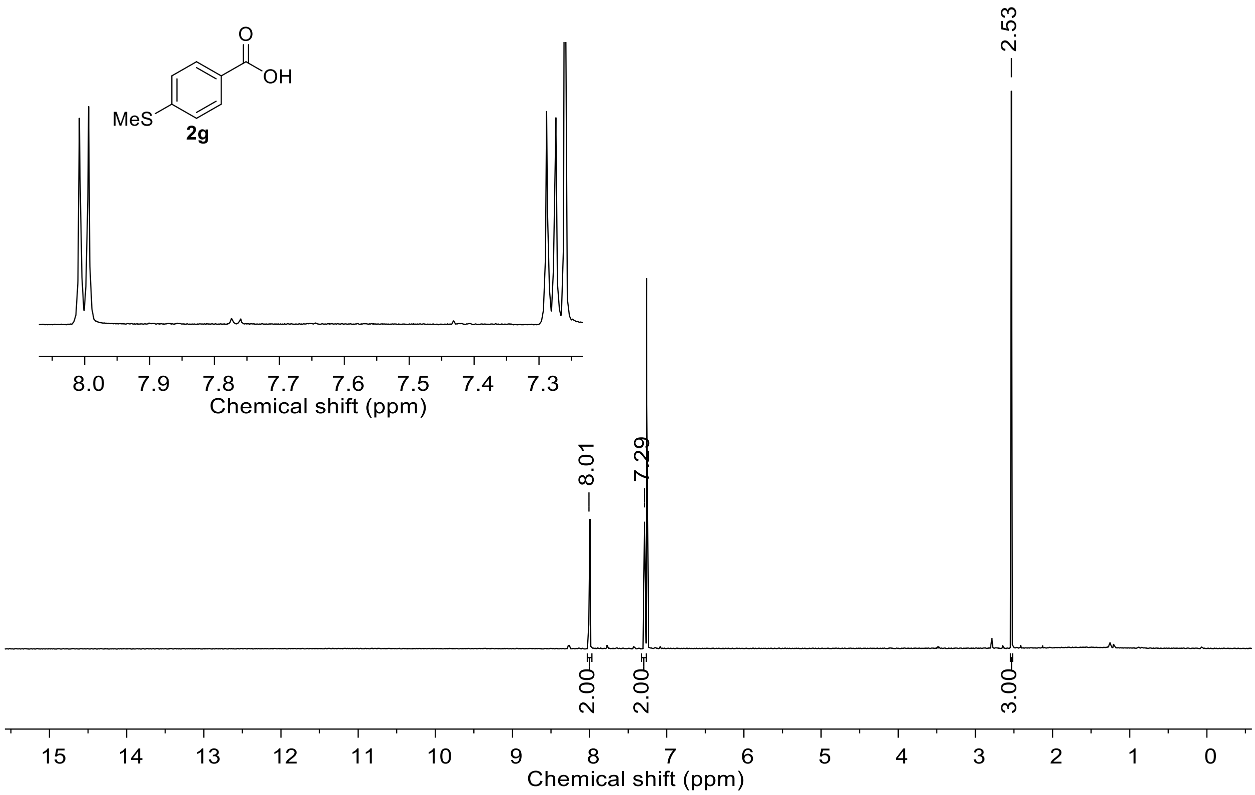

Figure F17. ^{1}H NMR spectrum of 4-Thiomethylbenzoic acid **2g** ($CDCl_3$, 600 MHz).

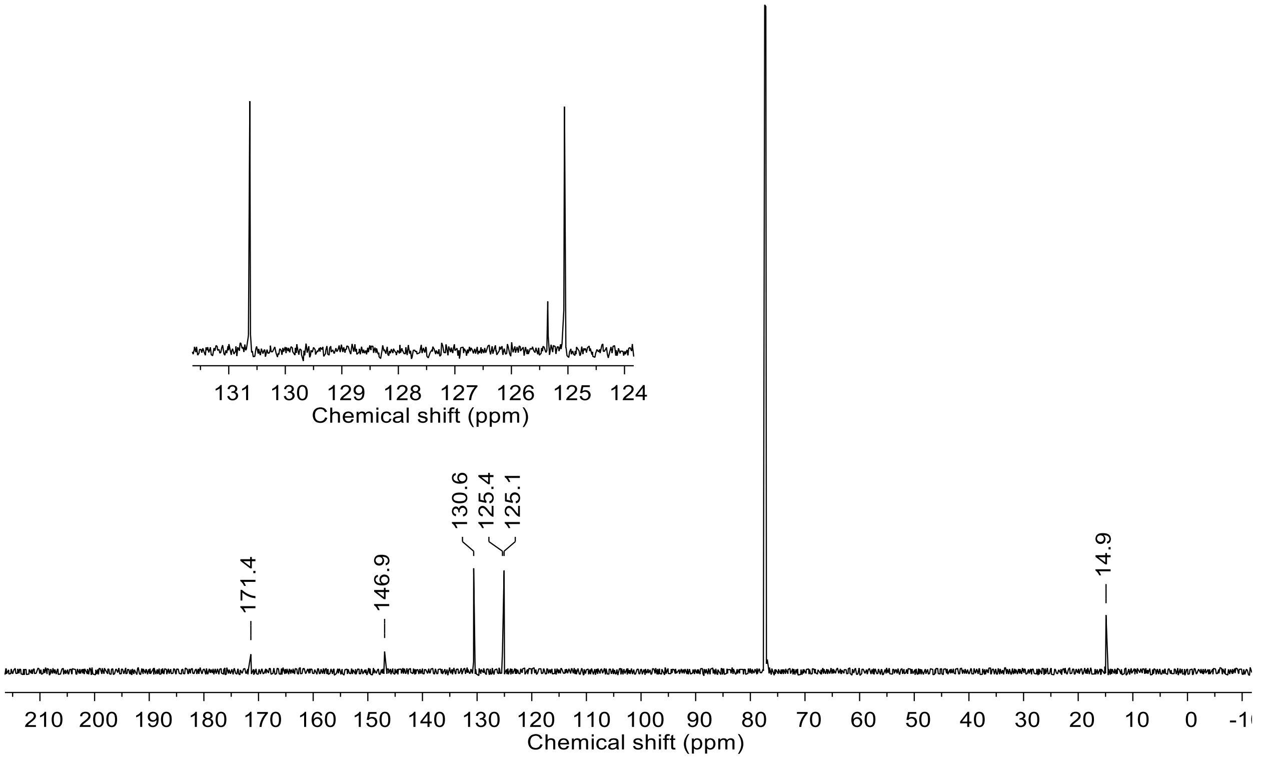

Figure F18. $^{13}C\{^{1}H\}$ NMR spectrum of 4-Thiomethylbenzoic acid **2g** ($CDCl_3$, 151 MHz).

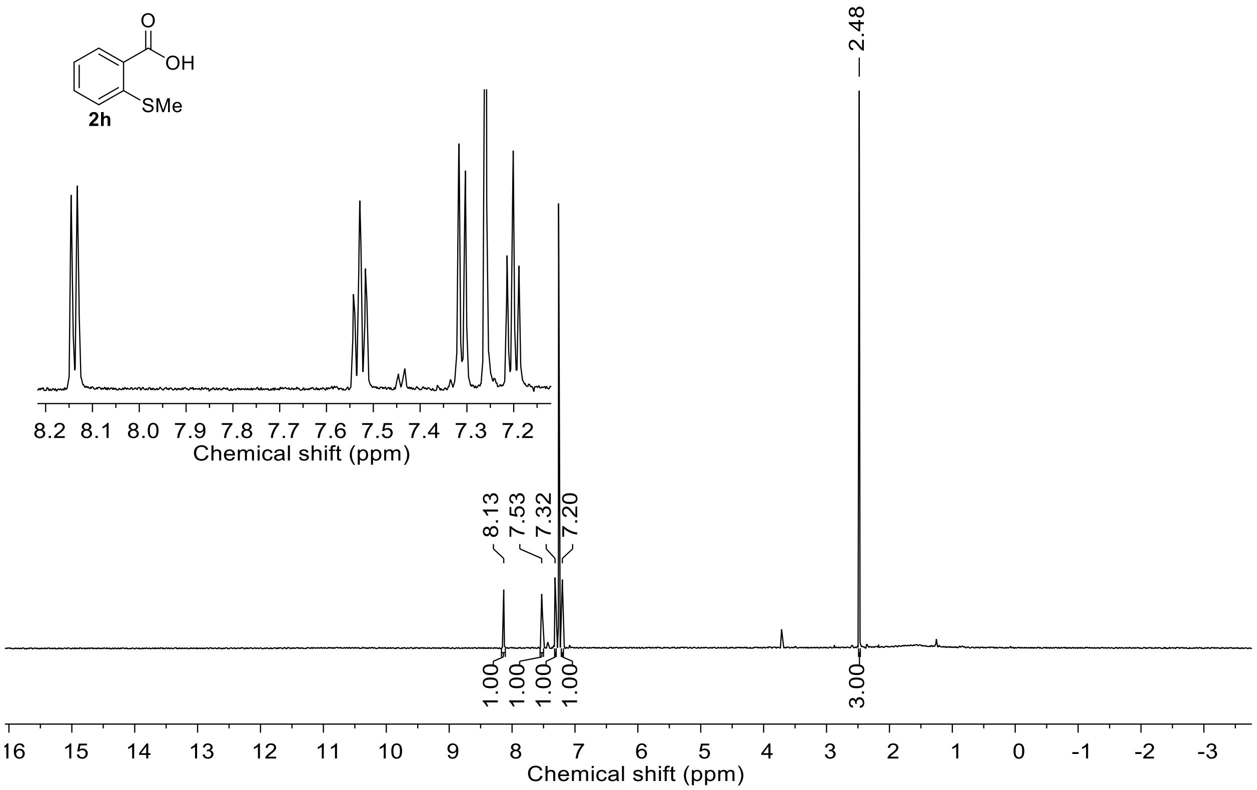

Figure F19. ^{1}H NMR spectrum of 2-Thiomethylbenzoic acid **2h** ($CDCl_3$, 600 MHz).

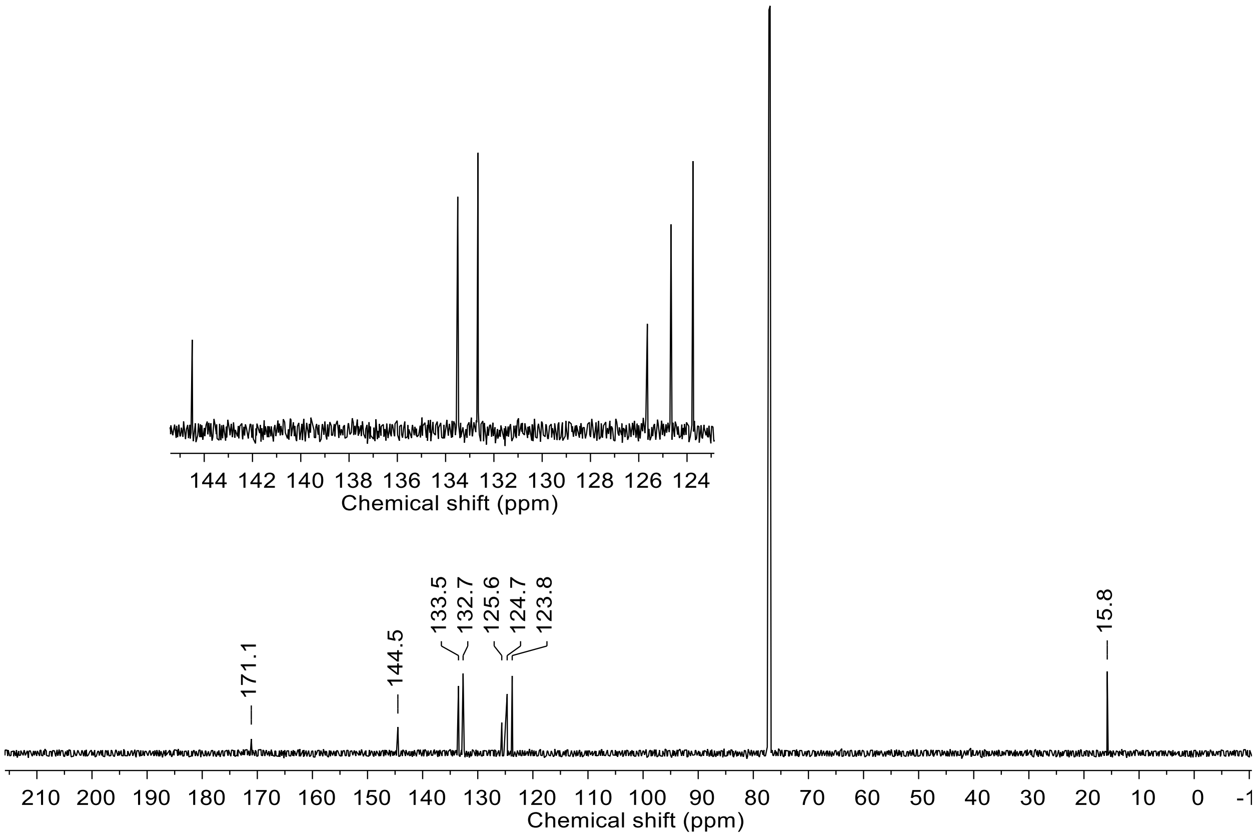

Figure F20. $^{13}C\{^{1}H\}$ NMR spectrum of 2-Thiomethylbenzoic acid **2h** ($CDCl_3$, 151 MHz).

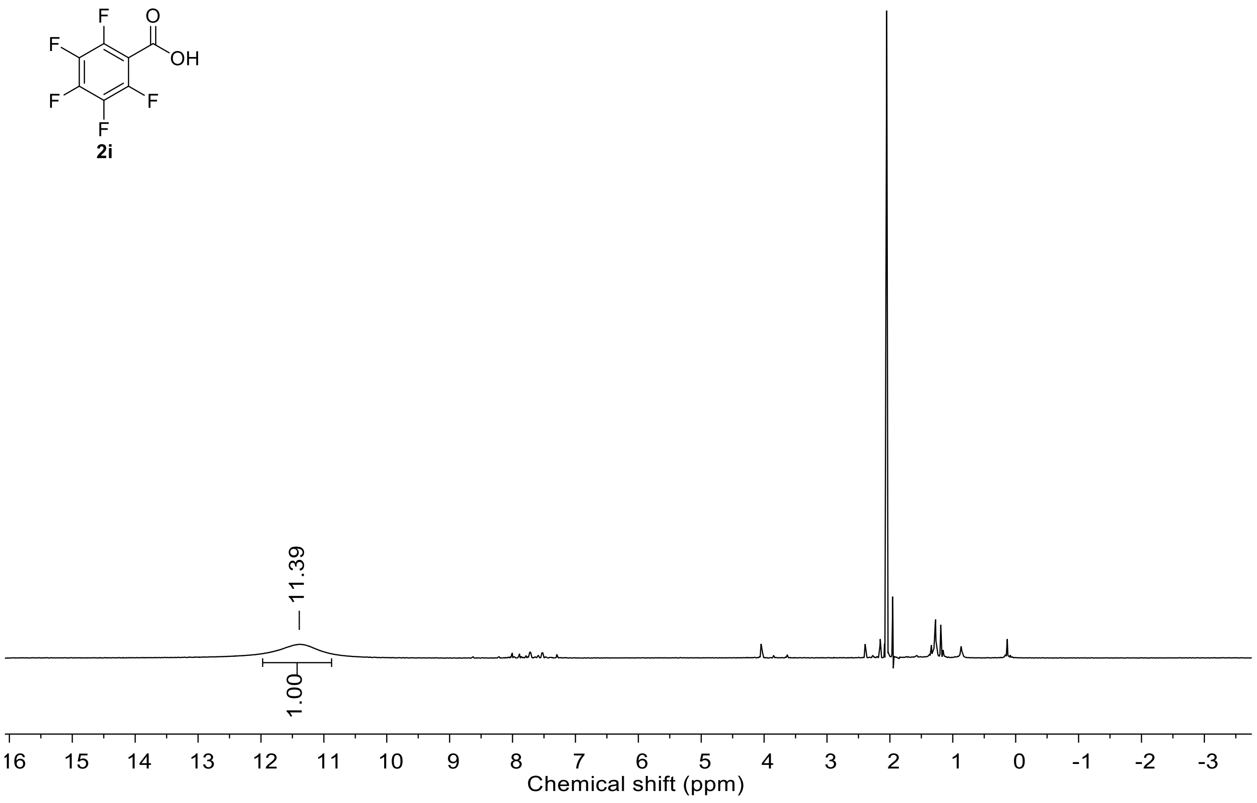

Figure F21. ^{1}H NMR spectrum of 2,3,4,5,6-pentafluorobenzoic acid **2i** (acetone-d_6, 600 MHz).

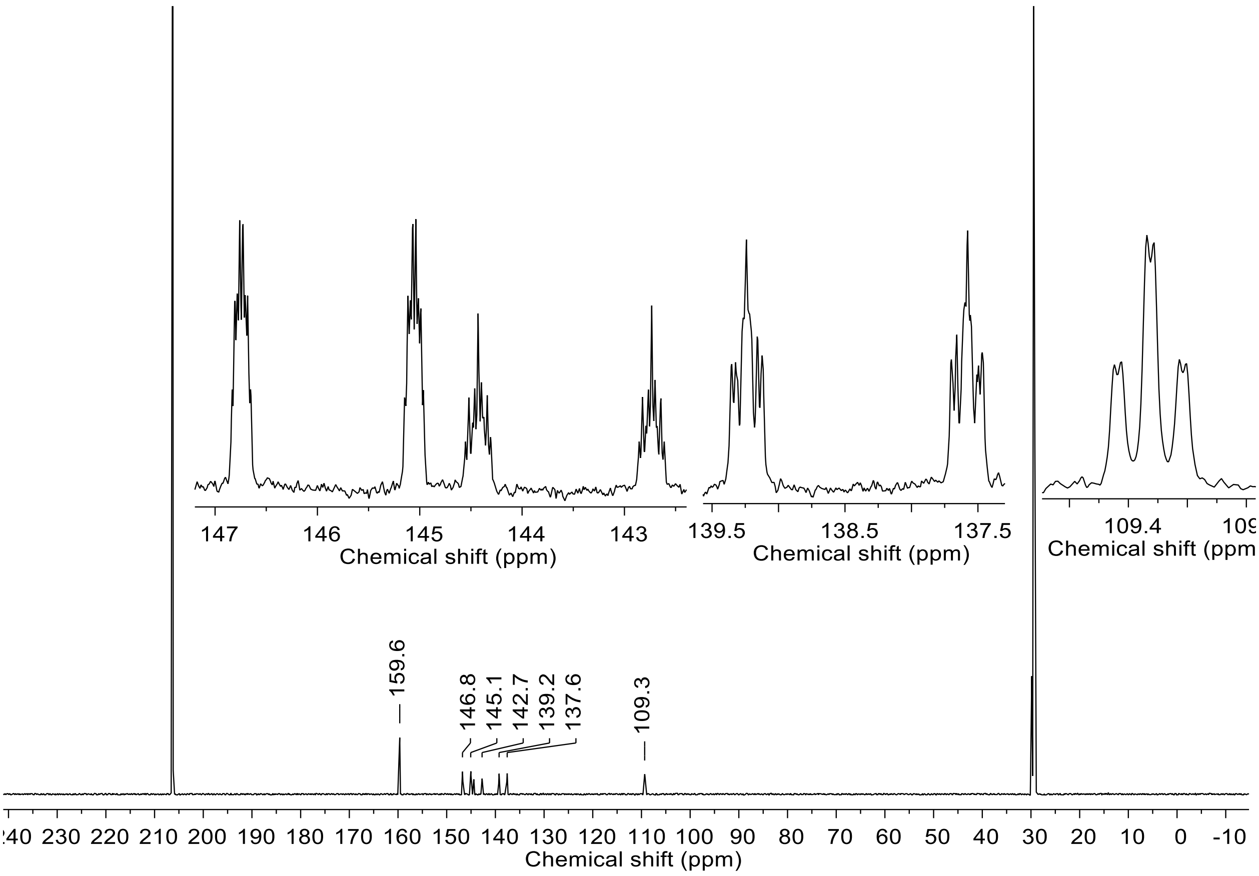

Figure F22. $^{13}C\{^{1}H\}$ NMR spectrum of 2,3,4,5,6-pentafluorobenzoic acid **2i** (acetone-d_6, 151 MHz).

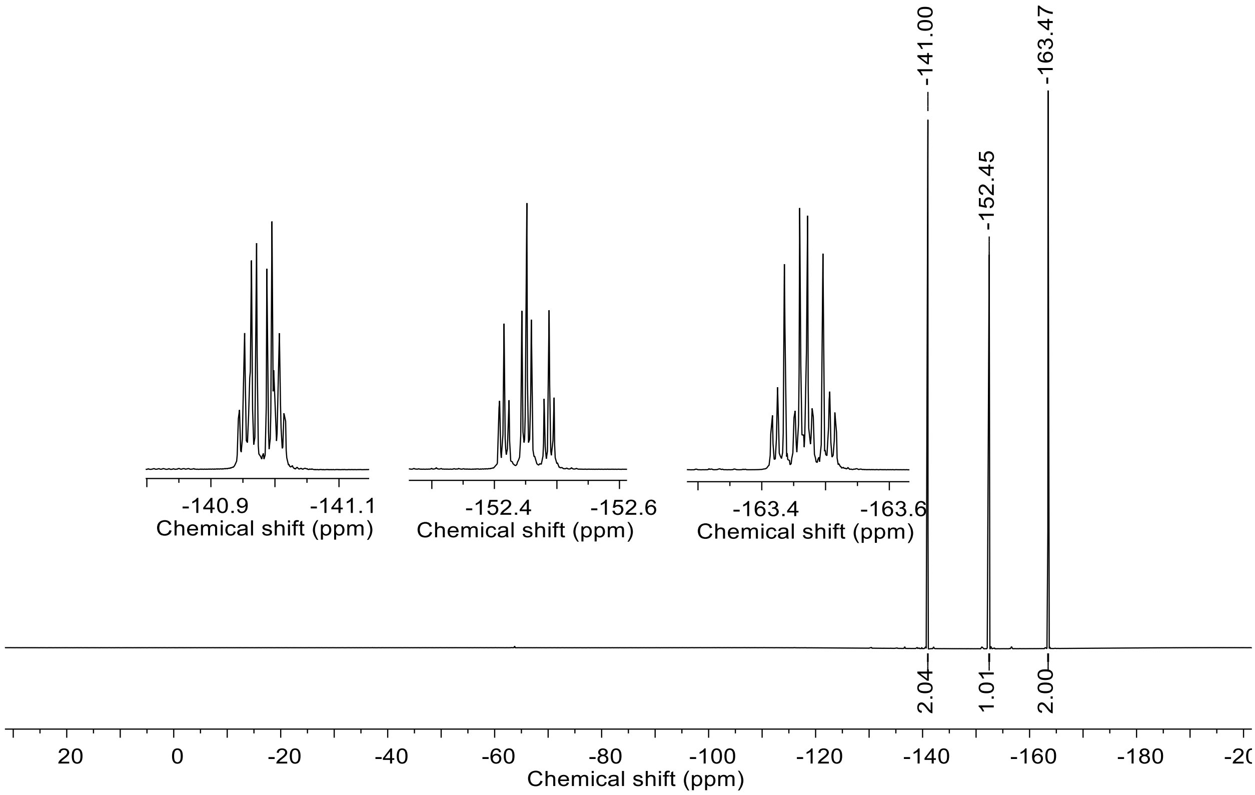

Figure F23. ^{19}F NMR spectrum of 2,3,4,5,6-pentafluorobenzoic acid **2i** (acetone-d_6, 564 MHz).

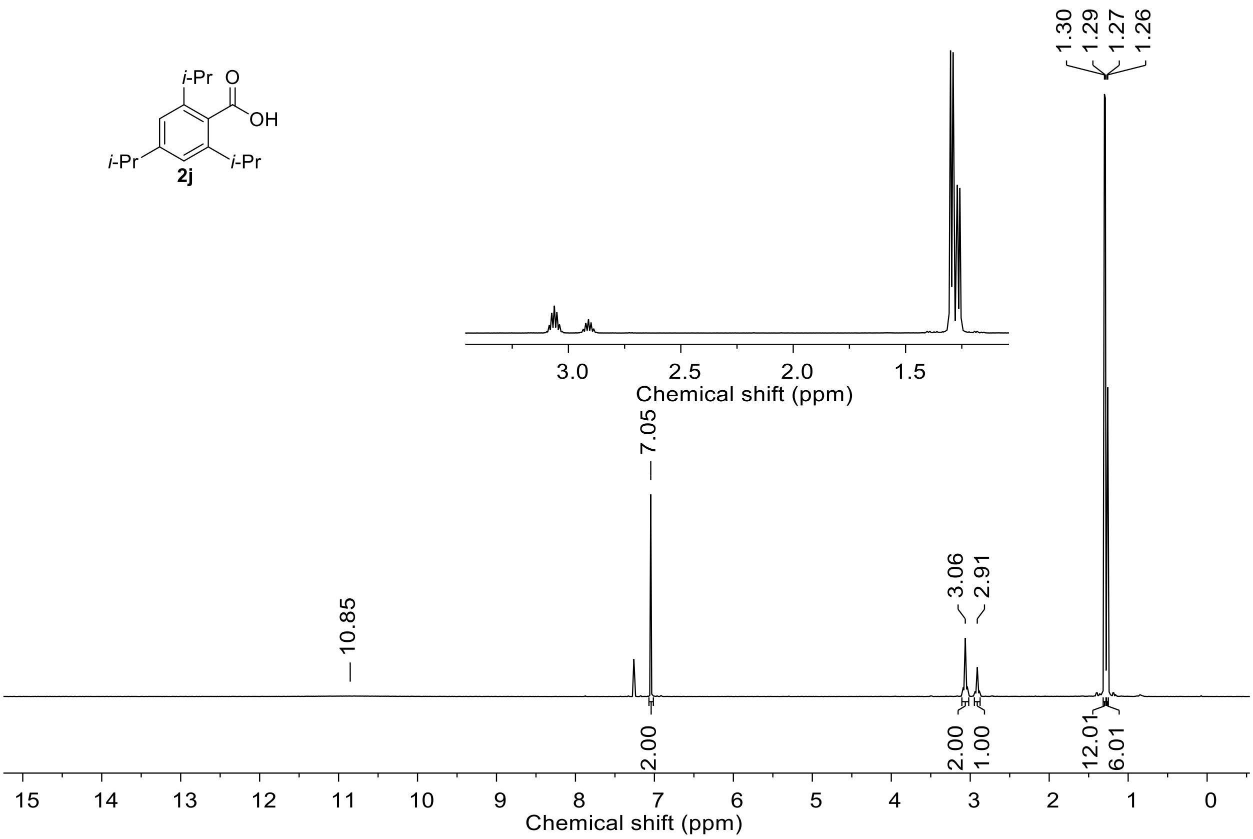

Figure F24. ^{1}H NMR spectrum of 2,4,6-tri-*iso*-propylbenzoic acid **2j** ($CDCl_3$, 600 MHz).

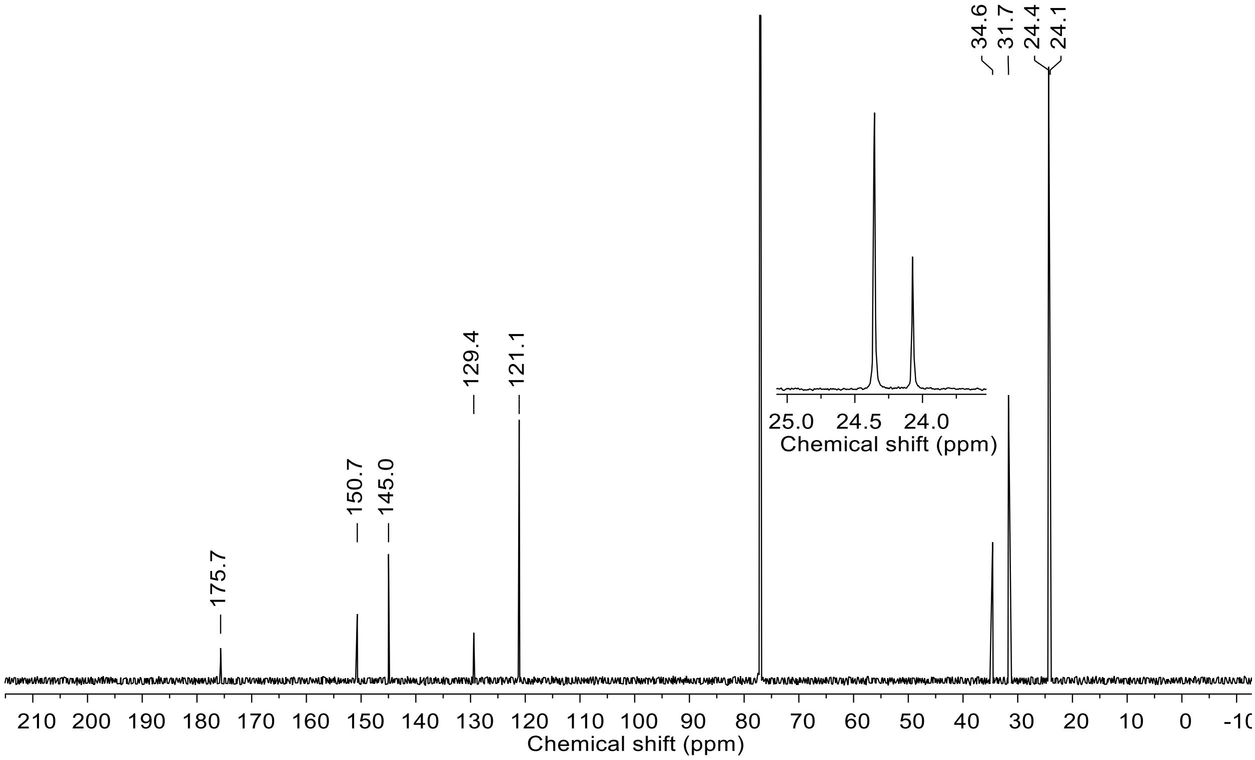

Figure F25. $^{13}C\{^{1}H\}$ NMR spectrum of 2,4,5-tri-*iso*-propylbenzoic acid **2j** ($CDCl_3$, 151 MHz).

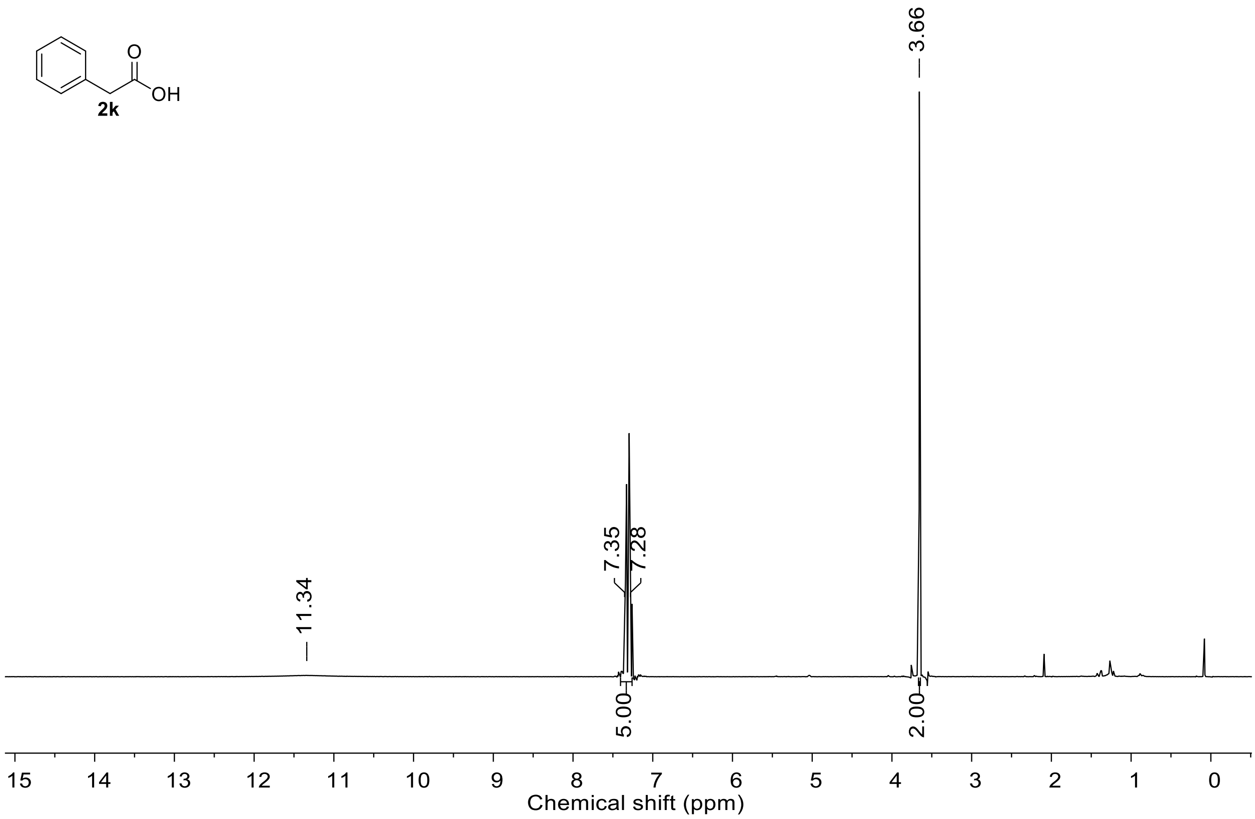

Figure F26. ^{1}H NMR spectrum of 2-phenylacetic acid **2k** ($CDCl_3$, 600 MHz).

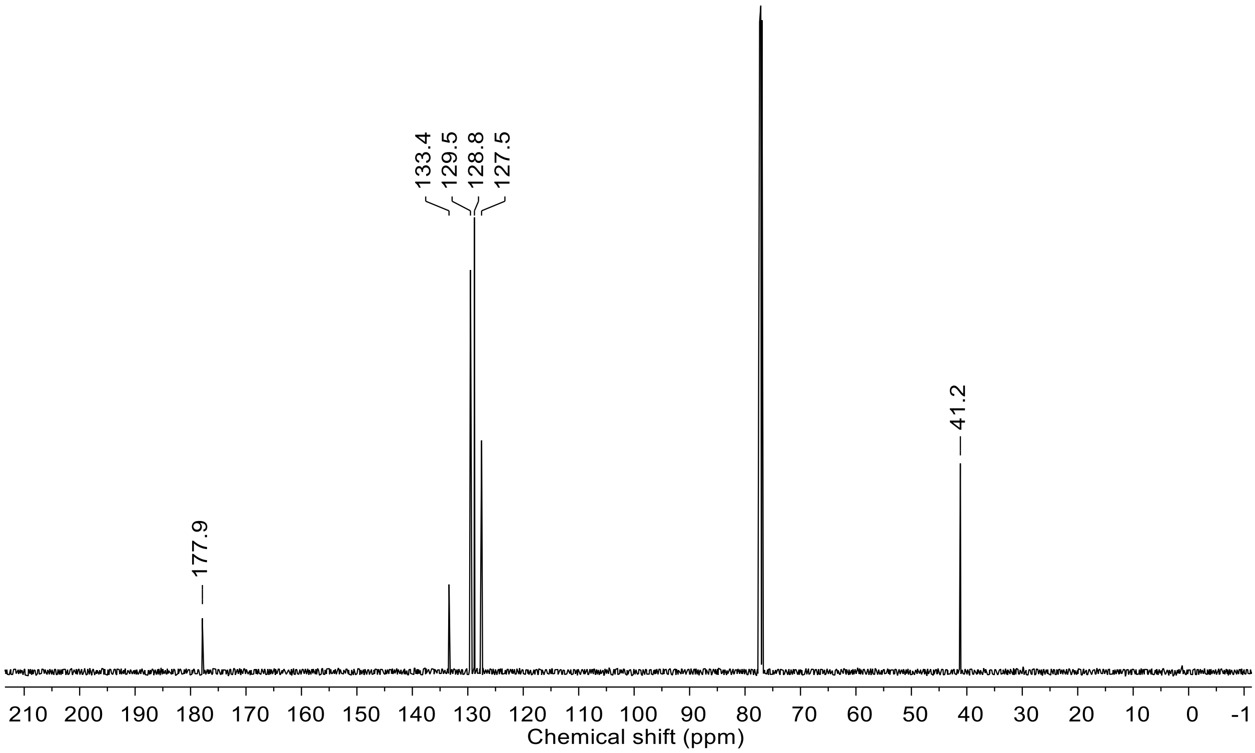

Figure F27. $^{13}C\{^{1}H\}$ NMR spectrum of 2-phenylacetic acid **2k** ($CDCl_3$, 151 MHz).

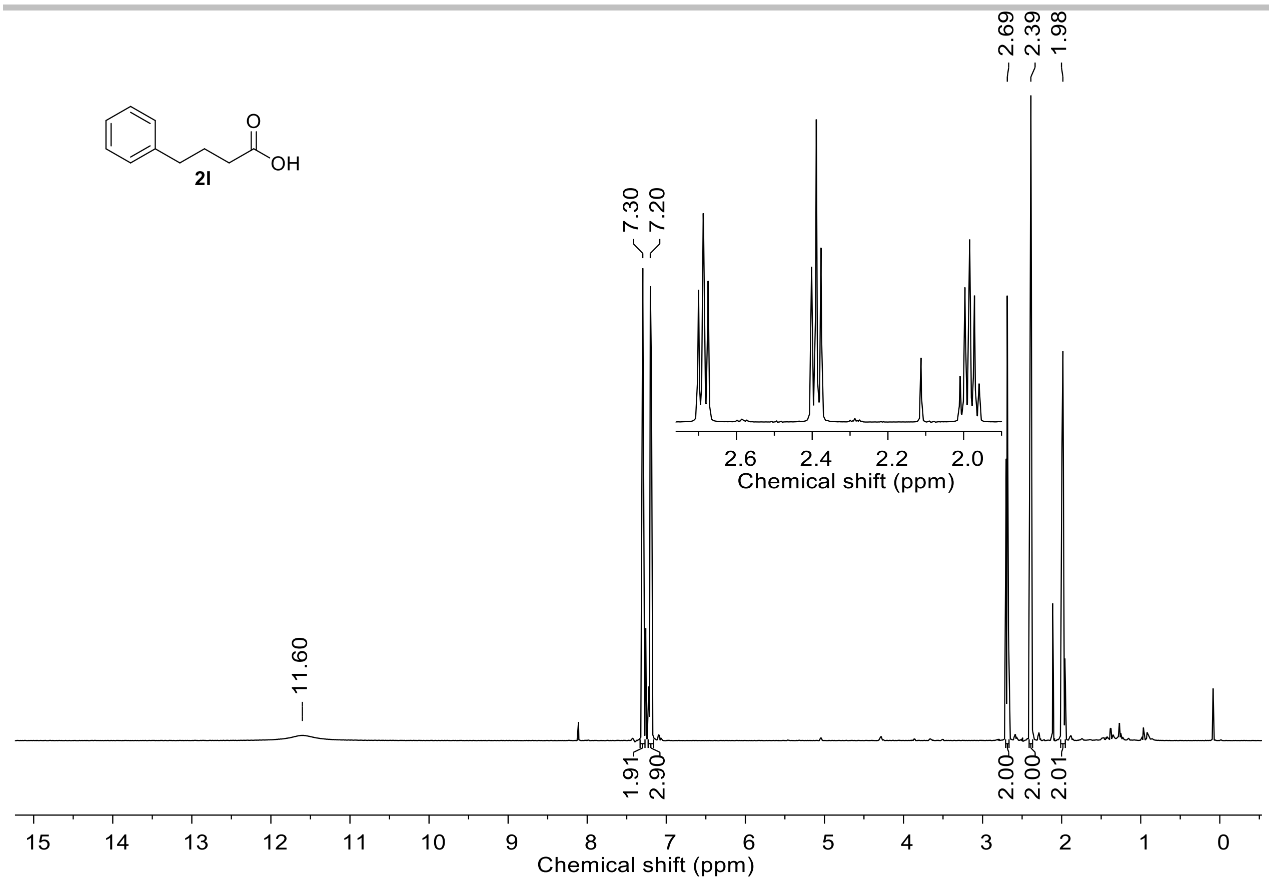

Figure F28. ^{1}H NMR spectrum of 4-phenylbutanoic acid **2l** ($CDCl_3$, 600 MHz).

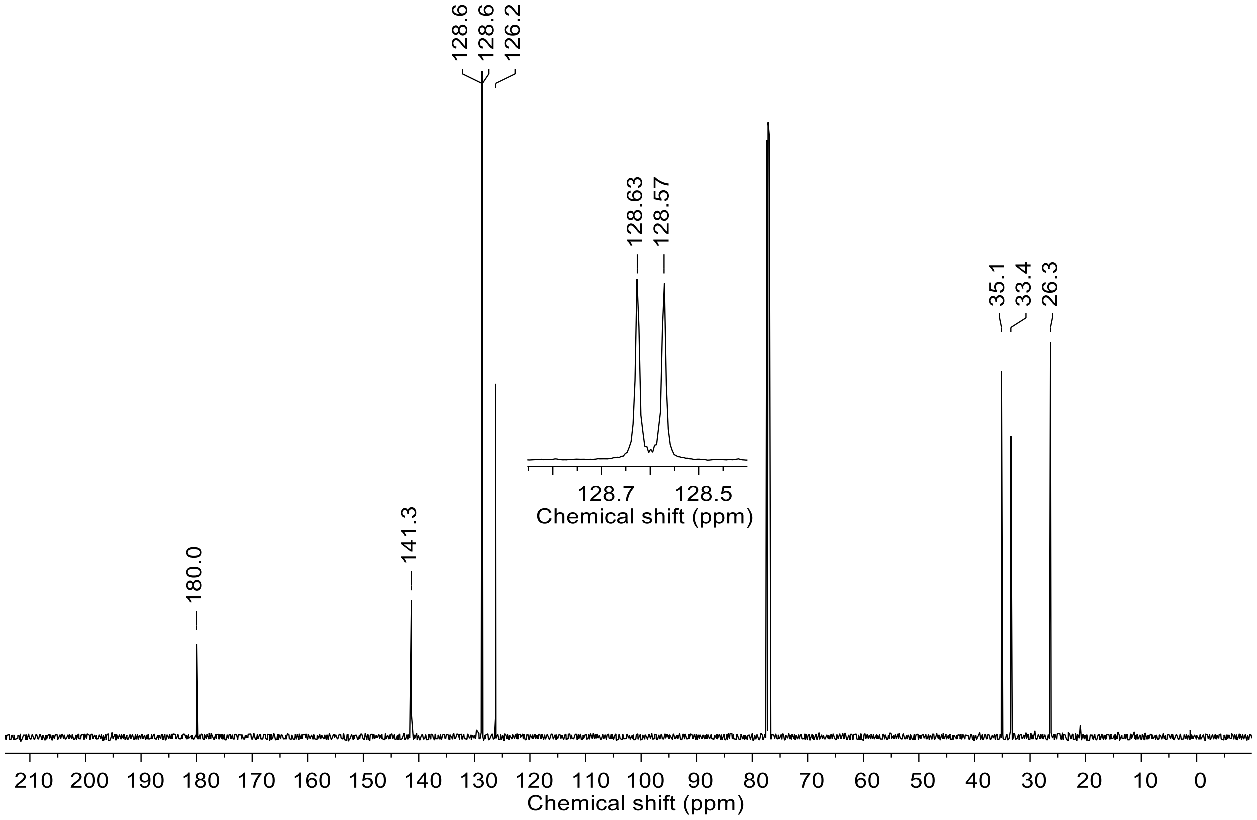

Figure F29. $^{13}C\{^{1}H\}$ NMR spectrum of 4-phenylbutanoic acid **2l** ($CDCl_3$, 151 MHz).

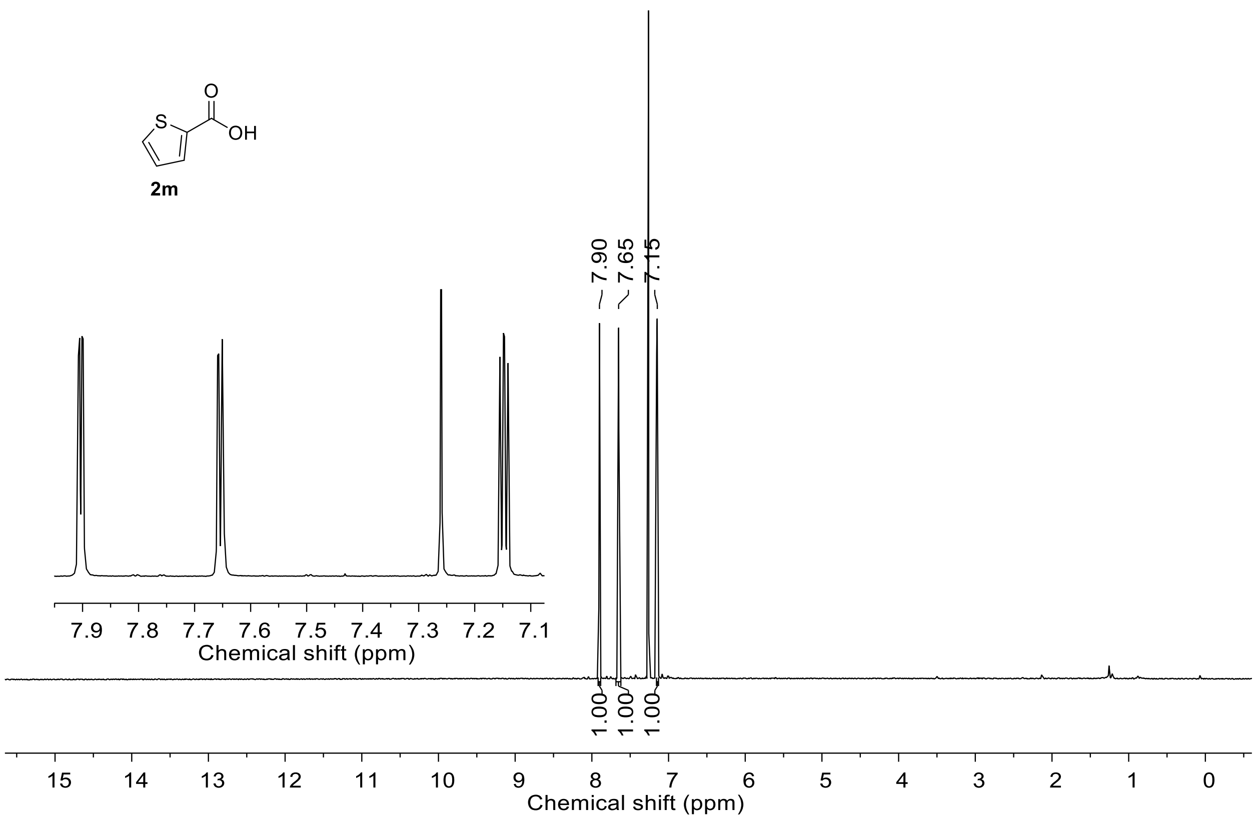

Figure F30. ^1H NMR spectrum of 2-Thiophenecarboxylic acid **2m** ($CDCl_3$, 600 MHz).

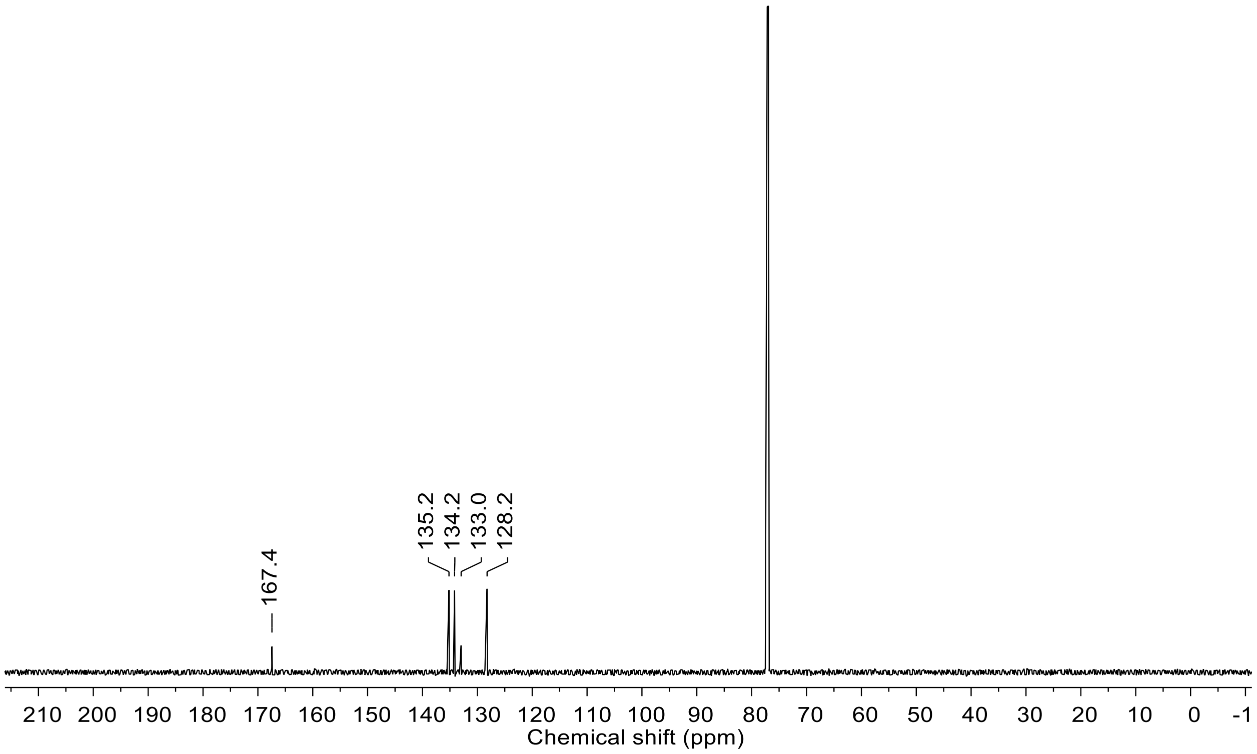

Figure F31. $^{13}C\{^1H\}$ NMR spectrum of 2-Thiophenecarboxylic acid **2m** ($CDCl_3$, 151 MHz).

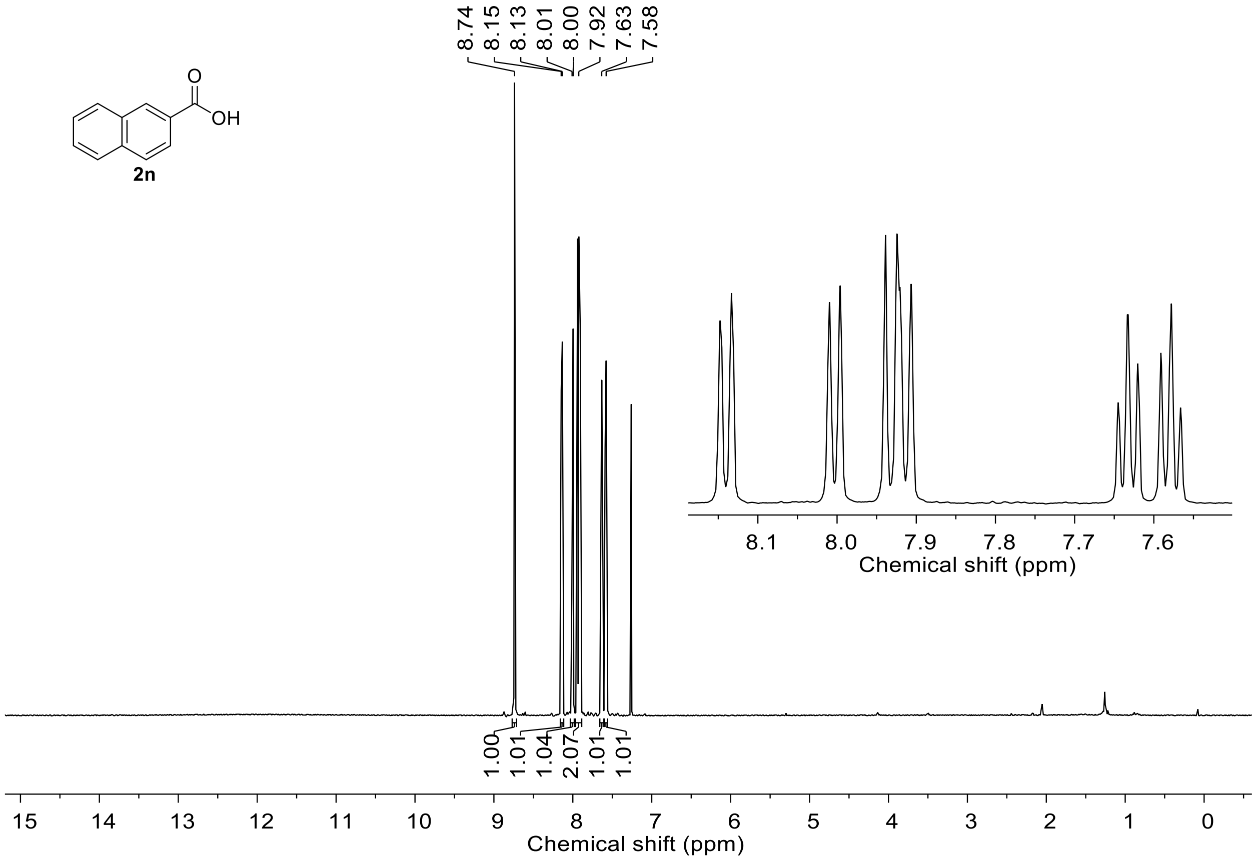

Figure F32. ^{1}H NMR spectrum of 2-naphthoic acid **2n** ($CDCl_3$, 600 MHz).

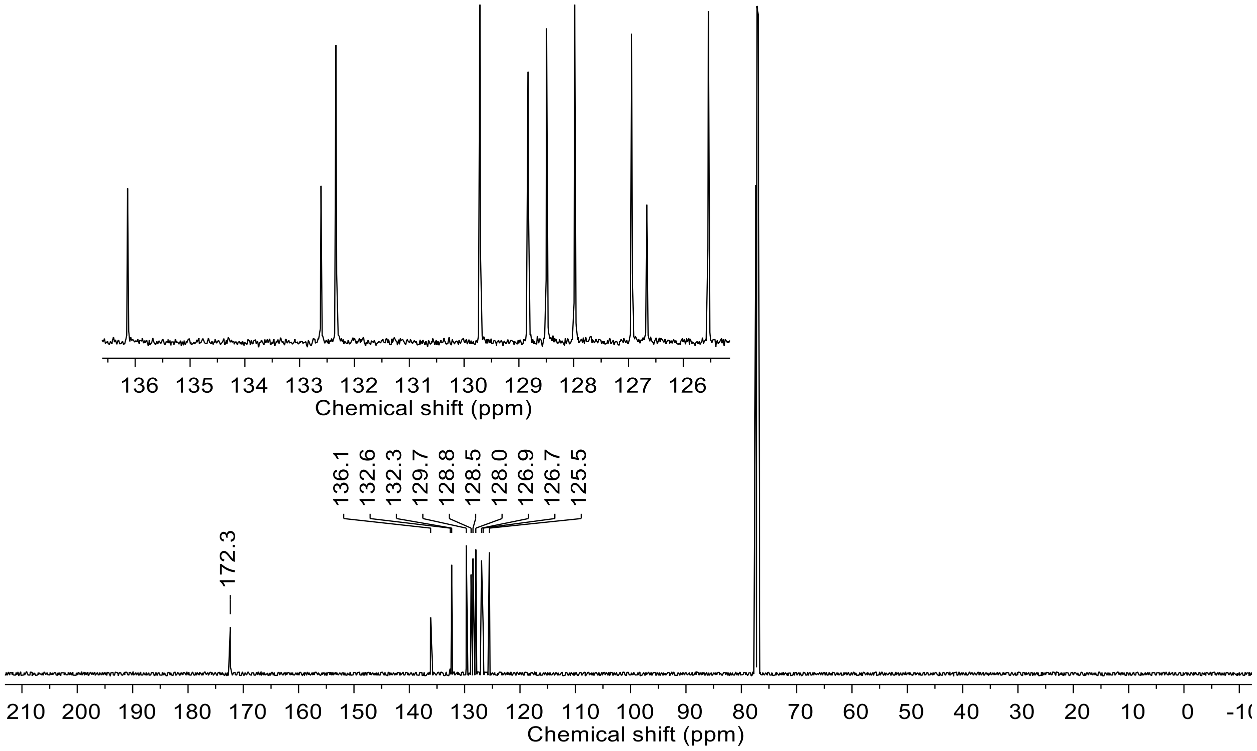

Figure F33. $^{13}C\{^{1}H\}$ NMR spectrum of 2-naphthoic acid **2n** ($CDCl_3$, 151 MHz).

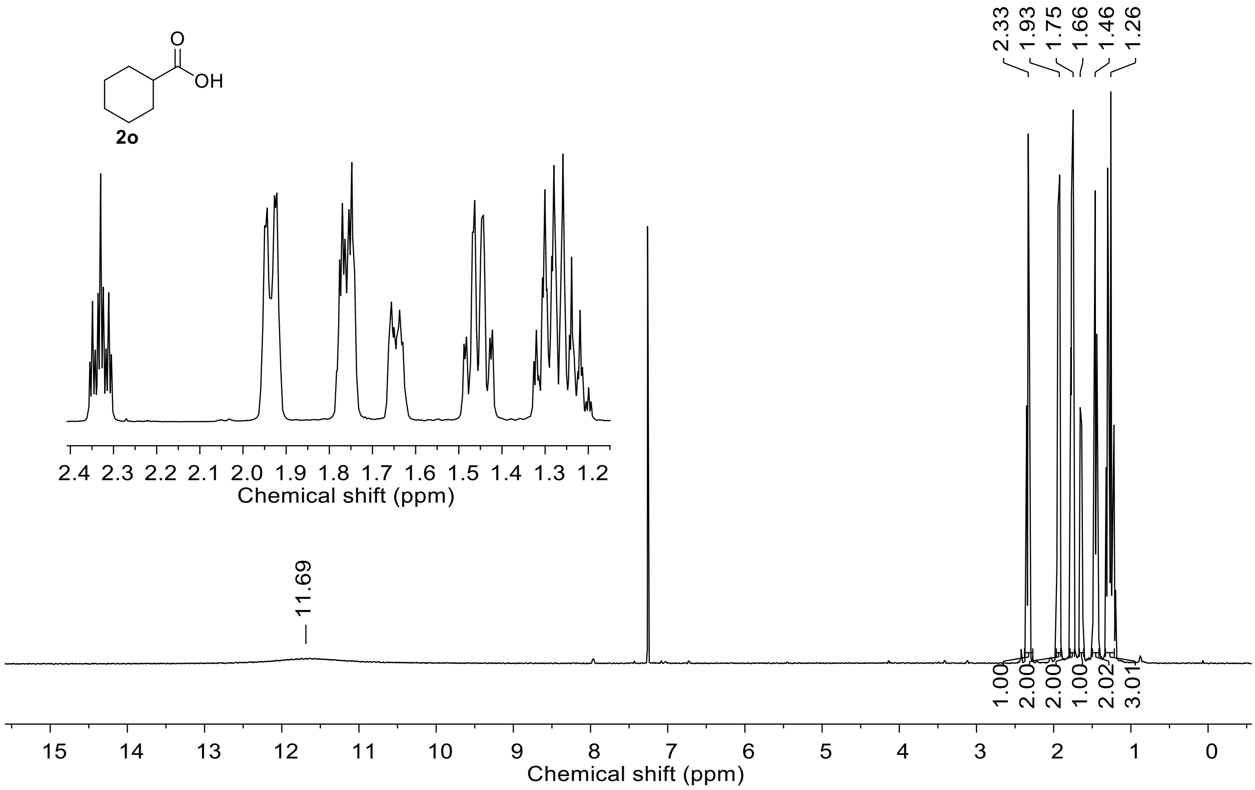

Figure F34. ^{1}H NMR spectrum of Cyclohexylcarboxylic acid **2o** ($CDCl_3$, 600 MHz).

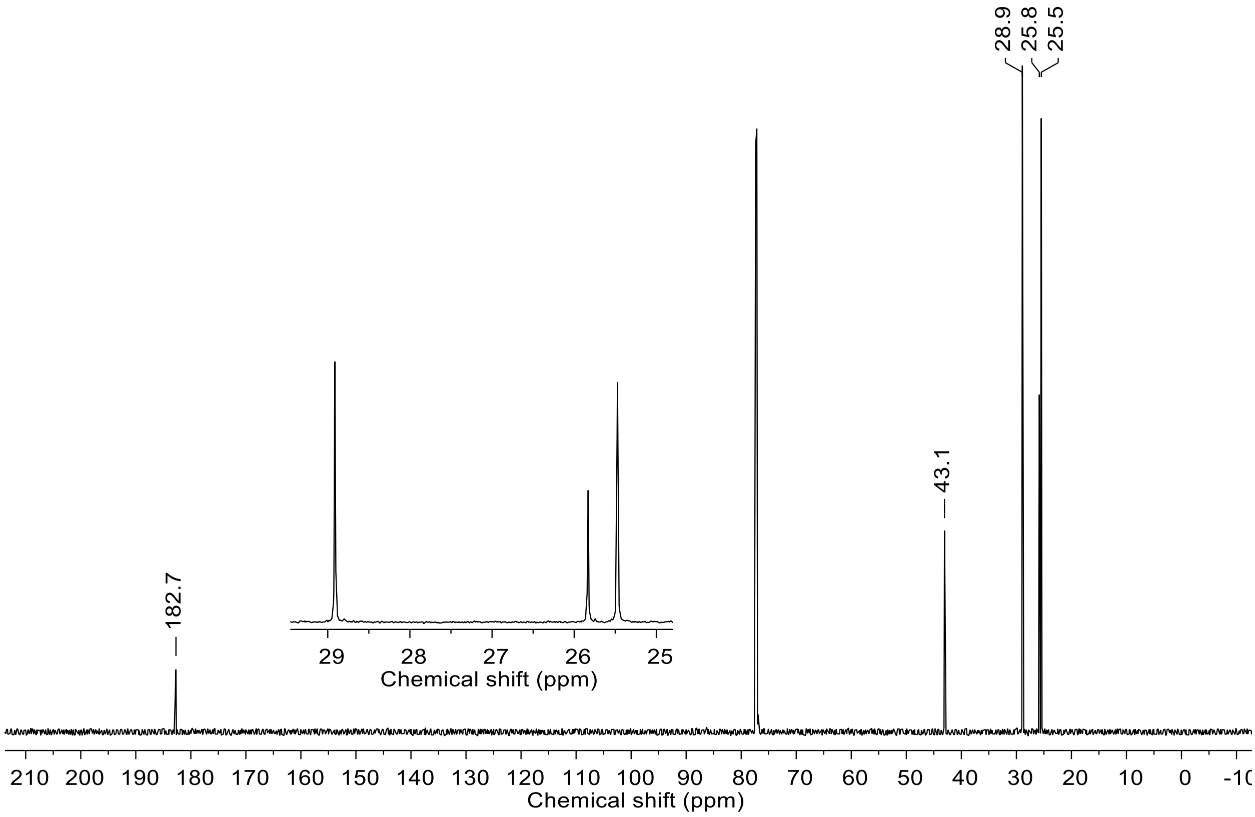

Figure F35. $^{13}C\{^{1}H\}$ NMR spectrum of Cyclohexylcarboxylic acid **2o** ($CDCl_3$, 151 MHz).

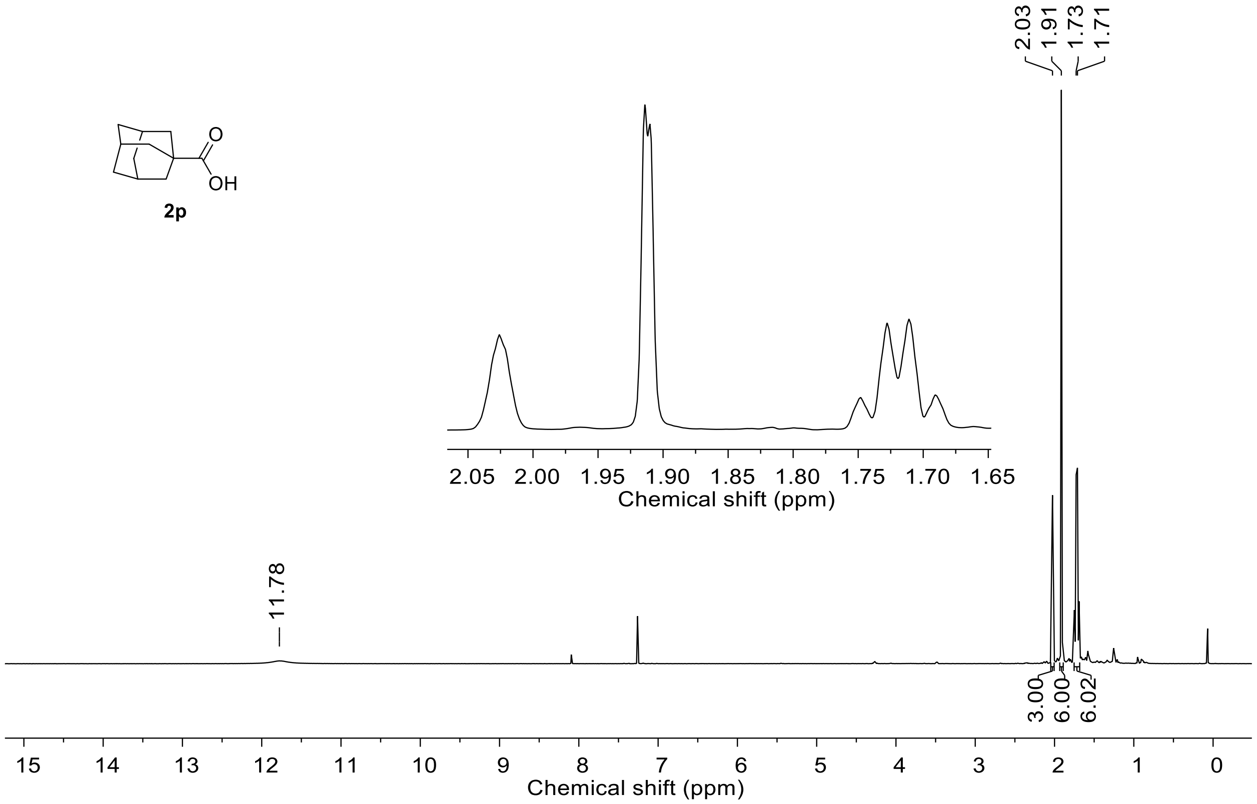

Figure F36. ^{1}H NMR spectrum of 1-adamantylcarboxylic acid **2p** ($CDCl_3$, 600 MHz).

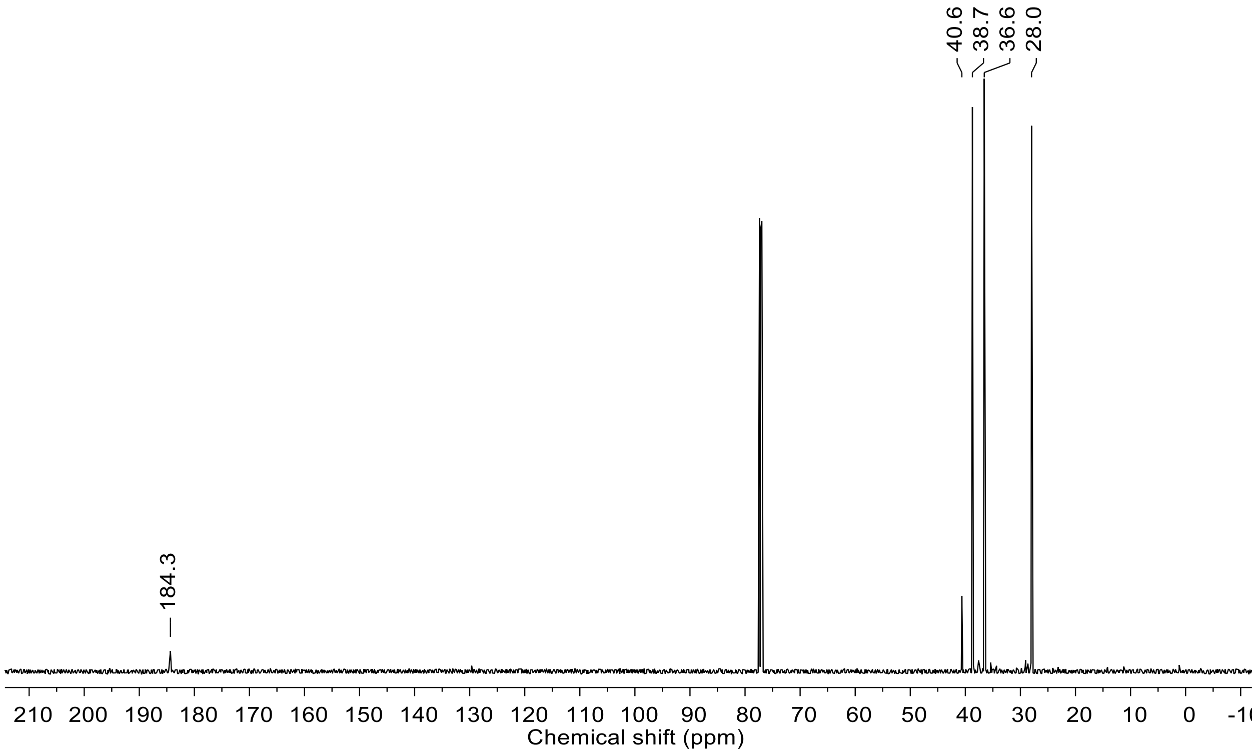

Figure F37. $^{13}C\{^1H\}$ NMR spectrum of 1-adamandtylcarboxylic acid **2p** ($CDCl_3$, 151 MHz).

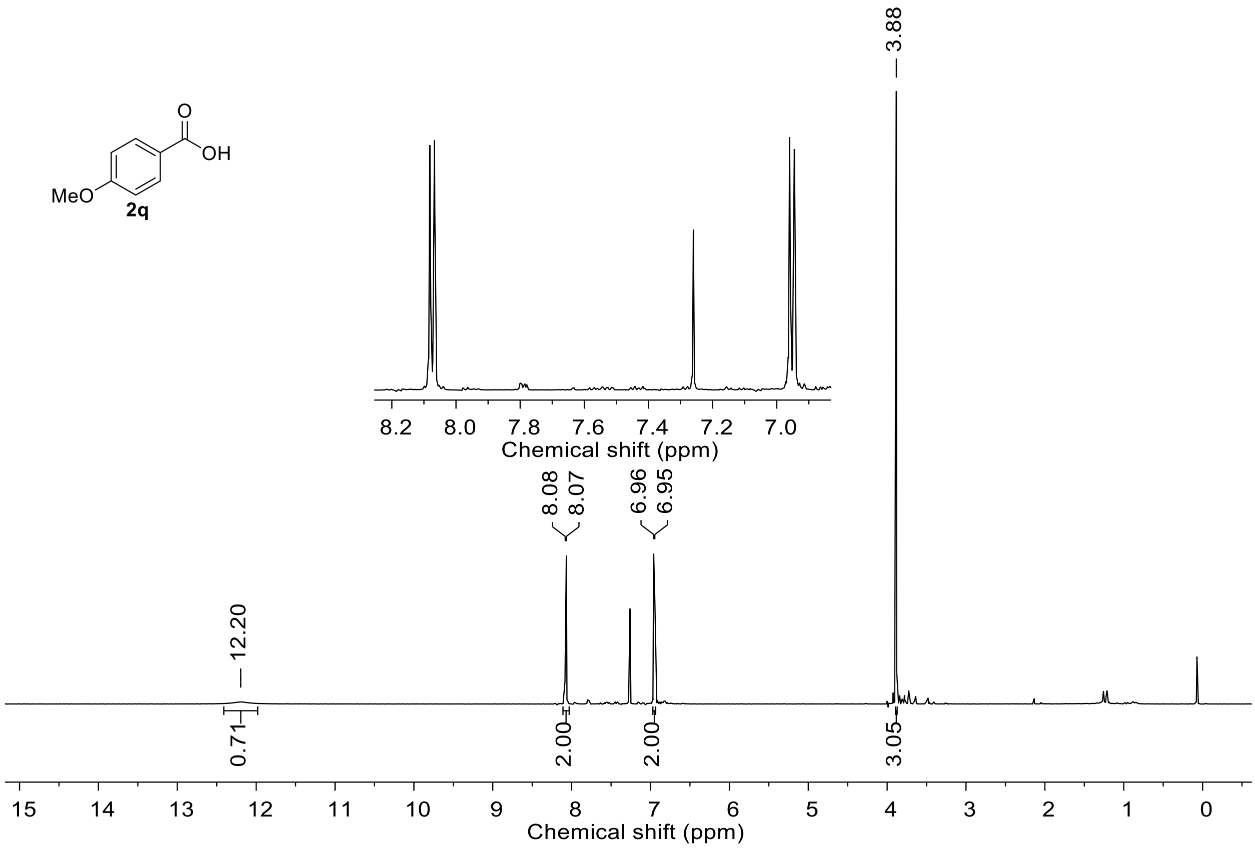

Figure F38. ^{1}H NMR spectrum of 4-methoxybenzoic acid **2q** ($CDCl_3$, 600 MHz).

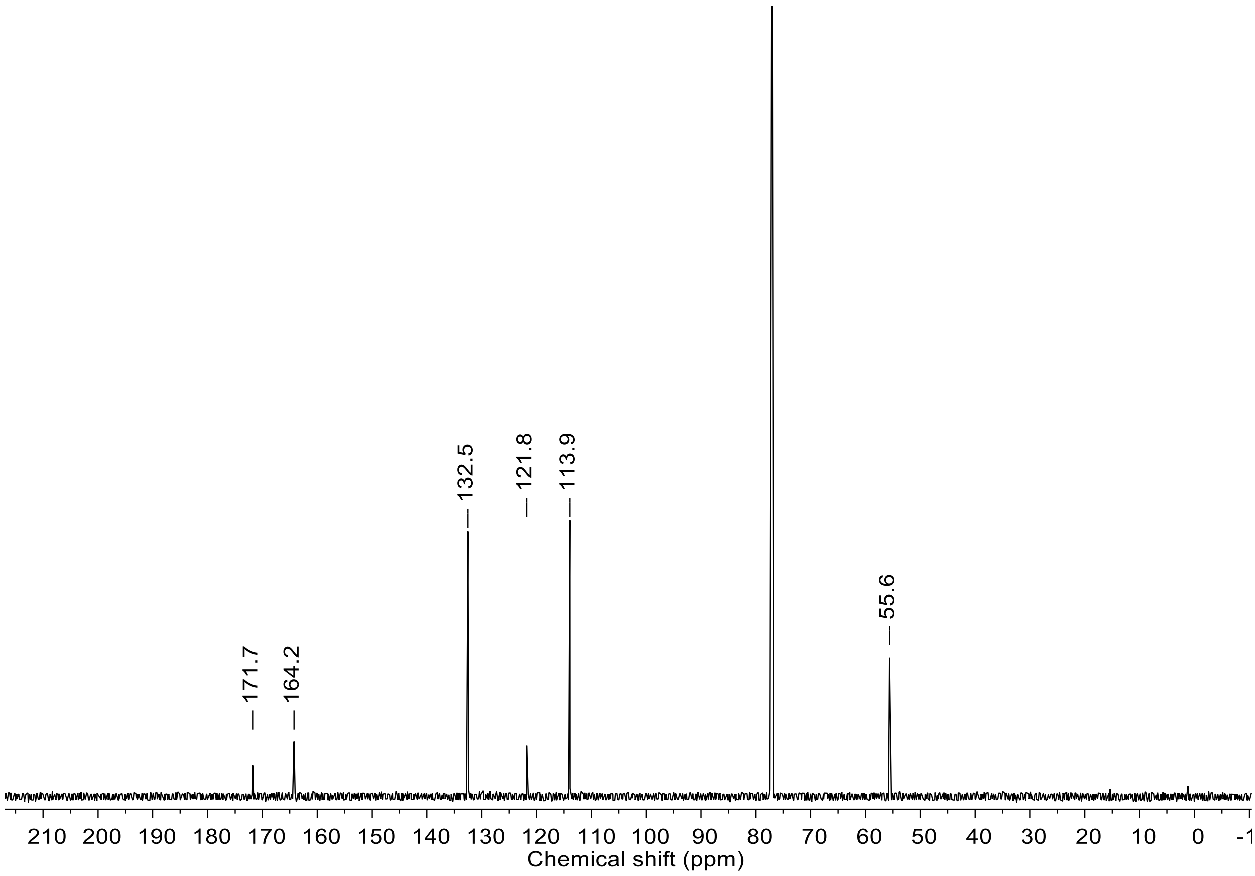

Figure F39. $^{13}C\{^{1}H\}$ NMR spectrum of 4-methoxybenzoic acid **2q** ($CDCl_3$, 151 MHz).

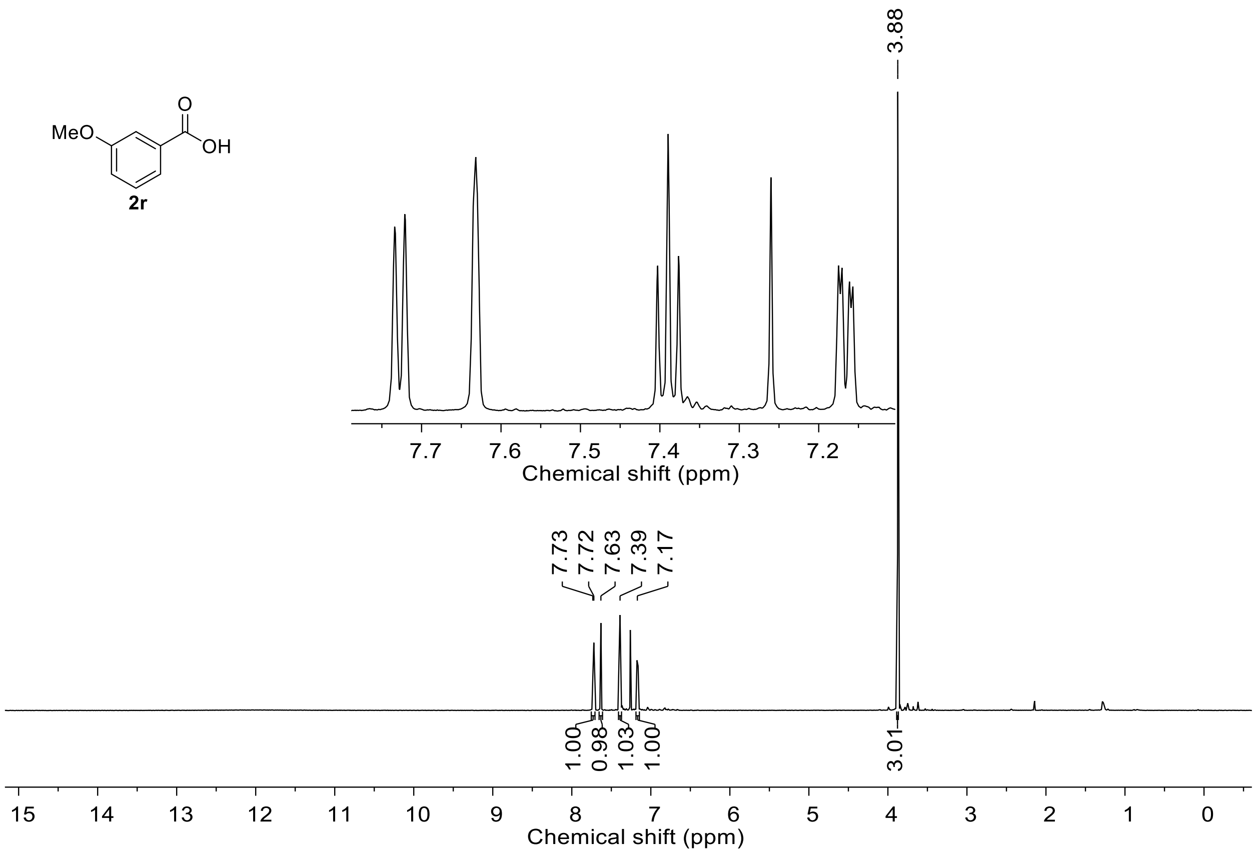

Figure F40. ^{1}H NMR spectrum of 3-methoxybenzoic acid **2r** ($CDCl_3$, 600 MHz).

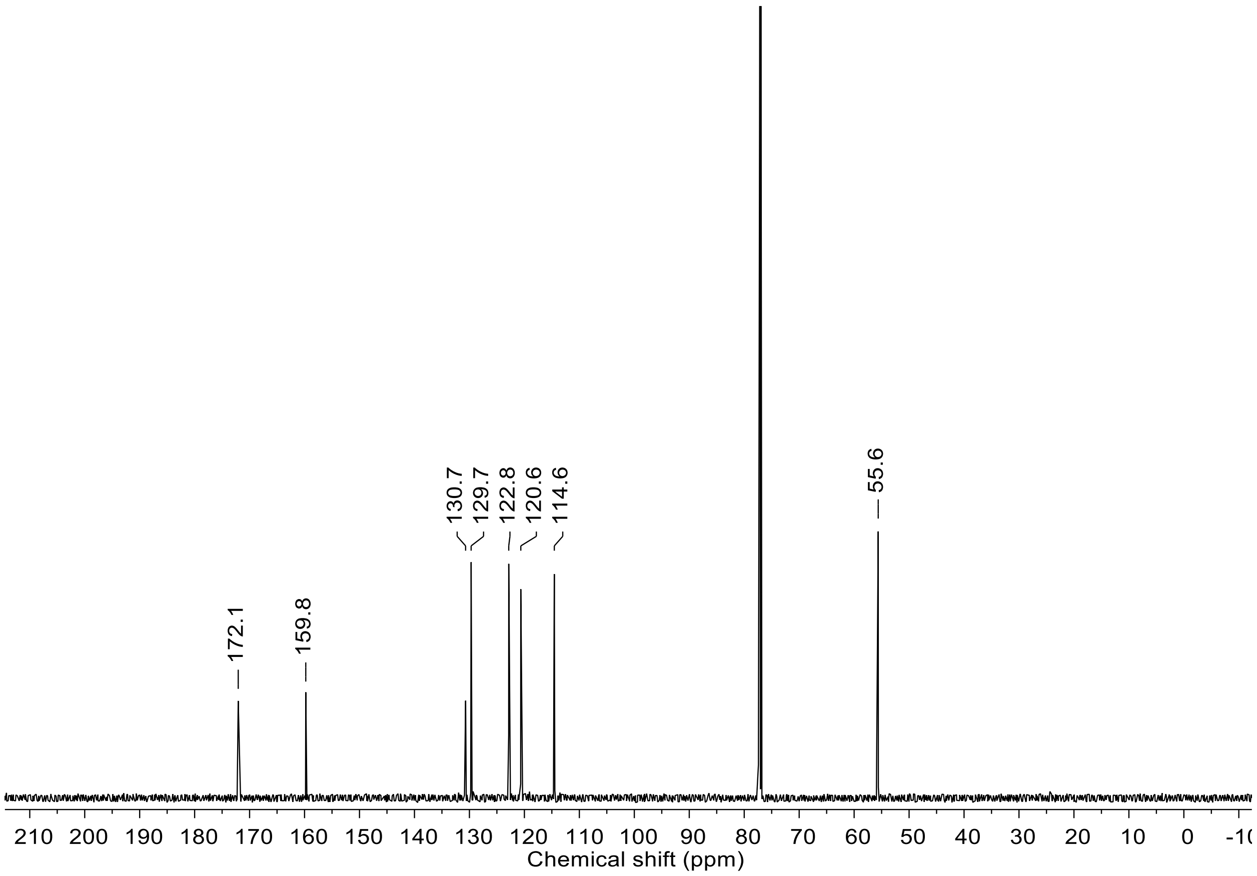

Figure F41. $^{13}C\{^{1}H\}$ NMR spectrum of 3-methoxybenzoic acid **2r** ($CDCl_3$, 151 MHz).

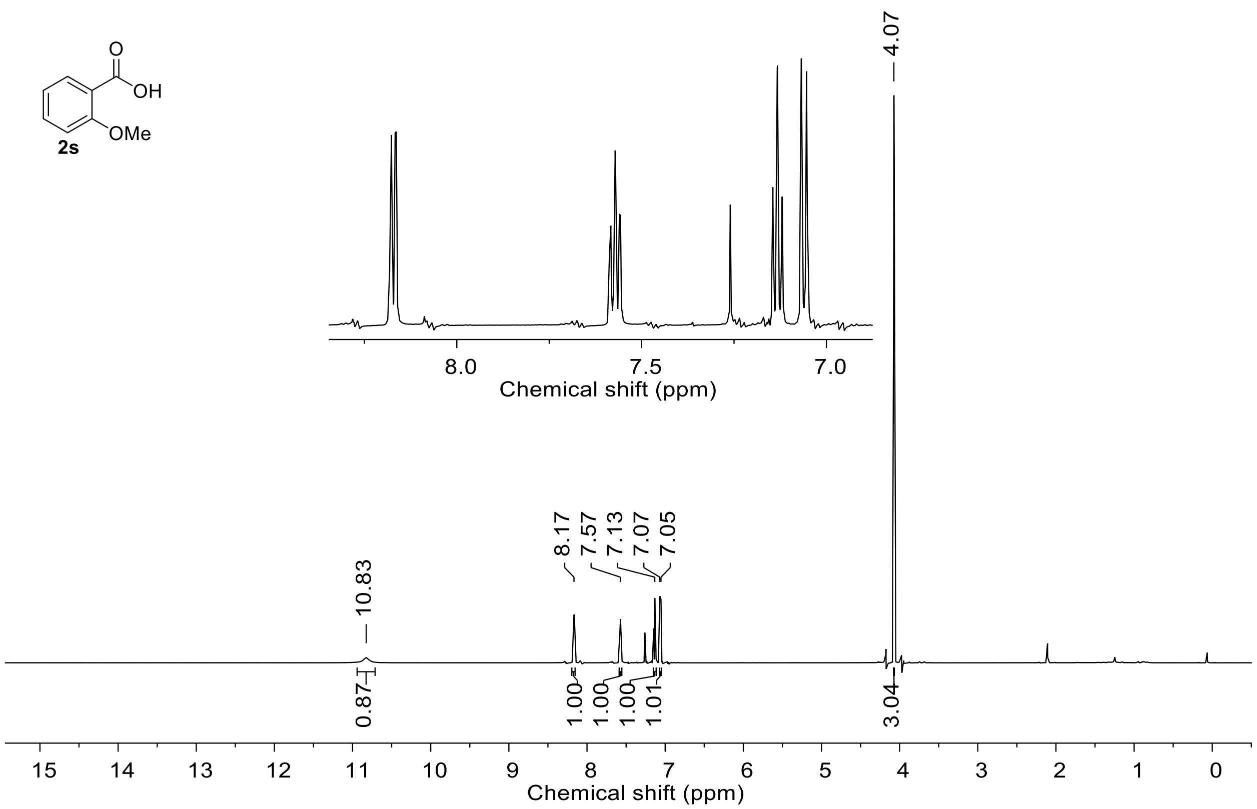

Figure F42. ^{1}H NMR spectrum of 2-methoxybenzoic acid **2s** ($CDCl_3$, 600 MHz).

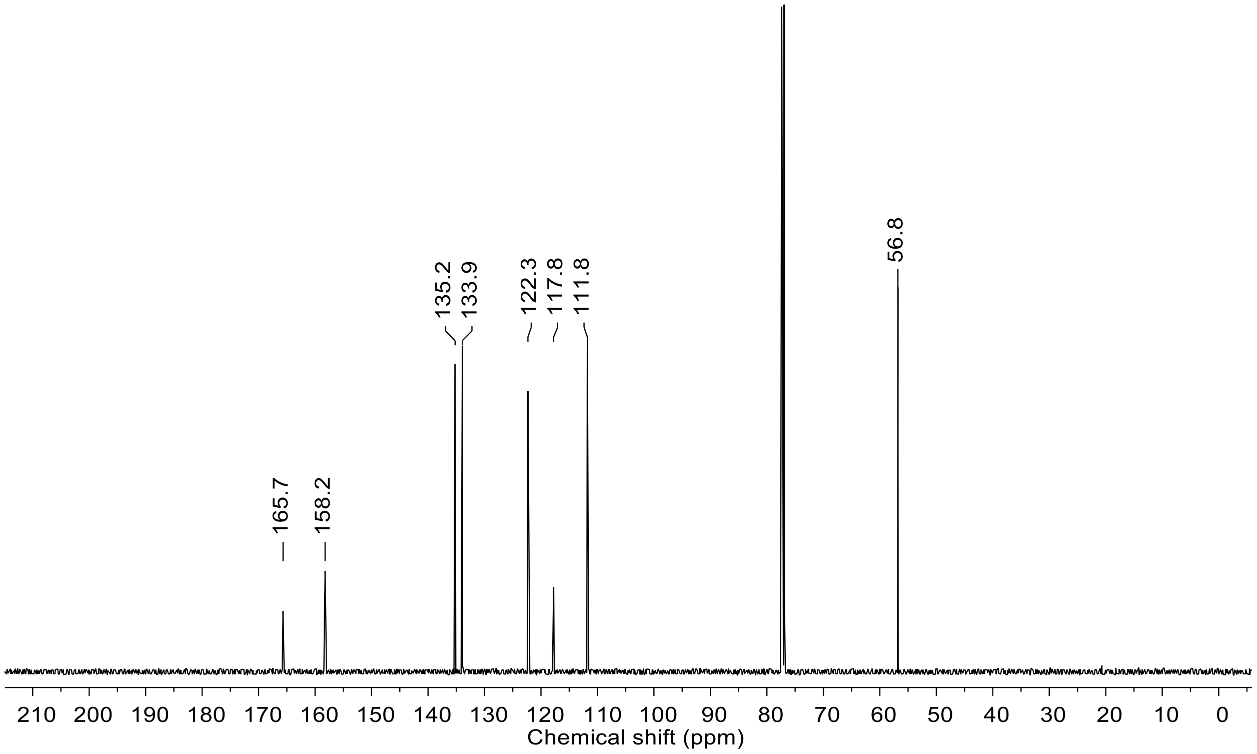

Figure F43. $^{13}C\{^{1}H\}$ NMR spectrum of 2-methoxybenzoic acid **2s** ($CDCl_3$, 151 MHz).

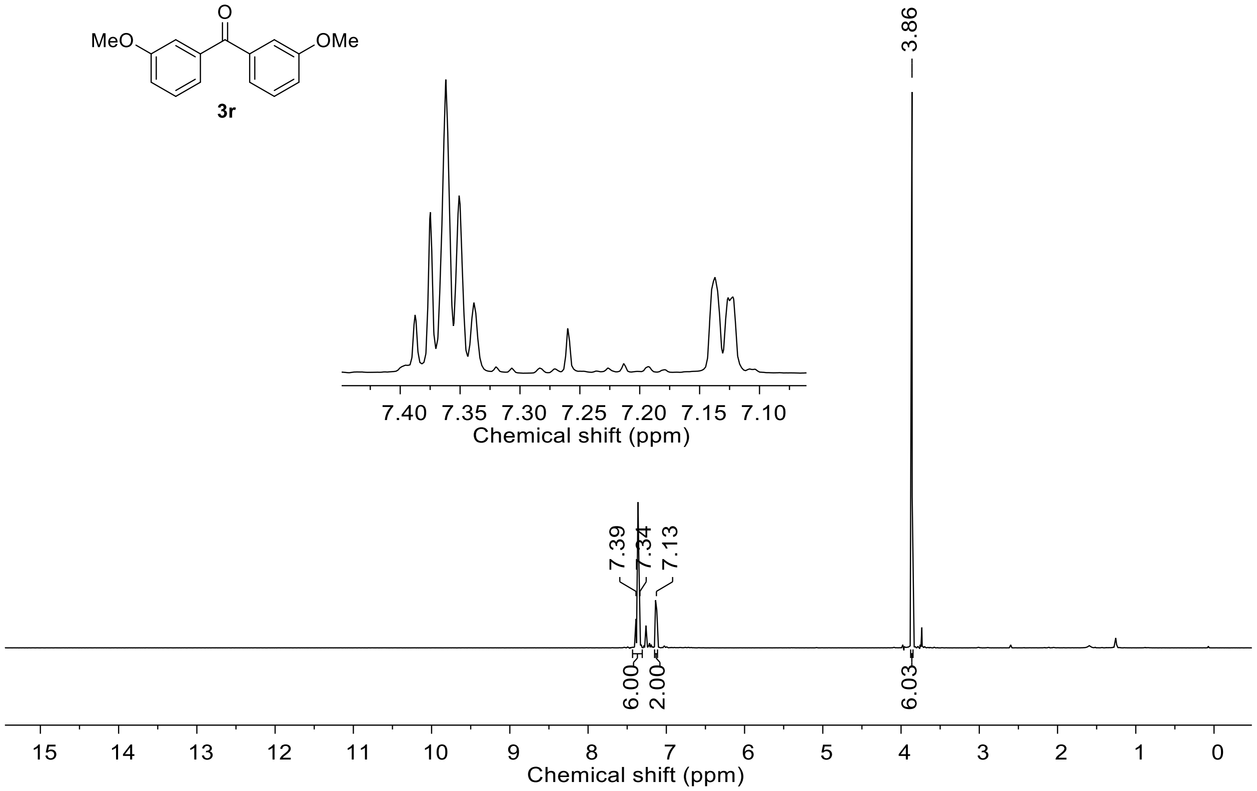

Figure F44. ^{1}H NMR spectrum of 3,3'-dimethoxybenzophenone **3r** ($CDCl_3$, 600 MHz).

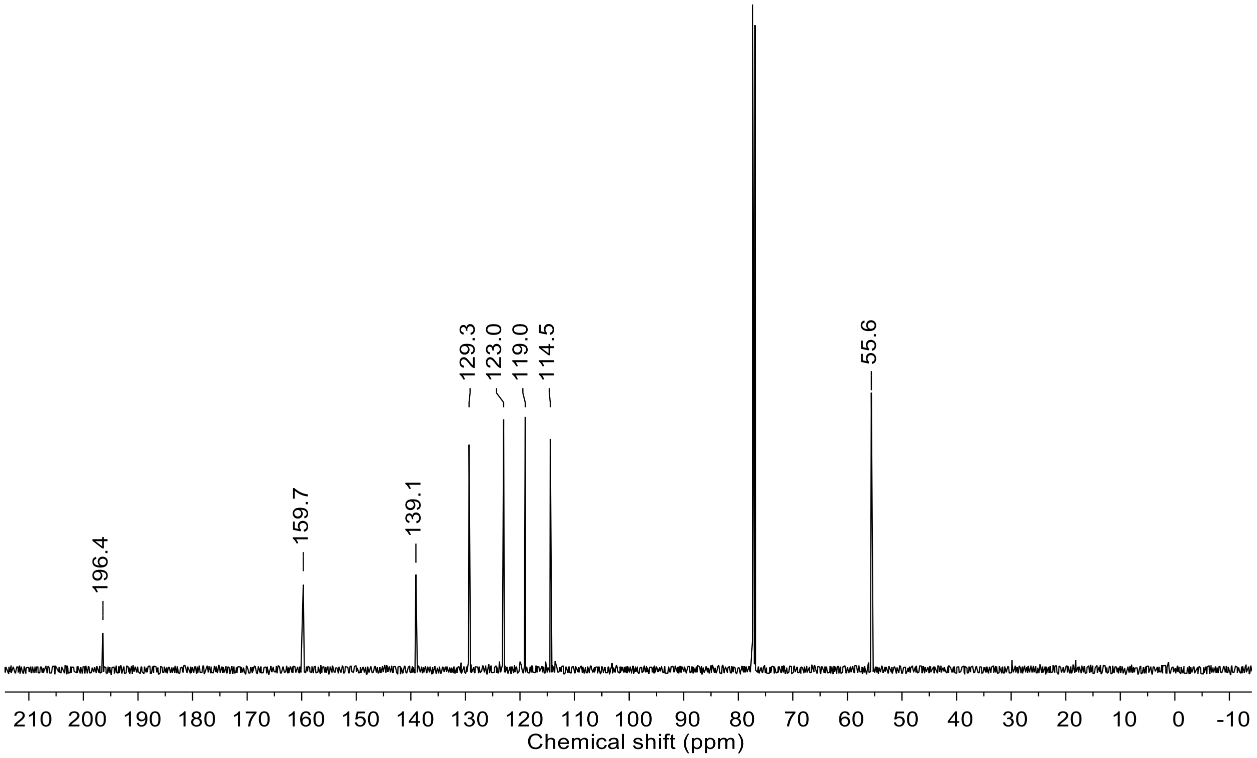

Figure F45. $^{13}C\{^{1}H\}$ NMR spectrum of 3,3'-dimethoxybenzophenone **3r** ($CDCl_3$, 151 MHz).

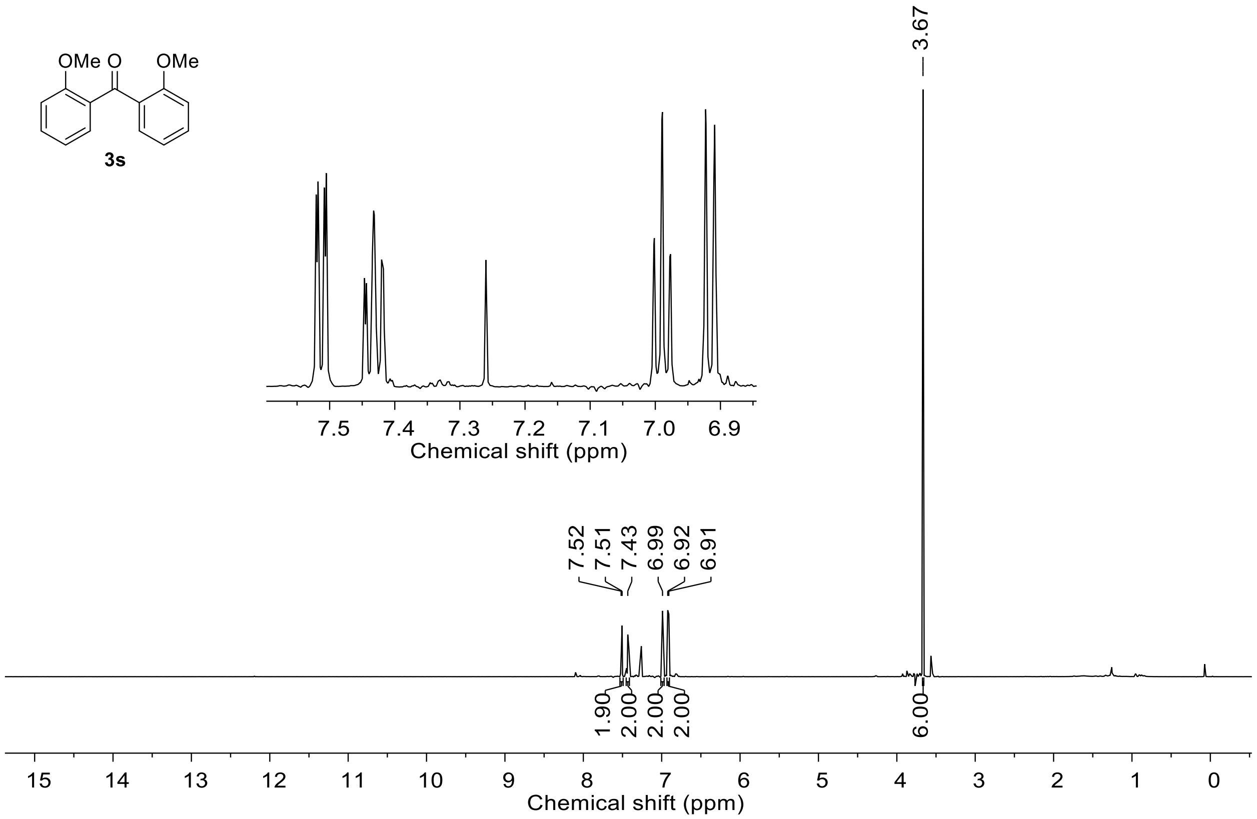

Figure F46. ^{1}H NMR spectrum of 2,2'-dimethoxybenzophenone **3s** ($CDCl_3$, 600 MHz).

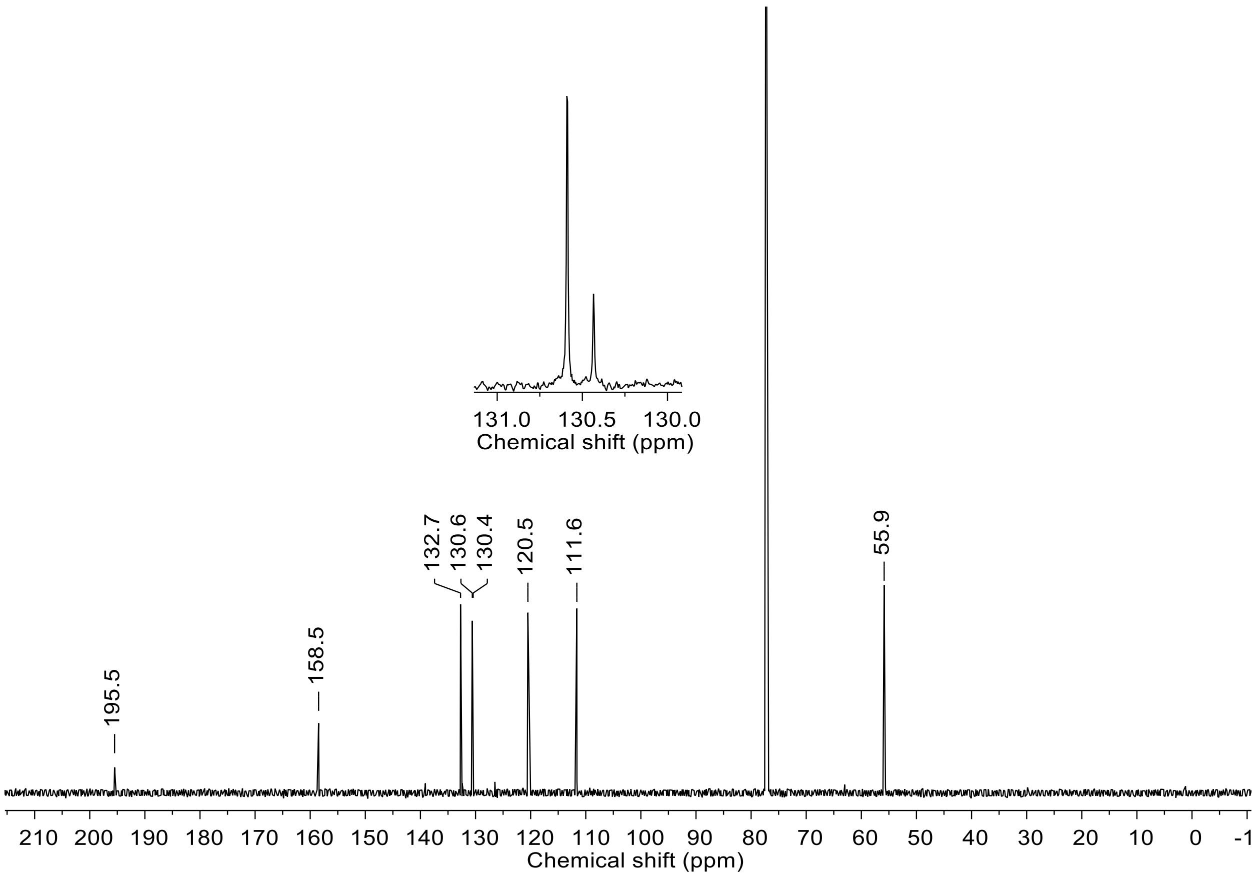

Figure F47. ^{13}C{^{1}H} NMR spectrum of 2,2'-dimethoxybenzophenone **3s** ($CDCl_3$, 151 MHz).

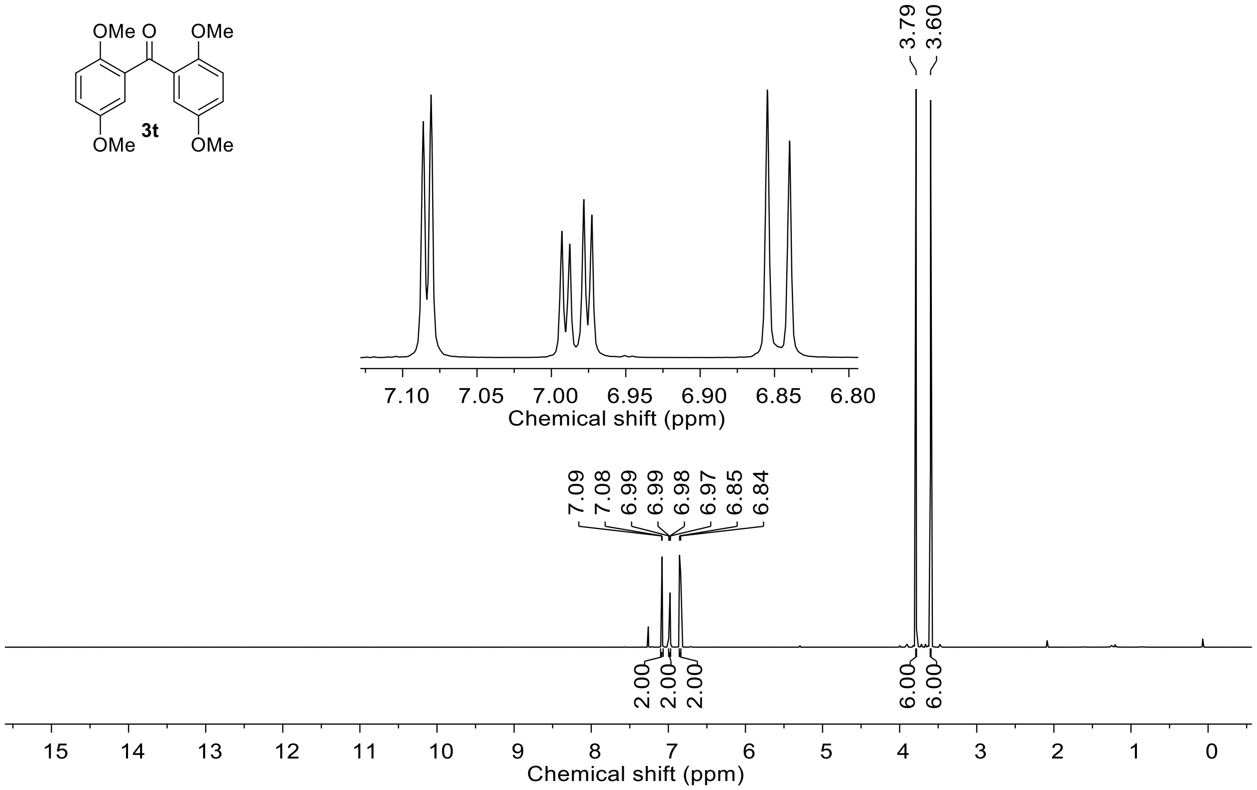

Figure F48. ^{1}H NMR spectrum of 2,2',5,5'-tetramethoxybenzophenone **3t** ($CDCl_3$, 600 MHz).

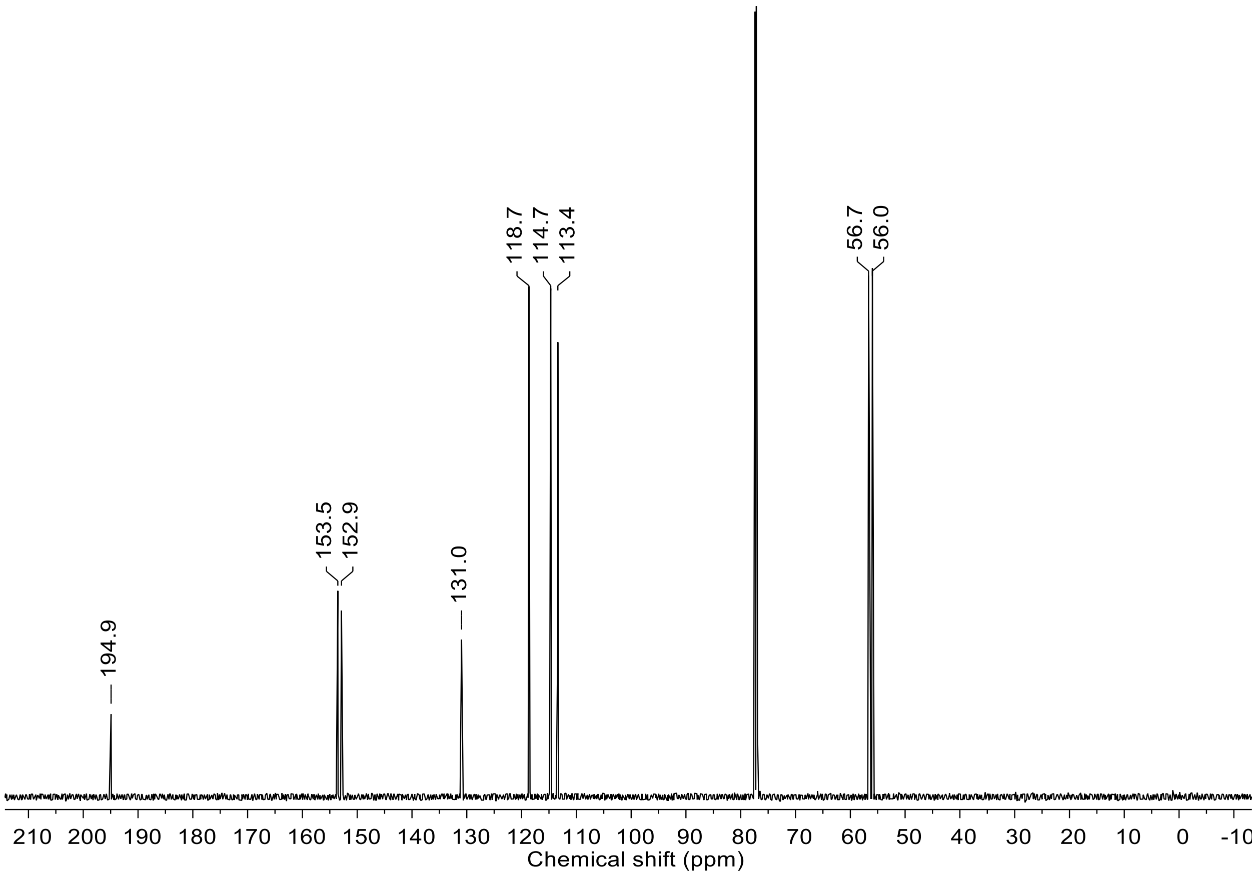

Figure F49. $^{13}C\{^{1}H\}$ NMR spectrum of 2,2',5,5'-tetramethoxybenzophenone **3t** ($CDCl_3$, 151 MHz).

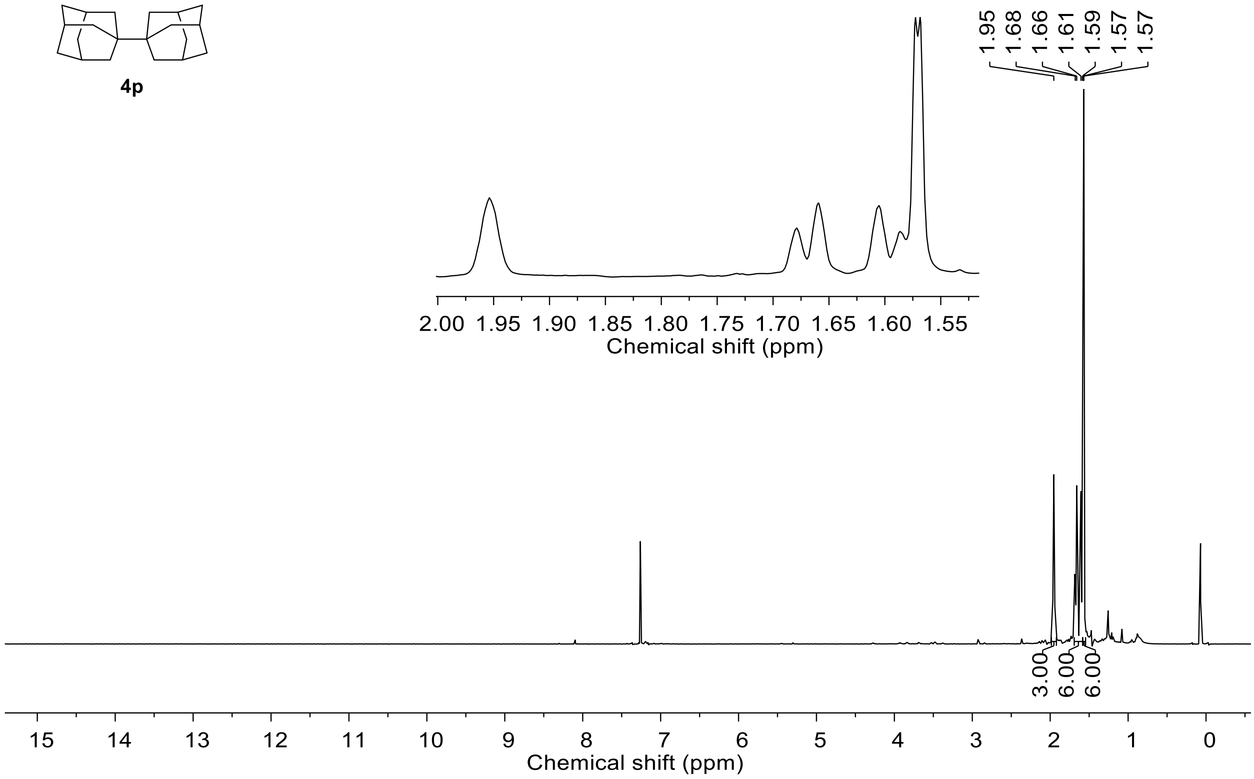

4p

Figure F50. ^{1}H NMR spectrum of Biadamantane **4p** ($CDCl_3$, 600 MHz).

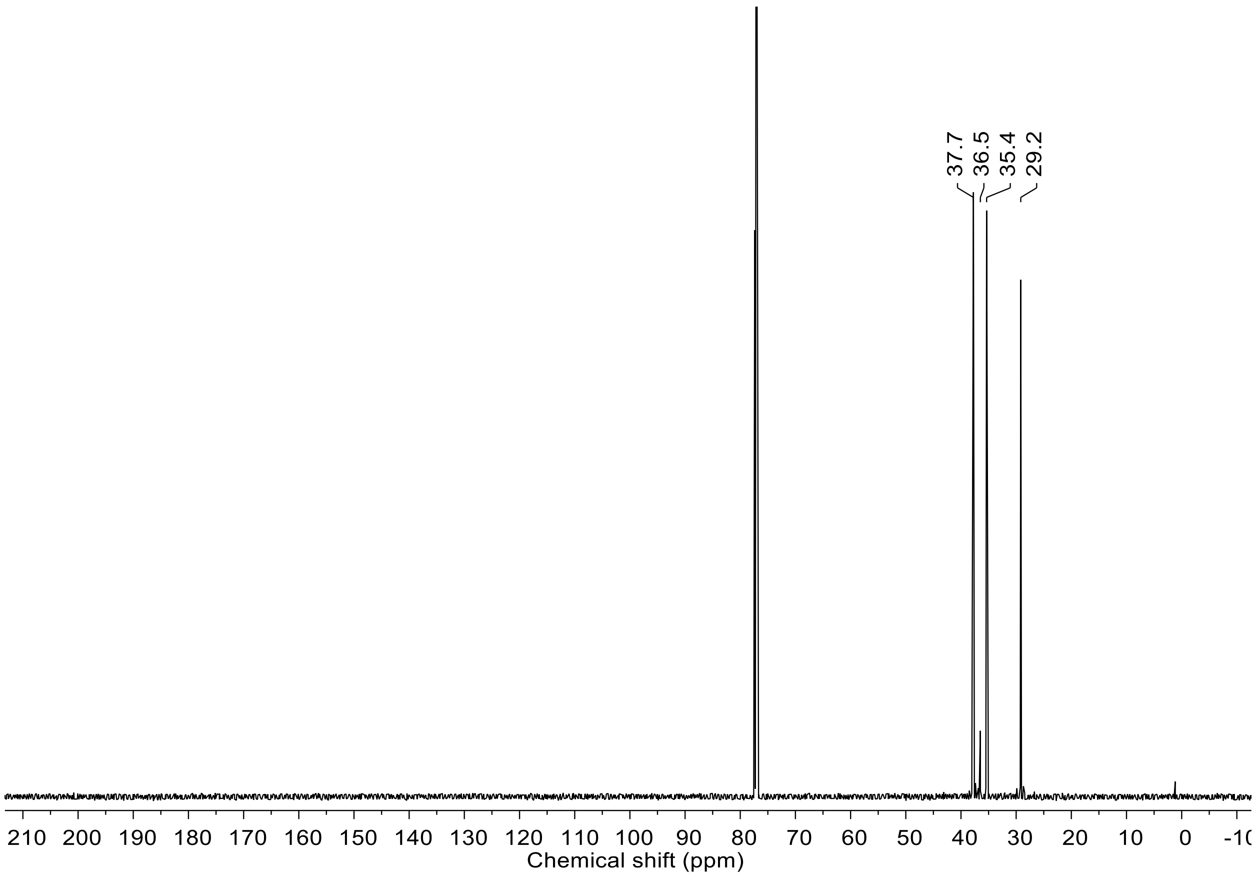

Figure F51. $^{13}C\{^{1}H\}$ NMR spectrum of Biadamantane **4p** ($CDCl_3$, 151 MHz).
